# Cross-Cultural Comparison (13 Countries) of Consumers’ Willingness to Eat Specific Insect Powders in Five Food Types

**DOI:** 10.3390/foods14050841

**Published:** 2025-02-28

**Authors:** Suyeon Park, Edgar Chambers, Jeehyun Lee

**Affiliations:** 1 Department of Food Science and Nutrition, College of Human Ecology, Pusan National University, Busan 46241, Republic of Korea; qkrtndus1224@pusan.ac.kr; 2 Center for Sensory Analysis and Consumer Behavior, Kansas State University, Manhattan, KS 66506, USA; eciv@ksu.edu

**Keywords:** edible insect, cross-culture, powder form, consumer, perception

## Abstract

By 2050, the world’s population will rise to 9 billion, which implies that it is necessary to double protein production. We should consider more sustainable, alternative forms of protein. A solution to this is the use of insects, which offer high levels of protein and require less water than poultry, pork, and beef production. The objective of this study was to evaluate 13 countries’ consumer perceptions regarding the willingness to eat specific types of insects as powdered ingredients in five food types. An online survey was conducted using Check All That Apply (CATA) to assess consumer perceptions across 13 countries. Approximately 630 consumers in each country were surveyed (total n > 8100). The CATA data were analyzed using Cochran’s Q test, which showed highly significant differences among countries. The willingness to eat insects varied by country, food type, and insect species. The results of this study can be used to understand consumers’ perceptions of insects and offer an indicator that can be used when developing insect-containing foods in the future.

## 1. Introduction

The current global food systems have been subjected to uncertainties and pressures due to population growth, urbanization, and climate change [1,2]. By 2050, the world’s population of humans is expected to reach nine billion [3]. However, the populations of all European countries and many Asian countries (Japan, China, etc.) are decreasing. Two-thirds of EU countries are expected to have smaller populations in 2050 than in 2019 [4], and, in Japan, the number of babies born every year has been decreasing for decades [5]. Despite the controversy over population growth, the fact that the global total population is growing remains unchanged, and the negative impacts of animal-based protein consumption have led consumers to search for alternative sources of protein, particularly due to fears about sustainability and environmental impacts [6,7,8,9,10].

Insects have been found to be an excellent source of high-quality protein and can be produced more sustainably than traditional livestock, as scientists have established [11,12]. Insect breeding was suggested by Meyer-Rochow [13] to offer another option to solve global protein shortages. Their environmental and nutritional benefits have been widely discussed, which has resulted in a marked increase in consumer interest in recent years [14]. Insects provide a significant amount of energy (fats and protein) and are less demanding on land and water than farm animals, which is a benefit [15,16,17]. High fertility, small production space requirements, and lower levels of greenhouse gas emissions during production are just a few of the advantages of them [18,19]. Furthermore, they are rich in essential amino acids, are excellent sources of polyunsaturated fatty acids [20], and are also abundant in iron, selenium, zinc, and numerous B vitamins [17].

Currently, the global obesity pandemic is one of the most serious problems threatening public health due to its close association with several diseases [21]. It has been mentioned that many people eat a nutritionally poor diet, and the consumption of insects and insect derivatives, such as insect powder, can have a positive effect in terms of preventing these problems. The chitin derivatives present in insects also exhibit an anti-inflammatory capacity, stimulating immune responses, preventing diabetes, and preventing and managing obesity [22], and the presence of physiologically active compounds has led to effects on hyperlipidemia and fat reduction in humans [23]. Insect powder is produced through the screening, washing, blanching, and drying of insects, followed by grinding them into a powder [24]. This process reduces the moisture content, lowering the likelihood of spoilage and making it easier to use them as an ingredient for processing [24]. Processing insects into powder form is important because, when insects are provided in an invisible powder, consumers’ acceptance increases [25].

Even in communities that traditionally consume edible insects and insect powders, they are still not accepted as widespread foods or ingredients, despite their potential benefits [26,27,28]. Only 7% of US consumers considered insect powder as a natural ingredient, suggesting that insects are not perceived positively as a food ingredient [29]. Insects are regarded as being sources of infectious diseases, toxins, and harmful microbes by most consumers [25]. In addition to food safety issues, the rejection of insect consumption may be attributed to other external factors, such as religion and allergic reactions [30]. Grabowski [31] pointed out that certain insects possess the potential to transmit diseases or cause poisoning, allergies, and physical injuries. For example, they can spread venomous needles or pathogens. According to Cappelli et al. [32], insects are prone to the accumulation and production of chemical pollutants such as heavy metals. In addition to physical factors, the major reasons that consumers are reluctant to eat insects and insect powders are the degree of disgust and unfamiliarity and negative perceptions related to edible insects. It is likely that the aforementioned factors cause consumers to consider edible insects as a high-risk food source, posing major obstacles to the promotion of insect powders in food products [30,33].

Unusual and unappealing foods or ingredients may be more appealing to consumers if they are not visible and are presented in a familiar way [14,34,35]. The initial barriers to consumption could be lowered by developing products in this direction [36]. To effectively reduce negative perceptions and attitudes among consumers, it is necessary to seek a deeper understanding of all barriers and challenges [37]. Despite the growing interest in edible insects/insect powders among foods to replace animal-based protein, the barriers to entry for consumers remain high. Previous studies have emphasized psychological and cultural factors in eating insects. However, cross-cultural studies examining the perceptions of certain insect powders when they are used as ingredients for familiar foods are lacking. Therefore, this study aimed to bridge this gap by evaluating the willingness of consumers in 13 countries to consume specific insect powders when they were contained in five familiar food types. The research hypothesis was that, when it was contained in an invisible powder form in a familiar food type, the willingness to consume would be high. Through this study, it will be possible to develop strategies to encourage the consumption of edible insects, which are not yet consumed widely. Thus, the objectives of this study were to (1) compare the willingness to eat insect powders produced from a range of insects; (2) compare the willingness to eat insect powders in various food types; and (3) determine whether the country of residence impacts the willingness to consume such products.

## 2. Materials and Methods

### 2.1. Schematic Overview of Survey Process

**Figure 1:**
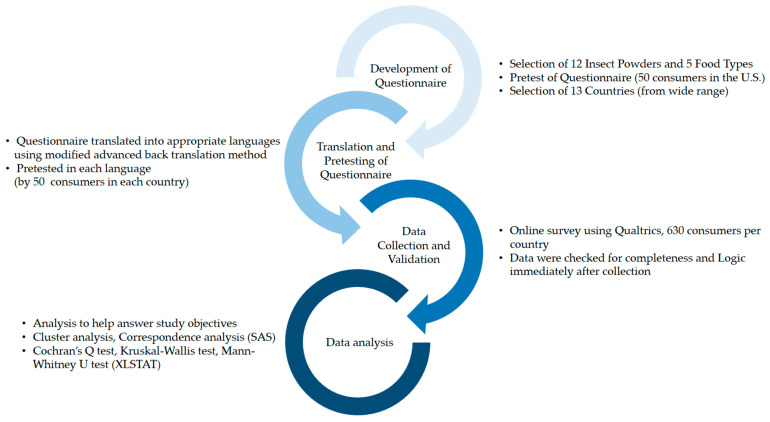
Schematic flow chart of survey process.

A flow diagram of the survey process, from the initial development of the questionnaire to the analysis of the data, is presented in Figure 1, with details given in the text. The objectives of the study led the development of a questionnaire that focused on various insect powders in various food types in multiple countries. The flow diagram includes the main steps in the process, including development, pretesting, translation, data collection, and analysis. The flow diagram facilitates an understanding of the process that we used through a concise, easy-to-understand graphic. This process is widely used in various global studies of consumer behavior related to food and other products.

### 2.2. Participants

Participants were surveyed in a total of 13 countries (Australia, Brazil, China, India, Japan, Mexico, Peru, Russia, South Africa, Spain, Thailand, the United Kingdom (UK), and the United State of America (USA)). These countries were chosen because they represent a broad range of consumers, including respondents across all continents, in countries that have typically eaten insects and those that have not; they also encompassed diets that ranged from vegetarian to frequent meat consumption and countries where the regulation of insects or insect-based ingredients varies. Approximately 630 consumers from each country (8191 consumers in total) (Table 1) were included. Gender (female, male) and age were balanced across all countries, and the ages of the participants were divided into three groups: 18–34 years old, 35–54 years old, and more than 55 years old. In Table 1, each country’s name is given using the three-digit International Olympic Committee (IOC) code. Additionally, general demographics, such as the number of adults aged 18 or older in the household, the number of children under the age of 18, and the highest level of education, were gathered (Table 2, Table 3 and Table 4).

### 2.3. Questionnaire Design

Participants were asked about their willingness to consume any of 12 specific insect powders (ant, ant egg, bee, beetle, caterpillar, cockroach, cricket, fly, fly larva, grasshopper, mealworm, and wasp) when they were used as an ingredient in five food types: a muffin or bread, cake, cracker, salty snack food, or meat patty. The question asked in the survey was “Please check each insect powder that you would be willing to try as part of a: (specified food)”. The insect powders were selected based on (a) literature searches for the types of insects eaten in various countries, (b) literature searches for the potential use of various insects as insect powders for human foods, and (c) discussions with consumers from multiple countries about foods that they believed it could contain insect powder. Ghosh et al. [38] noted that various species of insects may be more acceptable depending on the culture. The survey was conducted using a Check-All-That-Apply (CATA) method for each food type. CATA is a structured question format in which respondents are presented with a list of terms and asked to select all that apply to the focal sample [39]. Its main advantage is that the consumer perceives the task as easy and not tedious to complete [40,41]. This survey was conducted using Qualtrics (Provo, UT, USA), an online survey company, using databases maintained by Qualtrics or their partners. These databases typically contain more than one million consumers from each country.

### 2.4. Questionnaire Translation

The questionnaire (including demographic and other questions) was developed in English and subsequently translated into Portuguese, simplified Mandarin, Hindi, Japanese, Spanish, Russian, Afrikaans, and Thai for use in the appropriate countries. The English questionnaire was used for Australian, British, and US respondents. It also was offered as an option in India (along with Hindi) and South Africa (along with Afrikaans). The questionnaire translation process used a modification of the translation, review, adjudication, retesting, and documentation (TRAPD) approach, as used by Seninde and Chambers [42] based on Curtarelli and van Houten [43] and Harkness [44]. In detail, the English questionnaire was translated by a professional in the subject area who was a native speaker of the translated language and who also was fluent in English. It was then translated backward to English by a professional native speaker from another subject area. Then, the two professionals worked jointly (either in person or via online meeting) to check the final translation and confirm that the meanings were as intended. If disagreements occurred, a third party could have been consulted to adjudicate; however, the two professionals were able to reach an agreement in the few cases where the translations did not agree.

This procedure has been used for other questionnaires across several languages [28,45,46]. After the translation, a “soft launch” in each country with 50 respondents was conducted to examine each translated questionnaire and determine whether the questionnaires could be successfully comprehended and completed in the allocated time. Data from these respondents were tracked; no missing data were found, all data were found to be reasonable, and the data from the validity check questions were satisfactory—these were included to determine whether the consumers were focused. At this point, the questionnaires were approved and used in the test.

### 2.5. Data Analysis

Cochran’s Q test was used to determine the selection frequency of the CATA items and to see if there were significant differences between the countries and insect powder species. When significant differences existed, the critical difference (Sheskin) procedure was followed as a post hoc pairwise comparison analysis. In addition, a cluster analysis was conducted for each food type to determine which countries were similar in their responses to the specific insect powders being used in the products. To investigate whether a significant difference existed in the frequency of willingness to eat insect powder-containing foods for each country according to the three age groups (18–34, 35–54, and 55+), the Mann–Whitney U test was performed. The remaining four demographics were analyzed by dividing them into two groups as follows: (a) gender (male and female), (b) the highest level of education earned (high school or less and college or university graduate), (c) the number of adults in the participants’ households (1–2 and more than 3), (d) the number of children in the participants’ households (none or any). The Kruskal–Wallis test was used to investigate whether there was a significant difference in the frequency of willingness to eat insect powder-containing foods and each of these four demographics within each country. Correspondence analysis (CA) is a method performed to visualize correlations between samples and attributes using non-parametric data [47]. Thus, we performed a correspondence analysis to see if the clusters identified in the cluster analysis appeared the same in the CA results (graph not presented in this paper). The cluster analysis and correspondence analysis were conducted using SAS 9.4 (SAS Institute Inc., Cary, NC, USA), and the Cochran’s Q test, Kruskal–Wallis test, and Mann–Whitney U test were conducted using the XLSTAT software, version 2023.3.1 (New York, NY, USA). Microsoft Excel^TM^ (Santa Rosa, CA, USA) was used for simple calculations.

## 3. Results

### 3.1. Cluster Analysis

For muffins/bread, crackers, and cake, the willingness to eat certain insect powders was the same across the country groups. The countries were clustered into (A) the USA; (B) Australia, Japan, Russia, Spain, and the UK; (C) China, Mexico, Peru, and Thailand; and (D) Brazil, India, and South Africa. Salty snack foods and meat patties showed the same country groupings, except that China moved from Cluster C to Cluster X, where (W) USA; (X) Australia, China, Japan, Russia, Spain, and UK; (Y) Mexico, Peru, and Thailand; and (Z) Brazil, India, and South Africa (Table 5). The United States was clustered alone because it showed significantly higher willingness to consume (although it still showed reasonably low willingness) than the other 12 countries. A study by Woolf et al. [48] and Peksever, Ruby, and El [49] found that the more knowledge that US participants had about insect eating and the higher their educational level, the more willing they were to consume foods containing insects. In addition, many insect powder-containing foods are currently sold in the US, so this high exposure may have increased their familiarity. Clusters C and Y were clusters of countries with a higher frequency of selection of bee powder. In the cases of muffins/bread, crackers, and cake types, bee powder was selected relatively more frequently than the other insect-based powders, at about 10% or more, in all four countries (China, Mexico, Peru, and Thailand). However, in terms of salty snack foods and meat patties, the clusters were formed diffferently, because China showed lower willingness to consume bee powder (~7%) than the other three countries in Cluster Y. Due to this difference in the frequency values, only China was grouped differently. Clusters D and Z consisted of developing countries with a higher frequency of willingness to eat cricket powder, forming clusters together, and Clusters B and X were composed of countries with no noticeably higher values.

### 3.2. Consumers’ Willingness to Consume Insect Powder in Familiar Food Types

#### 3.2.1. Frequency of Insect Ingredient Selection by Country, Organized into Clusters

It was confirmed through Cochran’s Q test that there was a significant difference between the countries and insect powders by food type (*p*-value < 0.0001).

However, regardless of the food type, this study found that, in the USA (Cluster A), all insect species showed a higher frequency of selection than among the consumers in the remaining 12 countries (Table 5). There are several areas of the world, including Africa, Asia, and Latin America, where the practice of consuming insects as food is common [50]. Nevertheless, among the 13 countries, USA consumers showed the highest willingness to consume insect powders as ingredients.

This may be attributed to their familiarity with insect powders as food ingredients, rather than as intact whole insects. This research asked about the use of insect powders as ingredients for various foods. It is possible that the use of these processed ingredients did not align with the types of food that we studied, which may have confused the consumers in some countries. Although consumers in some countries are more likely to eat intact “insects” than consumers in the US, insect powders are not common ingredients in most of these countries. There are several insect-containing products (cricket powder protein bars, cookies, and chips, etc.) already sold in the US market, and the topic of insect protein has been discussed frequently in the food press in recent years. It is likely that consumers in other countries are also less concerned with increasing their protein content or less aware of insect protein overall.

Additionally, various studies [28,51] have shown that between 30 and 70 percent of US consumers are willing to try insect-containing ingredients, with higher percentages seen only after education about the benefits of using insect-based ingredients. In this study, respondents from the US also showed a higher percentage of willingness to eat insect powder-containing foods than respondents from other countries (Table 5). However, for the five food types and 12 insect types, the willingness to eat foods containing specific insect powders among USA respondents was only up to 25.4%, indicating that these consumers were still less willing to eat insect powder-containing foods.

Cockroach, fly, and fly larva powders were selected at about 1% or less for all five foods types in all 12 countries, except the US. Other than the US, the willingness to consume cricket powder was the highest in Clusters D and Z, which included Brazil, India, and South Africa. After the US, bee powder was chosen the most in Clusters C (which included China, Mexico, Peru, and Thailand) and Y (which included Mexico, Peru, and Thailand). Grasshopper powder was the next most selected in these clusters.

Of the 12 insects, the top three insect powders most frequently selected in terms of willingness to eat were bee, cricket, and grasshopper powders, and the bottom three were cockroach, fly, and fly larva powder. Among them, the intention to consume bee powder was the highest in 11 countries, with the exception of India and South Africa. Similarly, Rovai et al. [52] found that crickets were the most acceptable insect type, followed by grasshoppers.

In comparison, wasp powder, in the same insect order as bees (Hymenoptera), was selected mostly at a low frequency. However, it was one of the top three insect powders that Indian consumers were willing to consume (muffins/bread, 3%), and it was also one of the top three insect powders in South Africa (salty snack foods, 2.5%; crackers, 2.9%).

All 13 countries showed the highest willingness to eat muffins/bread containing insect powder among the five food types. This seems to be due to the fact that these foods are traditionally widely consumed as staple foods or snacks around the world. On the other hand, among the five types, cake showed the lowest frequency value, possibly because it is not a type of food in which protein content is important. Previous studies [34] have also observed the negative effects of adding insect powders when foods did not fulfill consumers’ wishes, such as mealworm muffins and chocolate grasshoppers. In these cases, the consumers perceived the foods as rotten.

In some Japanese regions, wasp larvae are eaten as a crispy snack, sometimes mixed with cooked imago wasp [53]. In some Southeast Asian countries, insects have been part of the traditional cuisine for centuries; in Thailand, grasshoppers and crickets are commonly eaten [54]. Insects that eat plant tissue, such as grasshoppers and beetles, are considered more hygienic than those that eat animal carcasses and live in unhygienic conditions [55]. Because of these practices, there seems to be higher willingness to consume these insect powders.

Fly, cockroach, and insect larvae are considered unappealing because of their contact with decaying human and animal cadavers or human feces [56]. Cockroaches are considered urban household pests and can cause potential health problems for individuals [57], and the smell of decay and contact with carcasses associated with flies and insect larvae elicit high levels of disgust [56]. For these reasons, these insect powders elicit negative perceptions; furthermore, the willingness to consume them seems to be very low.

#### 3.2.2. Frequency of Insect Powder Selection by Demographic

The US respondents exhibited the most significant gender-specific differences in almost all insect powders across all five food types; males were significantly more willing to consume various foods containing some insect powders (Table S1). However, for some countries, there were no significant differences in one or more food types. For example, Australia showed no significant differences in insect powders from various species for any food types. In China, female respondents were more willing to consume insect-containing foods and showed significant differences for ant powder in muffins/bread, grasshopper powder in crackers, ant egg powder and ant powder in salty snack foods, and ant egg powder in meat patties. Moreover, there were a few more exceptions where female respondents were more willing to consume: bee power in muffins/bread (Russia) and salty snack foods (Thailand) and wasp powder in cakes (Mexico).

Differences in the willingness to consume insect powders across the five food types by age were mostly found in the US, Peru, Thailand, South Africa, and UK respondents (Table S2). For Brazil and Spain, there were no significant age differences in all insect powders across the five food types; this was followed by Australia, China, and Russia, showing no significant differences in four food types, and India, Japan, and Mexico, showing no significant differences in three food types. When significant differences existed according to the age group, the younger age group was generally more willing to consume insect-containing foods in Australia, India, Japan, Mexico, Russia, the UK, and the US. In contrast, older age groups were more willing to consume insect-containing foods in Japan, Peru, South Africa, and Thailand.

Participants with an education level of high school or lower often showed a significantly higher frequency of willingness to eat insect powder-containing foods (Table S3). There was a significant difference in Japan and Mexico when muffins/bread contained beetle powder and when fly larva powder was considered in India for the same food type. For crackers, Japan (bee powder), China (caterpillar and fly powders), Mexico (caterpillar and wasp powders), and Thailand (cockroach powder) showed similar results. Meanwhile, respondents with a high school education or lower showed significantly higher willingness to consume certain foods: those in China (fly powder), Mexico (caterpillar and wasp powders), Thailand (fly powder), and India (fly larva powder) were more likely to consume cakes, and those in China (wasp powder), Japan (ant powder), and Mexico (bee and caterpillar powders) were more likely to consume salty snacks. However, a few insect powders were selected more frequently by college graduates or those with higher education levels in Australia (ant egg powder in crackers, meat patties, and salty snacks), South Africa (cricket powder in crackers and salty snacks), Brazil (wasp powder in cakes and cricket powder in salty snacks), and the UK (bee powder in cakes). 

When the number of adults in the participant’s household was compared (one or two versus three or more), the respondents in some countries showed similar tendencies in terms of their willingness to consume insect powder-containing foods (Table S4). Respondents in Brazil (ant egg powder in crackers, beetle powder in cakes and meat patties), Mexico (bee powder in crackers, cake, and meat patties), Peru (cockroach powder in cakes), and the UK (beetle powder in meat patties), with one to two adults in their households, showed significantly higher willingness to consume than those with more adults in the household. On the other hand, respondents with three or more adults in their households showed significantly higher willingness to eat such foods in Australia (ant egg and caterpillar powders in salty snack foods; caterpillar powder in meat patties) and Thailand (mealworm powder in muffins/bread and ant egg and bee powders in meat patties). In the US and Japan, the results were mixed. For mealworm powder in crackers and salty snack foods and fly larva powder in cakes, US respondents with one to two adults in their households showed significantly higher willingness to consume, whereas wasp powder in salty snack foods was selected more often by respondents with more than three adults in their households. In Japan, cricket powder in meat patties was selected more often by respondents with one to two adults in their households, whereas bee powder in crackers and beetle powder in cakes were selected more by respondents with three or more adults in their households.

The number of children in the participant’s household showed the most significant differences when comparing those with no children and one or more children. In most countries, the respondents were more willing to eat insect powder-containing food types when they had children in the household. However, in Peru and South Africa, respondents with no children in the household showed greater willingness to consume insect powder-containing foods (Table S5).

## 4. Discussion

### 4.1. Factors Related to Consumers’ Perceptions of Insect Consumption

This study advances our understanding of the willingness to eat insect powders when contained in familiar food types. USA respondents were more willing to eat insect-based powdered ingredients than those in other countries across all 12 insect species, presumably due to the influence of many media reports and studies on insect powder use. “None of the above”, i.e., not willing to eat any of the insect powders, was the most commonly selected response among all food types evaluated, indicating that they generally were not willing to eat insects, regardless of the type.

Castro and Chambers [30] reported that one of the most significant reasons that consumers did not wish to try insect-based products is their appearance. Consumers did not wish to see insect pieces in their food. Moreover, Meyer-Rochow and Hakko [58] mentioned similar results regarding the visual appearance. They suggested that using a powder or paste, where the insects were not noticed, is a better way to introduce insect use in food. Based on these studies, we expected that consumers’ willingness to consume insects would be higher when they were included in food as a powder. However, it was still low, at less than 35% for all insects in all 13 countries (refer to Table 5). These results show that considerable exposure and education will be needed to help consumers to understand the environmental and nutritional benefits of consuming insect powders as ingredients in various food types.

Besides the appearance of the insects themselves, the main reason that many people are reluctant to eat insect-containing foods is “disgust”. In a prior study [30], more than 50% of respondents in all countries except Mexico and Thailand stated that they would not consume insect-based products because they were disgusting. Among them, Japan highlighted this reason the most, with 77% of respondents; this in turn agreed with the perception that it was disgusting among 68% in the US and 67% in Spain. Similarly, this study found that, regardless of the five food types mentioned, none of the insect powders were seen as a desirable addition. Several studies have shown that conventional insect eaters are less disgusted than non-insect eaters [59,60], but the presence or absence of insect consumption experience was not considered in our study.

Aboriginal populations in Australia consumed termites and maggots long before the British settled there [61], and, while many maintained this tradition, the consumption of insects became obscure due to the complexity of modern cultures [62]. The insect-eating tradition in China has been around for more than 3000 years [63,64], but, now, the knowledge and tradition of insect consumption has disappeared in some areas, and they are only consumed regularly in some parts of China, such as in the minority areas of Yunnan Province [63]. Another Asian country, Japan, has a long tradition of consuming edible insects [65], but, in Asian countries, insect consumption is declining due to the westernization of diets [66,67], and Japan is no exception. Stone et al. [68] surveyed British consumers, with a large percentage of participants agreeing that the consumption of edible insects was mostly neglected due to the import of other foods, and only a few areas still follow the tradition of consuming edible insects.

As such, despite the fact that some countries traditionally consume insects in some regions, the low willingness to consume observed in this study shows decreased interest in insects as food or food ingredients as the consumption of western-style diets increases [69].

The lack of interest in eating products containing insect powders also may be because the acceptance of whole insects as food in certain countries, such as Mexico and Thailand, does not mean that consumers also are willing to eat insect powders when they are added to other foods. Insects as food may be traditional in some countries, but ground insects or insect powders are a relatively new ingredient that has not been adopted in many countries. In fact, approval for the use of some insect powders in food has only recently occurred in Europe [70,71,72].

### 4.2. Willingness to Consume by Insect Powder Type and Food Category

Barton, Richardson, and McSweeney [25] first conducted a survey on attitudes toward insect consumption; then, after the participants had consumed a drink produced from cricket protein powder, they completed the same questionnaire again. The respondents’ aversion to insect consumption was significantly reduced. The willingness to use cricket powder in daily life increased, and the willingness to recommend it to friends also became stronger. Moreover, in most studies that have evaluated both pre- and post-tasting experiences, the overall liking scores for insect-based products improved after tasting [73,74,75,76,77,78]. However, in this study, the respondents’ beliefs, perceptions, and cultural biases were reflected in the data, which were based only on survey questions and not on the consumption of foods containing insect powders. These data, and those of previous authors, suggest that if companies wish to include insect powders in various types of products, it will be necessary for consumers to have the opportunity to try (i.e., sample or taste) the products before purchase.

Moreover, this study identified several food types and insect species that might lower the initial barrier for consumer acceptance. When muffins or bread contained insect powder, consumers were most willing to eat them, and participants from all 13 countries tended to prefer bee, cricket, and grasshopper powders among the 12 insects studied. In both the USA and Europe, some species of cricket and mealworms have received approval, but other insects may be able to be used under novel food regulations with good manufacturing practices. In contrast, the willingness to consume was the lowest when cake, a sweet, dessert-style product, contained insect powder. Contrary to the findings of this study, Megido et al. [14] found that consumers were more willing to consume insect-based ingredients in snacks (37%) and desserts (23%). However, Lombardi et al. [71] also emphasized that enriching pasta with insect powder has been shown to be more appropriate than chocolate bars containing insects. In addition, cockroaches, flies, and fly larvae were associated with a marked unwillingness to consume. This may be because these insects are associated with unhygienic environments compared to other insects.

Some authors [79] have stated that, in Europe, people prefer to consume processed forms of insects, such as in flour or powders, while, in Asia, insects are primarily consumed in their unprocessed forms (Japan, China, and ASEAN countries such as Thailand, Malaysia, Indonesia, Singapore, the Philippines, Brunei, Vietnam, Cambodia, and Myanmar)—for example, certain species of crickets and palm weevils are consumed cooked as whole insects. As such, since each country has different preferred forms of insects, it is important to consider the consumption behavior by country, rather than applying the same food type, insect type, and form to all countries. In addition, cultural factors also play a major role in consumers’ willingness to consume them. Exposure to a certain insect results in common preferences and fundamental expectations within different cultures, so the preferred insect species also differ, because each culture traditionally consumes different species [34].

### 4.3. Demographic Differences

Many male consumers state that they like to try new foods [27], and they were more willing to try insect-containing foods in this study and in other studies [28,35,36,48]. However, we also found that females in China were more willing to consume foods containing insect powders than were males, which is similar to a prior study [28]. Similarly to the current study, where ‘cake’ was the food product, female participants in Mexico were more willing to try a sample containing insect powder [35], although the food type was a different sweet baked product, namely a cookie (refer to Table 1 and Supplementary Table S1).

Regarding age differences, Megido et al. [14] reported that 93.8% of the 19–25 age group had knowledge of insects as food, and Castro and Chambers [28] showed slightly higher willingness to eat insect-containing foods among consumers in the 18–34 age group compared to 35–54 year olds. In other studies, younger age groups also were more likely to eat insects [23,27]. Our data confirm these findings. More younger respondents were willing to eat insect powders in certain foods than older consumers (refer to Table 1 and Supplementary Table S2).

Studies examining the educational level as a source of variation in the willingness to eat insect-containing foods generally have shown no effect of this variable [80,81]. Our results are contrary to these findings as we observed that those with a lower level of education (i.e., high school or less) often were more willing to consume insect powders in various products (refer to Table 2 and Supplementary Table S3).

We found no studies that compared the number of adults or children in the household to determine the interest in consuming insects or insect-containing foods. In our study, the results were mixed when comparing two or fewer to three or more adults in the household. Thus, no obvious conclusions can be made (refer to Table 3 and Supplementary Table S4). Regarding children, we found that having children in the household generally increased the willingness to consume various products containing some insect powders (refer to Table 4 and Supplementary Table S5). Because we did not study the reasons for the willingness or not to eat foods containing insect powders, we can only speculate on why this result was noted. It could be because (a) parents were interested in the potential nutritional improvements in products containing insect powders; (b) younger adults may be more likely to have children in the household, and younger people were more willing to try foods containing insect powders; (c) households with children may consider insect powders as a way to save money (generally, this is not the case at present); (d) parents may believe that their children would be more willing to try unusual foods or other potential factors may be important. Further work on the willingness to consume insect powders and the importance of children in the household is needed.

### 4.4. Contributions to the Future

In previous studies, early adopters of edible insects were identified and classified based on familiarity and dietary habits [52], and cross-sectional studies were conducted on the intent to consume insects [49]. The difference is that, in this study, we selected five familiar food types and examined consumers’ willingness to consume insect powders from various insect types in these foods. Insect powders were specifically studied in this research because Castro and Chambers [30] noted that a high percentage of people preferred the insects to be invisible, with no visual or textural indications that insects were present. In another study, when cricket powder was directly used as an ingredient in chocolate chip cookies, the acceptability was the highest when the cookies contained 15 percent powder [35]. Based on these prior studies, we selected five familiar food types and examined consumers’ willingness to consume insect powders from various insect types in these foods, thus contributing to broader knowledge of the insect powder market.

Based on these findings, when developing and introducing insect-containing foods, the food types and insect species with a more positive influence on consumers’ perceptions and consumption should be selected. The focus should be on improving the marketing strategies to make insect-based products more attractive [82]. In addition, as shown with other “new ingredients” [83], it is critical that the food options chosen to include insect powders be appropriate for the culture and appropriate information be given. The adoption of insect powders could make a potential contribution to food security [84]. As the awareness of their nutritional and environmental benefits increase, the global demand for insect powders could increase, providing opportunities for insect powder production and more food industry collaborations to develop [38]. Due to their lower environmental impacts compared to traditional livestock, insects are often advocated for as a sustainable protein source [11,12,85].

## 5. Limitations

There are several limitations to this study. First, because insect-containing foods were not directly consumed and we only assessed the willingness to consume them using an online survey, our findings may not directly reflect consumers’ willingness to consume such products after being given educational information or tasting specific products. The degree of food neophobia also influences consumers’ willingness to eat various foods. Thus, using a questionnaire such as the Food Neophobia Scale (FNS) developed by Pliner and Hobden [86] would help to determine the correlation among the intention to eat insects and the degree of food neophobia.

We also believe that the data from this study make it clear that consumers require knowledge of the ingredient/product. For example, in this study, where a novel ingredient was tested, perceptions about the texture, appearance, and safety were likely exhibited by the consumers, but this may not be the reality. For example, informing consumers upfront that the novel ingredient is safe for human consumption and will not change the texture or appearance of the food may be critical for insect powders, where safety and sensory issues have been noted as potential problems among consumers. Conducting research on consumers’ intentions to consume, with the actual tasting of products, could further increase our understanding of insect-based foods

Lastly, we were able to balance demographics such as gender and age across countries. However, there were considerably larger numbers of consumers with college/university degrees, with more than two adults in the household, and with no children in the household in some countries than in others. These types of differences are unavoidable because some countries have households that include a larger proportion of extended relatives or larger families or may have more opportunities for education. However, these data also reflect the difficulty of obtaining a truly random cross-section of consumers using internet-based surveys. Although countries such as Thailand and India have extensive cell and internet usage, our data suggest that those who complete surveys online have a higher level of education and perhaps a different household structure (i.e., fewer children in the household) compared to those who do not complete such surveys. This must be considered when decisions are made based on such survey results. It is worth mentioning that, in developed countries, technology is more broadly accessible to individuals with lower educational levels and incomes than in other countries. Thus, the demographics in less developed countries may be more skewed towards more highly educated respondents, as seen in this survey.

## 6. Conclusions

Although people’s interest in insect consumption as an alternative to meat proteins is increasing worldwide, insect powder-based products are rarely consumed. To close this gap, this study performed a multi-country comparison of consumers to understand the perceptions of insect powder consumption. This research evaluated the use of insect-based powders in familiar food types. We believed that the use of insect powder as a potential ingredient would overcome the texture or appearance issues associated with insect parts. This phenomenon did not occur in the case of the rejection of insect powder as an ingredient, regardless of the food type. Clearly, consumers require education or actual experience with products produced from insect powder before any of the insects studied can be successfully included in products for mass consumption.

Few respondents reacted positively to any of the 12 types of insect powders in this study. Among the insect powders, cockroach, fly, and fly larva powders showed the lowest willingness to consume in most countries, while powders from bees, crickets, and grasshoppers were more frequently selected. Among the five food types, muffins/bread had the highest percentages of respondents willing to eat them if they contained certain types of insect powders, while cake had the lowest percentages of willingness. Based on these results, muffins or bread should be among the first types of foods developed with bee, cricket, or grasshopper insect powders. This may encourage some consumers to try insect powder-based products and discover whether they meet their sensory and health needs.

Efforts should be made to lower the barriers through education or sampling and trials of insect powder-based food consumption. Legendre et al. [87] concluded that reliable media information helped to motivate consumers to participate in purchasing activities. In addition, Gomez-Corona and Valentin [88] found that stimulating consumers’ gastronomic curiosity could increase their willingness to try different insect species, rather than simply informing them that insects should be eaten because they are healthy or benefit the planet. This could change consumers’ perceptions of insects, moving from concerns about their texture and safety to the potential benefits. This survey encompassed respondents from 13 countries, but the results will benefit not only the food industry but also the marketing sector and insect producers.

However, for insect-based products to be used as sustainable alternatives, insect-related regulations will need to be established in each country, and support for production and processing technologies will also be needed.

## Figures and Tables

**Table 1:** Demographic profile of the participants from 13 countries (n = 8191).

	AUS ^1^	BRA	CHN	IND	JPN	MEX	PER	RUS	RSA	ESP	THA	GBR	USA
Total	630	630	630	631	630	630	630	630	630	630	630	630	630
Male	315	315	315	315	315	315	315	315	315	315	315	314	314
18–34	105	105	105	105	105	105	105	105	105	105	105	104	104
35–54	105	105	105	105	105	105	105	105	105	105	105	105	105
55+	105	105	105	105	105	105	105	105	105	105	105	105	105
Female	315	315	315	316	315	315	315	315	315	315	315	316	316
18–34	105	105	105	106	105	105	105	105	105	105	105	106	105
35–54	105	105	105	105	105	105	105	105	105	105	105	105	106
55+	105	105	105	105	105	105	105	105	105	105	105	105	105

^1^ IOC code: AUS (Australia), BRA (Brazil), CHN (China), IND (India), JPN (Japan), MEX (Mexico), PER (Peru), RUS (Russia), ESP (South Africa), THA (Thailand), GBR (United Kingdom), USA (United States of America).

**Table 2:** Participants’ levels of education.

	Primary School or Less n * (%)	High School n (%)	College or University Graduate n (%)
Australia	23 (3.7)	228 (36.2)	379 (60.1)
Brazil	5 (0.8)	254 (40.3)	371 (58.9)
China	4 (0.6)	130 (20.6)	496 (78.8)
India	2 (0.3)	28 (4.4)	601 (95.3)
Japan	13 (2.1)	209 (33.1)	408 (64.8)
Mexico	2 (0.3)	26 (4.1)	602 (95.6)
Peru	2 (0.3)	59 (9.4)	569 (90.3)
Russia	5 (0.8)	51 (8.1)	574 (91.1)
South Africa	2 (0.3)	229 (36.4)	399 (63.3)
Spain	29 (4.6)	267 (42.4)	334 (53.0)
Thailand	11 (1.7)	79 (12.6)	540 (85.7)
UK	81 (12.9)	164 (26.0)	385 (61.1)
USA	8 (1.3)	286 (45.4)	336 (53.3)

* “n” denotes the number of participants.

**Table 3:** Number of adults aged 18 or older in participants’ households.

	1 n * (%)	2 n (%)	3 n (%)	4 n (%)	5 n (%)	6 n (%)
Australia	132 (21.0)	346 (54.9)	92 (14.6)	39 (6.2)	21 (3.3)	0 (0.0)
Brazil	69 (11.0)	259 (41.1)	164 (26.0)	101 (16.0)	37 (5.9)	0 (0.0)
China	14 (2.2)	129 (20.5)	326 (51.7)	79 (12.6)	82 (13.0)	0 (0.0)
India	15 (2.4)	158 (25.0)	131 (20.8)	191 (30.3)	136 (21.5)	0 (0.0)
Japan	109 (17.3)	225 (35.7)	156 (24.8)	98 (15.5)	42 (6.7)	0 (0.0)
Mexico	3 (0.5)	36 (5.7)	253 (40.1)	139 (22.1)	111 (17.6)	88 (14.0)
Peru	5 (0.8)	21 (3.3)	185 (29.4)	140 (22.2)	149 (23.7)	130 (20.6)
Russia	74 (11.7)	327 (52.0)	138 (21.9)	63 (10.0)	28 (4.4)	0 (0.0)
South Africa	67 (10.6)	298 (47.3)	132 (21.0)	75 (11.9)	58 (9.2)	0 (0.0)
Spain	1 (0.2)	62 (9.8)	318 (50.5)	145 (23.0)	87 (13.8)	17 (2.7)
Thailand	72 (11.4)	119 (18.9)	141 (22.4)	177 (28.1)	121 (19.2)	0 (0.0)
UK	123 (19.5)	352 (55.9)	95 (15.1)	43 (6.8)	17 (2.7)	0 (0.0)
USA	167 (26.5)	302 (47.9)	94 (14.9)	47 (7.5)	20 (3.2)	0 (0.0)
Total	851	2634	2225	1337	909	235

* “n” denotes the number of participants.

**Table 4:** Number of children under 18 (<18) in participants’ households.

	None n * (%)	1 n (%)	2 n (%)	3 n (%)	4 or More n (%)
Australia	419 (66.5)	109 (17.3)	75 (11.9)	23 (3.7)	4 (0.6)
Brazil	349 (55.4)	166 (26.3)	81 (12.9)	21 (3.3)	13 (2.1)
China	268 (42.5)	317 (50.3)	43 (6.8)	1 (0.2)	1 (0.2)
India	255 (40.4)	227 (36.0)	117 (18.5)	22 (3.5)	10 (1.6)
Japan	473 (75.2)	106 (16.8)	40 (6.3)	9 (1.4)	2 (0.3)
Mexico	238 (37.8)	179 (28.4)	161 (25.6)	38 (6.0)	14 (2.2)
Peru	248 (39.4)	170 (27.0)	145 (23.0)	52 (8.2)	15 (2.4)
Russia	326 (51.7)	169 (26.8)	110 (17.5)	22 (3.5)	3 (0.5)
South Africa	339 (53.8)	126 (20.0)	112 (17.8)	42 (6.7)	11 (1.7)
Spain	368 (58.4)	163 (25.9)	85 (13.5)	14 (2.2)	0 (0.0)
Thailand	301 (47.8)	190 (30.1)	107 (17.0)	17 (2.7)	15 (2.4)
UK	401 (63.7)	106 (16.8)	88 (14.0)	26 (4.1)	9 (1.4)
USA	424 (67.3)	103 (16.4)	65 (10.3)	27 (4.3)	11 (1.7)
Total	4409	2131	1229	314	108

* “n” denotes the number of participants.

**Table 5:** CATA task results regarding frequency (%) of consumers’ willingness to consume specific insect powders in five food types, arranged by country cluster ^(1),(2)^.

Cluster ^(3)^	Country	Ant Egg	Ant	Bee	Beetle	Caterpillar	Cockroach	Cricket	Fly Larva	Fly	Grasshopper	Mealworm	Wasp
		**Muffin or Bread**											
A	USA	**10.3 ^a^** ^(4)^	**17.0 ^a^**	**25.4 ^a^**	**15.9 ^a^**	**12.7 ^a^**	**6.2 ^a^**	25.4 ^a^	**7.1 ^a^**	**7.3 ^a^**	**22.7 ^a^**	**11.7 ^a^**	**10.8 ^a^**
B	Australia	1.1 ^d^	2.4 ^d^	7.6 ^c^	3.0 ^b^	2.1 ^c^	0.3 ^cd^	7.3 ^c^	0.2 ^b^	0.5 ^c^	5.6 ^d^	2.4 ^bcd^	**1.1 ^bc^**
	Japan	**1.0 ^d^**	1.3 ^d^	7.1 ^c^	1.7 ^b^	1.7 ^c^	0.6 ^cd^	**2.5 ^d^**	0.8 ^b^	0.3 ^c^	6.0 ^cd^	0.6 ^d^	1.4 ^bc^
	Russia	**1.0 ^d^**	2.9 ^d^	6.7 ^c^	1.1 ^b^	0.8 ^c^	**0.0 ^d^**	3.5 ^cd^	**0.0 ^b^**	0.3 ^c^	6.0 ^cd^	0.5 ^d^	1.4 ^bc^
	Spain	1.3 ^cd^	3.5 ^d^	7.1 ^c^	1.3 ^b^	0.8 ^c^	0.3 ^cd^	3.5 ^cd^	**0.0 ^b^**	0.2 ^c^	3.3 ^d^	1.0 ^cd^	3.2 ^bc^
	UK	1.4 ^cd^	3.2 ^d^	6.5 ^c^	2.4 ^b^	1.3 ^c^	0.3 ^cd^	5.7 ^cd^	0.8 ^b^	0.8 ^c^	4.4 ^d^	1.9 ^bcd^	1.9 ^bc^
C	China	1.3 ^cd^	3.7 ^cd^	13.0 ^b^	**1.0 ^b^**	1.3 ^c^	0.6 ^cd^	3.5 ^cd^	0.3 ^b^	0.5 ^c^	6.5 ^cd^	2.7 ^bcd^	1.7 ^bc^
	Mexico	3.8 ^bc^	7.8 ^b^	16.0 ^b^	1.7 ^b^	2.4 ^bc^	0.2 ^d^	14.1 ^b^	**0.0 ^b^**	**0.0 ^c^**	11.7 ^b^	3.5 ^bc^	3.5 ^b^
	Peru	1.9 ^cd^	7.1 ^bc^	16.8 ^b^	1.7 ^b^	2.9 ^bc^	0.2 ^d^	7.3 ^c^	**0.0 ^b^**	**0.0 ^c^**	6.2 ^cd^	3.7 ^b^	1.9 ^bc^
	Thailand	4.9 ^b^	3.2 ^d^	14.0 ^b^	2.2 ^b^	2.5 ^bc^	**0.0 ^d^**	12.7 ^b^	0.2 ^b^	**0.0 ^c^**	10.3 ^bc^	1.4 ^bcd^	2.1 ^bd^
D	Brazil	4.9 ^b^	3.5 ^d^	5.2 ^cde^	2.2 ^b^	0.6 ^bc^	0.3 ^b^	**34.1 ^a^**	0.3 ^b^	0.2 ^b^	4.6 ^de^	1.3 ^bc^	3.2 ^b^
	India	3.5 ^bc^	**0.5 ^d^**	**2.2 ^e^**	2.5 ^b^	**0.0 ^c^**	0.6 ^b^	10.6 ^cde^	1.1 ^b^	1.1 ^b^	**1.9 ^e^**	**0.5 ^c^**	3.0 ^b^
	South Africa	2.9 ^bc^	1.3 ^d^	2.4 ^de^	3.2 ^b^	1.4 ^bc^	0.2 ^b^	21.3 ^b^	0.3 ^b^	0.3 ^b^	3.5 ^de^	1.3 ^bc^	2.9 ^b^
		**Cracker**											
A	USA	**9.4 ^a^**	**14.3 ^a^**	**20.6 ^a^**	**13.5 ^a^**	**12.7 ^a^**	**4.9 ^a^**	**24.8 ^a^**	**6.3 ^a^**	**8.7 ^a^**	**20.8 ^a^**	**11.9 ^a^**	**11.4 ^a^**
B	Australia	1.0 ^c^	1.7 ^cde^	4.8 ^cd^	1.9 ^b^	1.4 ^c^	0.2 ^cd^	5.2 ^cde^	**0.0 ^b^**	0.3 ^c^	4.0 ^de^	1.6 ^bc^	1.9 ^bc^
	Japan	1.3 ^c^	**1.0 ^e^**	5.2 ^cd^	**0.5 ^b^**	1.0 ^c^	**0.0 ^d^**	**2.9 ^e^**	0.2 ^b^	**0.0 ^c^**	5.4 ^cd^	0.6 ^c^	1.3 ^bc^
	Russia	**0.3 ^c^**	1.9 ^cde^	4.0 ^cd^	1.3 ^b^	0.6 ^c^	**0.0 ^d^**	3.5 ^de^	**0.0 ^b^**	0.2 ^c^	3.7 ^de^	**0.3 ^c^**	**1.0 ^bc^**
	Spain	1.0 ^c^	2.9 ^bcde^	4.8 ^cd^	1.6 ^b^	1.0 ^c^	0.2 ^cd^	4.1 ^de^	**0.0 ^b^**	**0.0 ^c^**	3.8 ^de^	1.1 ^c^	1.4 ^bc^
	UK	1.1 ^c^	1.7 ^cde^	4.4 ^cd^	2.1 ^b^	1.4 ^c^	0.3 ^cd^	4.3 ^de^	1.1 ^b^	0.8 ^bc^	2.9 ^de^	1.3 ^c^	1.4 ^bc^
C	China	1.1 ^c^	4.3 ^bcd^	7.8 ^bc^	1.0 ^b^	1.1 ^c^	**0.0 ^d^**	4.0 ^de^	0.2 ^b^	0.6 ^bc^	5.1 ^cde^	1.1 ^c^	1.7 ^bc^
	Mexico	2.7 ^bc^	4.9 ^bc^	10.5 ^b^	1.3 ^b^	1.6 ^c^	**0.0 ^d^**	11.7 ^b^	0.2 ^b^	**0.0 ^c^**	9.4 ^b^	2.5 ^bc^	3.3 ^b^
	Peru	1.1 ^c^	5.4 ^b^	11.7 ^b^	1.6 ^b^	3.2 ^bc^	**0.0 ^d^**	7.3 ^cd^	0.2 ^b^	**0.0 ^c^**	5.6 ^bcd^	3.8 ^b^	2.2 ^bc^
	Thailand	3.8 ^b^	2.4 ^bcde^	10.0 ^b^	2.1 ^b^	1.7 ^c^	0.2 ^cd^	9.0 ^bc^	0.2 ^b^	0.2 ^c^	7.9 ^bc^	1.4 ^bc^	1.3 ^bc^
D	Brazil	2.9 ^bc^	2.4 ^bcde^	4.8 ^cd^	3.2 ^b^	**0.2 ^c^**	0.3 ^b^	18.7 ^b^	0.2 ^b^	0.8 ^b^	3.5 ^de^	1.1 ^c^	2.2 ^b^
	India	2.2 ^bc^	1.6 ^de^	**1.9 ^d^**	1.3 ^b^	**0.2 ^c^**	0.3 ^b^	7.5 ^def^	0.2 ^b^	0.8 ^b^	**1.3 ^e^**	0.5 ^c^	1.4 ^b^
	South Africa	1.4 ^bc^	1.6 ^de^	2.1 ^d^	2.9 ^b^	1.0 ^bc^	0.3 ^b^	13.3 ^c^	0.3 ^b^	0.5 ^b^	3.5 ^de^	0.8 ^c^	2.9 ^b^
		**Cake**											
A	USA	**9.4 ^a^**	**13.3 ^a^**	**19.7 ^a^**	**13.5 ^a^**	**12.2 ^a^**	**4.4 ^a^**	**20.8 ^a^**	**5.9 ^a^**	**9.0 ^a^**	**18.1 ^a^**	**11.3 ^a^**	**10.2 ^a^**
B	Australia	1.3 ^c^	2.2 ^bc^	5.2 ^de^	1.6 ^b^	1.6 ^cd^	**0.0 ^c^**	4.0 ^bcd^	**0.0 ^b^**	0.6 ^bc^	4.0 ^bcd^	1.9 ^bc^	1.3 ^bc^
	Japan	**0.6 ^c^**	1.0 ^c^	4.4 ^ef^	0.6 ^b^	1.0 ^cd^	0.3 ^c^	**1.3 ^d^**	0.2 ^b^	0.2 ^c^	4.1 ^bcd^	0.6 ^c^	1.3 ^bc^
	Russia	**0.6 ^c^**	1.7 ^bc^	4.1 ^ef^	**0.5 ^b^**	**0.0 ^d^**	**0.0 ^c^**	1.7 ^d^	**0.0 ^b^**	0.2 ^c^	1.3 ^cd^	0.2 ^c^	1.0 ^bc^
	Spain	1.1 ^c^	2.2 ^bc^	4.6 ^e^	1.4 ^b^	1.0 ^cd^	0.2 ^c^	2.4 ^cd^	0.3 ^b^	0.2 ^c^	3.2 ^bcd^	1.0 ^bc^	1.3 ^bc^
	UK	0.8 ^c^	2.1 ^bc^	3.2 ^ef^	2.2 ^b^	1.1 ^cd^	0.3 ^c^	3.3 ^cd^	1.0 ^b^	1.0 ^bc^	2.9 ^bcd^	1.1 ^bc^	1.0 ^bc^
C	China	1.3 ^bc^	3.0 ^bc^	5.9 ^cde^	**0.5 ^c^**	1.1 ^bc^	0.2 ^b^	1.9 ^de^	**0.0 ^b^**	0.6 ^b^	4.6 ^bcd^	1.4 ^bc^	1.6 ^bc^
	Mexico	2.2 ^bc^	4.6 ^b^	9.7 ^bc^	1.3 ^b^	1.1 ^cd^	**0.0 ^c^**	7.3 ^b^	**0.0 ^b^**	0.2 ^c^	5.2 ^b^	2.5 ^bc^	2.5 ^b^
	Peru	1.7 ^bc^	4.6 ^b^	11.3 ^b^	1.4 ^b^	2.5 ^bcd^	0.2 ^c^	4.3 ^bcd^	**0.0 ^b^**	**0.0 ^c^**	4.8 ^bc^	3.2 ^b^	1.9 ^bc^
	Thailand	3.8 ^b^	1.6 ^bc^	8.9 ^bcd^	1.3 ^b^	2.1 ^cd^	0.2 ^c^	5.9 ^bc^	**0.0 ^b^**	0.2 ^c^	5.4 ^b^	1.4 ^bc^	1.1 ^bc^
D	Brazil	2.5 ^bc^	2.7 ^bc^	5.1 ^def^	3.3 ^b^	0.5 ^bc^	0.2 ^b^	17.9 ^a^	0.3 ^b^	**0.0 ^b^**	3.0 ^bcd^	1.1 ^bc^	3.2 ^b^
	India	2.1 ^b^	**0.6 ^c^**	**1.6 ^f^**	1.1 ^bc^	0.3 ^bc^	0.2 ^b^	3.3 ^cde^	0.2 ^b^	1.1 ^b^	**1.1 ^d^**	**0.0 ^c^**	**0.5 ^c^**
	South Africa	1.1 ^c^	1.3 ^c^	2.9 ^ef^	2.1 ^bc^	0.8 ^bc^	0.2 ^b^	7.9 ^b^	0.3 ^b^	0.5 ^b^	2.2 ^bcd^	0.8 ^bc^	2.1 ^bc^
		**Salty snack food**											
W	USA	**9.0 ^a^**	**16.7 ^a^**	**22.5 ^a^**	**14.8 ^a^**	**12.4 ^a^**	**4.6 ^a^**	**24.3 ^a^**	**7.0 ^a^**	**7.6 ^a^**	**21.6 ^a^**	**10.6 ^a^**	**11.4 ^a^**
X	Australia	1.1 ^c^	2.5 ^c^	5.4 ^def^	2.1 ^b^	1.1 ^c^	**0.0 ^b^**	6.0 ^cd^	**0.0 ^b^**	0.3 ^c^	4.6 ^de^	2.2 ^bc^	1.9 ^bc^
	China	1.3 ^c^	4.1 ^bc^	7.6 ^cd^	1.7 ^b^	0.8 ^c^	0.3 ^b^	3.0 ^cd^	0.2 ^b^	0.5 ^c^	6.8 ^cd^	1.1 ^bc^	1.3 ^bc^
	Japan	1.1 ^c^	1.7 ^c^	6.3 ^cde^	**0.3 ^b^**	1.4 ^c^	0.2 ^b^	**2.4 ^d^**	0.5 ^b^	0.2 ^c^	6.5 ^d^	0.5 ^c^	1.4 ^bc^
	Russia	1.0 ^c^	3.8 ^bc^	4.1 ^def^	0.6 ^b^	1.0 ^c^	**0.0 ^b^**	4.6 ^cd^	**0.0 ^b^**	0.2 ^c^	4.8 ^de^	0.5 ^c^	1.1 ^bc^
	Spain	**0.8 ^c^**	3.8 ^bc^	5.9 ^cde^	1.6 ^b^	1.4 ^c^	**0.0 ^b^**	4.1 ^cd^	0.3 ^b^	0.5 ^c^	3.8 ^de^	**0.3 ^c^**	**1.0 ^bc^**
	UK	1.3 ^c^	2.4 ^c^	4.6 ^def^	2.9 ^b^	1.4 ^c^	0.2 ^b^	4.3 ^cd^	0.6 ^b^	1.0 ^c^	4.4 ^de^	1.3 ^bc^	1.7 ^bc^
Y	Mexico	2.9 ^bc^	6.7 ^b^	12.2 ^b^	2.2 ^b^	3.3 ^bc^	**0.0 ^b^**	11.9 ^b^	**0.0 ^b^**	**0.0 ^c^**	11.1 ^b^	3.5 ^b^	3.0 ^b^
	Peru	1.4 ^c^	6.2 ^b^	14.0 ^b^	1.9 ^b^	2.9 ^bc^	**0.0 ^b^**	7.3 ^c^	**0.0 ^b^**	**0.0 ^c^**	5.4 ^de^	3.3 ^b^	3.5 ^b^
	Thailand	4.1 ^b^	2.5 ^c^	10.0 ^bc^	1.6 ^b^	2.2 ^c^	**0.0 ^b^**	12.1 ^b^	**0.0 ^b^**	**0.0 ^c^**	10.8 ^bc^	1.1 ^bc^	2.4 ^bc^
Z	Brazil	4.1 ^b^	3.0 ^cd^	5.1 ^def^	2.9 ^b^	0.6 ^cd^	0.2 ^b^	20.0 ^a^	**0.0 ^b^**	0.2 ^b^	4.3 ^de^	0.6 ^d^	3.5 ^b^
	India	2.2 ^bc^	**1.0 ^d^**	**1.3 ^f^**	1.9 ^b^	**0.0 ^d^**	0.3 ^b^	5.6 ^c^	0.2 ^b^	1.4 ^b^	**2.2 ^e^**	0.5 ^d^	1.3 ^b^
	South Africa	1.9 ^bc^	1.6 ^d^	1.6 ^ef^	2.5 ^b^	1.0 ^bcd^	0.2 ^b^	12.5 ^b^	0.2 ^b^	0.3 ^b^	4.3 ^de^	0.8 ^cd^	2.5 ^b^
		**Meat patty**											
W	USA	**8.7 ^a^**	**14.0 ^a^**	**18.4 ^a^**	**13.8 ^a^**	**13.0 ^a^**	**4.8 ^a^**	**21.7 ^a^**	**6.7 ^a^**	**7.9 ^a^**	**19.4 ^a^**	**11.4 ^a^**	**9.7 ^a^**
X	Australia	1.0 ^cd^	2.2 ^cd^	5.9 ^cdef^	2.5 ^b^	2.1 ^c^	0.2 ^c^	4.6 ^cd^	**0.0 ^b^**	0.2 ^b^	4.1 ^c^	3.2 ^bc^	1.7 ^bc^
	China	0.8 ^cd^	3.0 ^bcd^	7.0 ^bcde^	1.1 ^b^	1.3 ^c^	**0.0 ^c^**	3.7 ^cd^	0.2 ^b^	0.3 ^b^	4.6 ^c^	1.6 ^bcd^	1.0 ^bc^
	Japan	1.4 ^cd^	**0.8 ^d^**	4.6 ^cdefg^	1.1 ^b^	0.8 ^c^	0.2 ^c^	**1.9 ^cd^**	0.5 ^b^	0.3 ^b^	4.8 ^c^	0.6 ^cd^	1.7 ^bc^
	Russia	**0.2 ^d^**	2.1 ^cd^	3.2 ^efg^	**1.0 ^b^**	1.0 ^c^	**0.0 ^c^**	2.5 ^cd^	**0.0 ^b^**	**0.0 ^b^**	3.2 ^c^	**0.0 ^d^**	**0.8 ^bc^**
	Spain	1.1 ^cd^	2.9 ^bcd^	4.0 ^defg^	1.6 ^b^	1.0 ^c^	**0.0 ^c^**	3.2 ^cd^	**0.0 ^b^**	0.2 ^b^	2.9 ^c^	0.6 ^cd^	1.0 ^bc^
	UK	0.6 ^cd^	2.4 ^cd^	3.7 ^defg^	2.2 ^b^	2.1 ^c^	**0.0 ^c^**	3.3 ^cd^	0.8 ^b^	1.1 ^b^	3.5 ^c^	1.4 ^bcd^	1.4 ^bc^
Y	Mexico	2.5 ^bc^	4.9 ^bc^	7.5 ^bcd^	1.9 ^b^	1.7 ^c^	**0.0 ^c^**	10.0 ^b^	**0.0 ^b^**	**0.0 ^b^**	9.2 ^b^	2.5 ^bcd^	3.2 ^b^
	Peru	1.3 ^cd^	5.9 ^b^	10.6 ^b^	1.1 ^b^	3.7 ^bc^	**0.0 ^c^**	5.9 ^c^	**0.0 ^b^**	0.2 ^b^	4.8 ^c^	3.8 ^b^	2.4 ^bc^
	Thailand	4.0 ^b^	2.1 ^cd^	8.3 ^bc^	2.4 ^b^	2.4 ^bc^	**0.0 ^c^**	10.3 ^b^	**0.0 ^b^**	0.3 ^b^	10.0 ^b^	1.7 ^bcd^	1.7 ^bc^
Z	Brazil	3.8 ^bc^	1.9 ^cd^	5.1 ^cdefg^	2.7 ^b^	0.6 ^c^	0.3 ^b^	18.4 ^a^	**0.0 ^b^**	0.2 ^b^	4.0 ^c^	1.0 ^cd^	2.7 ^b^
	India	1.6 ^bcd^	1.3 ^d^	**1.4 ^g^**	1.4 ^b^	**0.3 ^c^**	0.5 ^b^	6.2 ^bcd^	**0.0 ^b^**	1.4 ^b^	**1.7 ^c^**	0.5 ^d^	1.3 ^b^
	South Africa	1.4 ^bcd^	1.4 ^d^	2.5 ^fg^	2.7 ^b^	0.5 ^c^	0.5 ^b^	11.0 ^b^	0.3 ^b^	0.5 ^b^	3.0 ^c^	0.6 ^cd^	1.4 ^b^

^(1)^ Cochran’s Q test was used to determine whether each insect powder’s selection frequency differed significantly between countries. There were significant differences between countries in the frequency of insect powder species selection for all food types (*p*-value < 0.0001). ^(2)^ Bold values indicate one country with a significantly higher or lower frequency of selecting a specific insect powder. ^(3)^ Each cluster was constructed among countries showing a similar trend in the frequency of selection of insect powders per food type. ^(4)^ Different letters within row indicate significant differences.

## Data Availability

The original contributions presented in this study are included in the article/Supplementary Materials. Further inquiries can be directed to the corresponding author.

## Supplementary Materials

The following supporting information can be downloaded at: https://www.mdpi.com/article/10.3390/foods14050841/s1, Table S1. Significant differences in the CATA frequency percentage indicating consumers’ willingness to consume specific insect powder according to demographic (gender; male and female) across the five food types arranged by country clusters. Table S2. Significant differences in the CATA frequency percentage indicating consumers’ willingness to consume specific insect powder according to demographic (age; 18–34, 35–54, and 54+) across the five food types arranged by country clusters. Table S3. Significant differences in the CATA frequency percentage indicating consumers’ willingness to consume specific insect powder according to demographic (the highest degree earned; High school or less and college graduate) across the five food types arranged by country clusters. Table S4. Significant differences in the CATA frequency percentage indicating consumers’ willingness to consume specific insect powder according to demographic (number of adults aged 18 or order in household; 1–2, and 3+) across the five food types arranged by country clusters. Table S5. Significant differences in the CATA frequency percentage indicating consumers’ willingness to consume specific insect powder according to demographic (number of children under 18 in household; none or any) across the five food types arranged by country clusters.
