# Supplementary material for: Cross-Cultural Comparison (13 Countries) of Consumers’ Willingness to Eat Specific Insect Powders in Five Food Types
Source: Foods. 2025 Feb 28;14(5):841. doi: 10.3390/foods14050841 (PMC11898450; doi:10.3390/foods14050841)
Supplement: Supplementary file 1 [file foods-14-00841-s001.zip › foods-3444812-supplementary.pdf]

Table S1. Significant differences in the CATA frequency percentage indicating consumers' willingness to consume specific insect powder according to demographic (gender; male and female) across the five food types arranged by country clusters.

Cluster	Country	Gender	Ant egg	Ant	Bee	Beetle	Caterpillar	Cockroach	Cricket	Fly larvae	Fly	Grasshopper	Mealworm	Wasp
			**Muffin or Bread**											
		Male	**15.0**	**22.0**	28.7	**21.7**	**17.2**	**8.3**	**30.9**	**11.5**	**11.5**	**28.3**	**17.2**	**14.3**
A	USA	Female	**5.7**	**12.0**	22.2	**10.1**	**8.2**	**4.1**	**19.9**	**2.8**	**3.2**	**17.1**	**6.3**	**7.3**
		p-value	**0.0001**	**0.001**	0.061	**<0.0001**	**0.001**	**0.030**	**0.002**	**<0.0001**	**<0.0001**	**0.001**	**<0.0001**	**0.004**
		Male	1.6	1.6	8.3	2.9	2.5	0.3	8.3	0.3	0.6	6.3	2.2	1.0
B	Australia	Female	0.6	3.2	7.0	3.2	1.6	0.3	6.3	0.0	0.3	4.8	2.5	1.3
		p-value	0.255	0.192	0.549	0.817	0.401	1.000	0.359	0.319	0.564	0.385	0.795	0.705
		Male	0.6	1.0	7.6	2.2	2.5	1.0	**4.1**	1.3	0.3	6.7	0.3	1.9
	Japan	Female	1.3	1.6	6.7	1.3	1.0	0.3	**1.0**	0.3	0.3	5.4	1.0	1.0
		p-value	0.413	0.478	0.643	0.362	0.129	0.317	**0.011**	0.179	1.000	0.504	0.317	0.315
		Male	1.3	2.9	**4.1**	1.6	1.3	0.0	4.1	0.0	0.3	6.7	0.3	1.6
	Russia	Female	0.6	2.9	**9.2**	0.6	0.3	0.0	2.9	0.0	0.3	5.4	0.6	1.3
		p-value	0.413	1.000	**0.011**	0.255	0.179	1.000	0.386	1.000	1.000	0.504	0.564	0.738
		Male	1.6	**5.1**	**9.2**	1.9	**1.6**	0.6	4.8	0.0	0.3	**5.1**	1.6	1.0
	Spain	Female	1.0	**1.9**	**5.1**	0.6	**0.0**	0.0	2.2	0.0	0.0	**1.6**	0.3	1.3
		p-value	0.478	**0.030**	**0.045**	0.155	**0.025**	0.158	0.083	1.000	0.319	**0.015**	0.101	0.705
		Male	2.2	4.5	8.0	3.2	1.6	0.6	7.3	1.0	1.0	4.8	2.2	2.2
	UK	Female	0.6	1.9	5.1	1.6	0.9	0.0	4.1	0.6	0.6	4.1	1.6	1.6
		p-value	0.092	0.067	0.141	0.188	0.472	0.156	0.083	0.650	0.650	0.687	0.553	0.553
		Male	0.6	**1.6**	12.7	1.0	1.0	0.6	3.2	0.0	0.6	4.8	2.9	1.3
C	China	Female	1.9	**5.7**	13.3	1.0	1.6	0.6	3.8	0.6	0.3	8.3	2.5	2.2
		p-value	0.155	**0.006**	0.813	1.000	0.478	1.000	0.665	0.158	0.564	0.076	0.807	0.362
		Male	3.5	7.3	16.8	1.3	1.3	0.3	12.7	0.0	0.0	13.0	3.5	2.5
	Mexico	Female	4.1	8.3	15.2	2.2	3.5	0.0	15.6	0.0	0.0	10.5	3.5	4.4
		p-value	0.678	0.656	0.588	0.362	0.068	0.319	0.304	1.000	1.000	0.323	1.000	0.193
		Male	**3.2**	8.6	16.5	2.2	3.5	0.3	7.9	0.0	0.0	7.0	3.8	1.9
	Peru	Female	**0.6**	5.7	17.1	1.3	2.2	0.0	6.7	0.0	0.0	5.4	3.5	1.9
		p-value	**0.020**	0.164	0.832	0.362	0.340	0.319	0.541	1.000	1.000	0.409	0.832	1.000
		Male	**7.6**	4.1	11.4	3.2	**4.1**	0.0	**15.6**	0.3	0.0	**12.7**	**2.5**	2.9
	Thailand	Female	**2.2**	2.2	16.5	1.3	**1.0**	0.0	**9.8**	0.0	0.0	**7.9**	**0.3**	1.3
		p-value	**0.002**	0.173	0.066	0.105	**0.011**	1.000	**0.031**	0.319	1.000	**0.050**	**0.019**	0.162

Cluster	Country	Gender	Ant egg	Ant	Bee	Beetle	Caterpillar	Cockroach	Cricket	Fly larvae	Fly	Grasshopper	Mealworm	Wasp
D	Brazil	Male	**7.0**	4.1	6.7	2.2	0.6	0.6	**41.9**	0.3	0.3	5.7	1.3	4.1
		Female	**2.9**	2.9	3.8	2.2	0.6	0.0	**26.3**	0.3	0.0	3.5	1.3	2.2
		p-value	**0.017**	0.386	0.108	1.000	1.000	0.158	**<0.0001**	1.000	0.319	0.184	1.000	0.173
	India	Male	4.1	0.6	2.2	3.8	0.0	1.0	**13.3**	1.6	**2.2**	2.2	0.3	3.2
		Female	3.2	0.3	2.2	1.6	0.0	0.6	**7.9**	0.6	**0.3**	1.9	0.6	2.8
		p-value	0.520	0.562	0.996	0.084	1.000	0.652	**0.027**	0.253	**0.033**	0.776	0.566	0.811
	South Africa	Male	2.9	1.3	2.5	3.8	**2.5**	0.3	**25.7**	0.6	0.6	3.8	1.6	3.2
		Female	2.9	1.3	2.2	2.5	**0.3**	0.0	**16.8**	0.0	0.0	3.2	1.0	2.5
		p-value	1.000	1.000	0.795	0.364	**0.019**	0.319	**0.006**	0.158	0.158	0.665	0.478	0.633
			**Cracker**											
A	USA	Male	**14.6**	**17.8**	**24.5**	**17.8**	**18.2**	**8.3**	**29.6**	**10.5**	**12.4**	**27.4**	**16.6**	**14.6**
		Female	**4.1**	**10.8**	**16.8**	**9.2**	**7.3**	**1.6**	**19.9**	**2.2**	**5.1**	**14.2**	**7.3**	**8.2**
		p-value	**<0.0001**	**0.011**	**0.016**	**0.001**	**<0.0001**	**0.0001**	**0.005**	**<0.0001**	**0.001**	**<0.0001**	**0.0001**	**0.011**
B	Australia	Male	1.6	2.2	5.1	1.6	1.6	0.3	6.0	0.0	0.6	5.4	2.2	2.2
		Female	0.3	1.3	4.4	2.2	1.3	0.0	4.4	0.0	0.0	2.5	1.0	1.6
		p-value	0.101	0.362	0.709	0.561	0.738	0.319	0.372	1.000	0.158	0.067	0.203	0.561
	Japan	Male	1.6	0.6	5.1	1.0	1.6	0.0	4.1	0.3	0.0	6.7	0.6	1.9
		Female	1.0	1.3	5.4	0.0	0.3	0.0	1.6	0.0	0.0	4.1	0.6	0.6
		p-value	0.478	0.413	0.859	0.083	0.101	1.000	0.056	0.319	1.000	0.159	1.000	0.155
	Russia	Male	0.3	1.6	3.2	1.6	1.0	0.0	4.4	0.0	0.3	2.9	0.0	1.0
		Female	0.3	2.2	4.8	1.0	0.3	0.0	2.5	0.0	0.0	4.4	0.6	1.0
		p-value	1.000	0.561	0.308	0.478	0.317	1.000	0.193	1.000	0.319	0.289	0.158	1.000
	Spain	Male	0.6	4.1	6.3	2.2	**1.9**	0.3	5.4	0.0	0.0	**5.7**	1.9	1.0
		Female	1.3	1.6	3.2	1.0	**0.0**	0.0	2.9	0.0	0.0	**1.9**	0.3	1.9
		p-value	0.413	0.056	0.062	0.203	**0.014**	0.319	0.110	1.000	1.000	**0.013**	0.058	0.315
	UK	Male	1.9	**2.9**	**6.4**	2.2	1.9	0.3	5.1	1.6	1.3	3.2	1.6	2.2
		Female	0.3	**0.6**	**2.5**	1.9	0.9	0.3	3.5	0.6	0.3	2.5	0.9	0.6
		p-value	0.057	**0.033**	**0.020**	0.771	0.310	0.998	0.318	0.252	0.176	0.624	0.472	0.092
C	China	Male	0.3	2.9	7.3	1.0	1.0	0.0	3.8	0.3	0.6	**3.2**	1.0	1.9
		Female	1.9	5.7	8.3	1.0	1.3	0.0	4.1	0.0	0.6	**7.0**	1.3	1.6
		p-value	0.058	0.077	0.656	1.000	0.705	1.000	0.839	0.319	1.000	**0.030**	0.705	0.762
	Mexico	Male	1.9	4.8	10.2	1.0	1.6	0.0	10.5	0.0	0.0	9.8	2.2	2.2
		Female	3.5	5.1	10.8	1.6	1.6	0.0	13.0	0.3	0.0	8.9	2.9	4.4
		p-value	0.220	0.854	0.795	0.478	1.000	1.000	0.323	0.319	1.000	0.682	0.613	0.121

Cluster	Country	Gender	Ant egg	Ant	Bee	Beetle	Caterpillar	Cockroach	Cricket	Fly larvae	Fly	Grasshopper	Mealworm	Wasp
		Male	**2.2**	6.3	11.7	2.2	3.8	0.0	8.6	0.3	0.0	6.7	4.1	2.5
	Peru	Female	**0.0**	4.4	11.7	1.0	2.5	0.0	6.0	0.0	0.0	4.4	3.5	1.9
		p-value	**0.008**	0.291	1.000	0.203	0.364	1.000	0.221	0.319	1.000	0.224	0.678	0.590
		Male	4.8	3.5	7.9	2.9	2.5	0.3	11.1	0.3	0.3	8.6	**2.5**	1.6
	Thailand	Female	2.9	1.3	12.1	1.3	1.0	0.0	7.0	0.0	0.0	7.3	**0.3**	1.0
		p-value	0.212	0.068	0.085	0.162	0.129	0.319	0.071	0.319	0.319	0.556	**0.019**	0.478
		Male	3.5	**3.8**	6.3	4.1	0.3	0.3	**22.2**	0.0	0.3	4.8	1.6	**3.8**
D	Brazil	Female	2.2	**1.0**	3.2	2.2	0.0	0.3	**15.2**	0.3	1.3	2.2	0.6	**0.6**
		p-value	0.340	**0.019**	0.062	0.173	0.319	1.000	**0.025**	0.319	0.179	0.083	0.255	**0.007**
		Male	3.2	2.2	2.2	2.2	0.0	0.3	8.3	0.3	**1.6**	1.0	0.3	2.2
	India	Female	1.3	0.9	1.6	0.6	0.3	0.3	7.0	0.0	**0.0**	1.6	0.6	0.6
		p-value	0.104	0.201	0.557	0.093	0.320	1.000	0.541	0.318	**0.025**	0.481	0.566	0.093
		Male	1.6	2.2	2.2	3.2	1.3	0.6	**16.5**	0.6	0.6	4.1	1.0	3.5
	South Africa	Female	1.3	1.0	1.9	2.5	0.6	0.0	**10.2**	0.0	0.3	2.9	0.6	2.2
		p-value	0.738	0.203	0.780	0.633	0.413	0.158	**0.019**	0.158	0.564	0.386	0.655	0.340
			**Cake**											
		Male	**12.7**	**16.9**	**23.2**	**18.2**	**16.9**	**7.0**	**25.5**	**8.9**	**13.1**	**23.2**	**15.0**	**13.7**
A	USA	Female	**6.0**	**9.8**	**16.1**	**8.9**	**7.6**	**1.9**	**16.1**	**2.8**	**5.1**	**13.0**	**7.6**	**6.6**
		p-value	**0.004**	**0.009**	**0.025**	**0.001**	**0.0001**	**0.002**	**0.004**	**0.001**	**0.0001**	**0.001**	**0.003**	**0.003**
		Male	1.9	2.2	6.7	2.2	2.2	0.0	5.1	0.0	1.0	5.4	2.9	1.9
B	Australia	Female	0.6	2.2	3.8	1.0	1.0	0.0	2.9	0.0	0.3	2.5	1.0	0.6
		p-value	0.155	1.000	0.108	0.203	0.203	1.000	0.154	1.000	0.317	0.067	0.081	0.155
		Male	0.6	1.0	4.1	**1.3**	1.3	0.6	**2.2**	0.3	0.3	**5.7**	0.6	1.6
	Japan	Female	0.6	1.0	4.8	**0.0**	0.6	0.0	**0.3**	0.0	0.0	**2.5**	0.6	1.0
		p-value	1.000	1.000	0.700	**0.045**	0.413	0.158	**0.033**	0.319	0.319	**0.045**	1.000	0.478
		Male	1.0	1.3	3.2	0.6	0.0	0.0	1.9	0.0	0.0	1.0	0.3	1.3
	Russia	Female	0.3	2.2	5.1	0.3	0.0	0.0	1.6	0.0	0.3	1.6	0.0	0.6
		p-value	0.317	0.362	0.230	0.564	1.000	1.000	0.762	1.000	0.319	0.478	0.319	0.413
		Male	1.0	3.2	5.7	2.2	1.6	0.3	3.2	0.3	0.3	**4.8**	1.0	1.3
	Spain	Female	1.3	1.3	3.5	0.6	0.3	0.0	1.6	0.3	0.0	**1.6**	1.0	1.3
		p-value	0.705	0.105	0.184	0.094	0.101	0.319	0.192	1.000	0.319	**0.023**	1.000	1.000
		Male	1.3	2.9	**4.8**	2.9	1.3	0.6	**4.8**	1.6	1.3	3.8	1.6	**1.9**
	UK	Female	0.3	1.3	**1.6**	1.6	0.9	0.0	**1.9**	0.3	0.6	1.9	0.6	**0.0**
		p-value	0.176	0.158	**0.022**	0.275	0.699	0.156	**0.044**	0.100	0.409	0.148	0.252	**0.014**

Cluster	Country	Gender	Ant egg	Ant	Bee	Beetle	Caterpillar	Cockroach	Cricket	Fly larvae	Fly	Grasshopper	Mealworm	Wasp
		Male	0.6	2.2	5.7	0.3	1.0	0.0	2.2	0.0	0.3	3.2	1.0	2.5
C	China	Female	1.9	3.8	6.0	0.6	1.3	0.3	1.6	0.0	1.0	6.0	1.9	0.6
		p-value	0.155	0.245	0.866	0.564	0.705	0.319	0.561	1.000	0.317	0.087	0.315	0.056
		Male	3.2	3.8	9.8	1.0	1.3	0.0	6.3	0.0	0.3	4.8	2.5	**1.0**
	Mexico	Female	1.3	5.4	9.5	1.6	1.0	0.0	8.3	0.0	0.0	5.7	2.5	**4.1**
		p-value	0.105	0.342	0.893	0.478	0.705	1.000	0.359	1.000	0.319	0.592	1.000	**0.011**
		Male	2.2	**6.3**	11.4	2.2	2.9	0.3	5.1	0.0	0.0	6.3	4.1	2.5
	Peru	Female	1.3	**2.9**	11.1	0.6	2.2	0.0	3.5	0.0	0.0	3.2	2.2	1.3
		p-value	0.362	**0.037**	0.900	0.094	0.613	0.319	0.326	1.000	1.000	0.062	0.173	0.244
		Male	4.4	2.5	8.6	1.6	**3.8**	0.3	7.0	0.0	0.3	6.0	1.9	1.3
	Thailand	Female	3.2	0.6	9.2	1.0	**0.3**	0.0	4.8	0.0	0.0	4.8	1.0	1.0
		p-value	0.406	0.056	0.780	0.478	**0.002**	0.319	0.236	1.000	0.319	0.481	0.315	0.705
		Male	3.5	3.5	**7.3**	3.8	0.6	0.3	**21.6**	0.0	0.0	3.8	1.6	**4.8**
D	Brazil	Female	1.6	1.9	**2.9**	2.9	0.3	0.0	**14.3**	0.6	0.0	2.2	0.6	**1.6**
		p-value	0.129	0.220	**0.011**	0.506	0.564	0.319	**0.017**	0.158	1.000	0.245	0.255	**0.023**
		Male	2.5	1.0	2.5	**2.2**	0.6	0.0	3.8	0.3	1.6	1.0	0.0	1.0
	India	Female	1.6	0.3	0.6	**0.0**	0.0	0.3	3.2	0.0	0.6	1.3	0.0	0.0
		p-value	0.398	0.315	0.055	**0.008**	0.157	0.320	0.660	0.318	0.253	0.708	1.000	0.083
		Male	1.6	1.3	2.9	2.9	1.3	0.3	8.6	0.6	0.6	2.5	1.3	2.9
	South Africa	Female	0.6	1.3	2.9	1.3	0.3	0.0	7.3	0.0	0.3	1.9	0.3	1.3
		p-value	0.255	1.000	1.000	0.162	0.179	0.319	0.556	0.158	0.564	0.590	0.179	0.162
			**Salty snack food**											
		Male	**13.1**	**22.0**	**28.0**	**19.7**	**16.2**	**7.6**	**28.0**	**11.1**	**11.5**	**28.7**	**14.3**	**15.3**
W	USA	Female	**5.1**	**11.4**	**17.1**	**9.8**	**8.5**	**1.6**	**20.6**	**2.8**	**3.8**	**14.6**	**7.0**	**7.6**
		p-value	**0.0001**	**0.0001**	**0.001**	**0.0001**	**0.003**	**0.0001**	**0.029**	**<0.0001**	**0.0001**	**<0.0001**	**0.003**	**0.002**
		Male	1.6	2.9	6.3	2.5	1.6	0.0	6.0	0.0	0.3	4.8	2.9	2.2
X	Australia	Female	0.6	2.2	4.4	1.6	0.6	0.0	6.0	0.0	0.3	4.4	1.6	1.6
		p-value	0.255	0.613	0.291	0.401	0.255	1.000	1.000	1.000	1.000	0.850	0.280	0.561
		Male	**0.3**	**1.9**	7.0	2.2	0.6	0.3	3.8	0.3	1.0	6.3	0.6	1.9
	China	Female	**2.2**	**6.3**	8.3	1.3	1.0	0.3	2.2	0.0	0.0	7.3	1.6	0.6
		p-value	**0.033**	**0.005**	0.549	0.362	0.655	1.000	0.245	0.319	0.083	0.636	0.255	0.155
		Male	1.3	1.9	7.0	0.3	2.2	0.3	3.2	1.0	0.0	7.9	0.3	2.2
	Japan	Female	1.0	1.6	5.7	0.3	0.6	0.0	1.6	0.0	0.3	5.1	0.6	0.6
		p-value	0.705	0.762	0.514	1.000	0.094	0.319	0.192	0.083	0.319	0.147	0.564	0.094

Cluster	Country	Gender	Ant egg	Ant	Bee	Beetle	Caterpillar	Cockroach	Cricket	Fly larvae	Fly	Grasshopper	Mealworm	Wasp
		Male	1.3	4.1	3.2	1.0	**1.9**	0.0	5.4	0.0	0.3	5.7	0.6	1.6
	Russia	Female	0.6	3.5	5.1	0.3	**0.0**	0.0	3.8	0.0	0.0	3.8	0.3	0.6
		p-value	0.413	0.678	0.230	0.317	**0.014**	1.000	0.342	1.000	0.319	0.262	0.564	0.255
		Male	0.6	**5.7**	7.6	1.9	1.9	0.0	**5.7**	0.6	1.0	**5.7**	0.6	0.6
	Spain	Female	1.0	**1.9**	4.1	1.3	1.0	0.0	**2.5**	0.0	0.0	**1.9**	0.0	1.3
		p-value	0.655	**0.013**	0.063	0.525	0.315	1.000	**0.045**	0.158	0.083	**0.013**	0.158	0.413
		Male	1.9	**3.8**	**6.4**	2.5	1.6	0.0	5.4	0.3	1.3	4.1	1.9	**3.2**
	UK	Female	0.6	**0.9**	**2.8**	3.2	1.3	0.3	3.2	0.9	0.6	4.7	0.6	**0.3**
		p-value	0.153	**0.018**	**0.035**	0.643	0.731	0.320	0.164	0.320	0.409	0.712	0.153	**0.006**
		Male	2.5	7.3	12.7	1.6	2.2	0.0	11.4	0.0	0.0	11.7	3.5	2.9
Y	Mexico	Female	3.2	6.0	11.7	2.9	4.4	0.0	12.4	0.0	0.0	10.5	3.5	3.2
		p-value	0.633	0.524	0.716	0.280	0.121	1.000	0.713	1.000	1.000	0.613	1.000	0.817
		Male	2.2	**8.9**	14.3	2.5	2.9	0.0	7.6	0.0	0.0	5.7	3.8	3.2
	Peru	Female	0.6	**3.5**	13.7	1.3	2.9	0.0	7.0	0.0	0.0	5.1	2.9	3.8
		p-value	0.094	**0.005**	0.819	0.244	1.000	1.000	0.760	1.000	1.000	0.725	0.506	0.665
		Male	**5.7**	2.2	**7.6**	2.2	3.2	0.0	**15.2**	0.0	0.0	12.1	1.9	3.5
	Thailand	Female	**2.5**	2.9	**12.4**	1.0	1.3	0.0	**8.9**	0.0	0.0	9.5	0.3	1.3
		p-value	**0.045**	0.613	**0.047**	0.203	0.105	1.000	**0.015**	1.000	1.000	0.305	0.058	0.068
		Male	**6.3**	3.2	6.0	3.8	1.0	0.3	**25.7**	0.0	0.0	5.4	0.6	**5.1**
Z	Brazil	Female	**1.9**	2.9	4.1	1.9	0.3	0.0	**14.3**	0.0	0.3	3.2	0.6	**1.9**
		p-value	**0.005**	0.817	0.277	0.152	0.317	0.319	**0.0001**	1.000	0.319	0.169	1.000	**0.030**
		Male	**3.5**	1.6	1.3	**3.5**	0.0	0.3	7.0	0.3	1.9	3.2	1.0	1.6
	India	Female	**0.9**	0.3	1.3	**0.3**	0.0	0.3	4.4	0.0	0.9	1.6	0.0	1.3
		p-value	**0.030**	0.101	0.997	**0.004**	1.000	1.000	0.167	0.318	0.312	0.190	0.083	0.734
		Male	1.9	1.6	2.2	2.9	1.6	0.3	13.7	0.3	0.6	5.1	1.3	**4.1**
	South Africa	Female	1.9	1.6	1.0	2.2	0.3	0.0	11.4	0.0	0.0	3.5	0.3	**1.0**
		p-value	1.000	1.000	0.203	0.613	0.101	0.319	0.400	0.319	0.158	0.326	0.179	**0.011**
			**Meat patty**											
		Male	**13.1**	**17.8**	**23.6**	**18.5**	**17.5**	**7.0**	**27.4**	**10.2**	**12.4**	**26.1**	**15.0**	**14.3**
W	USA	Female	**4.4**	**10.1**	**13.3**	**9.2**	**8.5**	**2.5**	**16.1**	**3.2**	**3.5**	**12.7**	**7.9**	**5.1**
		p-value	**0.0001**	**0.005**	**0.001**	**0.001**	**0.001**	**0.008**	**0.001**	**0.0001**	**<0.0001**	**<0.0001**	**0.005**	**<0.0001**
		Male	1.3	2.5	7.3	2.9	2.9	0.0	6.0	0.0	0.3	5.1	3.8	2.2
X	Australia	Female	0.6	1.9	4.4	2.2	1.3	0.3	3.2	0.0	0.0	3.2	2.5	1.3
		p-value	0.413	0.590	0.128	0.613	0.162	0.319	0.087	1.000	0.319	0.230	0.364	0.362

Cluster	Country	Gender	Ant egg	Ant	Bee	Beetle	Caterpillar	Cockroach	Cricket	Fly larvae	Fly	Grasshopper	Mealworm	Wasp
		Male	**0.0**	1.9	7.3	1.3	0.6	0.0	3.5	0.0	0.3	4.4	1.6	1.3
	China	Female	**1.6**	4.1	6.7	1.0	1.9	0.0	3.8	0.3	0.3	4.8	1.6	0.6
		p-value	**0.025**	0.103	0.755	0.705	0.155	1.000	0.832	0.319	1.000	0.850	1.000	0.413
		Male	1.9	0.6	4.4	1.3	1.3	2.2	2.9	0.6	0.6	**6.7**	0.3	0.3
	Japan	Female	1.0	1.0	4.8	1.0	0.3	1.3	1.0	0.3	0.0	**2.9**	1.0	0.0
		p-value	0.315	0.655	0.850	0.705	0.179	0.362	0.081	0.564	0.158	**0.025**	0.317	0.319
		Male	0.3	1.6	2.2	1.3	1.0	0.0	3.5	0.0	0.0	3.8	0.0	1.0
	Russia	Female	0.0	2.5	4.1	0.6	1.0	0.0	1.6	0.0	0.0	2.5	0.0	0.6
		p-value	0.319	0.401	0.173	0.413	1.000	1.000	0.129	1.000	1.000	0.364	1.000	0.655
		Male	1.6	3.8	5.4	2.5	1.6	0.0	**4.8**	0.0	0.3	**4.4**	0.6	1.0
	Spain	Female	0.6	1.9	2.5	0.6	0.3	0.0	**1.6**	0.0	0.0	**1.3**	0.6	1.0
		p-value	0.255	0.152	0.067	0.056	0.101	1.000	**0.023**	1.000	0.319	**0.017**	1.000	1.000
		Male	1.0	3.5	**5.4**	2.5	2.5	0.0	**4.8**	1.3	1.6	4.1	2.2	2.2
	UK	Female	0.3	1.3	**1.9**	1.9	1.6	0.0	**1.9**	0.3	0.6	2.8	0.6	0.6
		p-value	0.314	0.066	**0.019**	0.581	0.395	1.000	**0.044**	0.176	0.252	0.378	0.092	0.092
		Male	2.2	4.4	7.9	1.0	2.2	0.0	7.9	0.0	0.0	9.8	2.2	2.2
Y	Mexico	Female	2.9	5.4	7.0	2.9	1.3	0.0	12.1	0.0	0.0	8.6	2.9	4.1
		p-value	0.613	0.581	0.650	0.081	0.362	1.000	0.085	1.000	1.000	0.582	0.613	0.173
		Male	**2.2**	**8.6**	11.1	1.6	4.1	0.0	7.3	0.0	0.3	5.7	4.8	3.2
	Peru	Female	**0.3**	**3.2**	10.2	0.6	3.2	0.0	4.4	0.0	0.0	3.8	2.9	1.6
		p-value	**0.033**	**0.004**	0.699	0.255	0.525	1.000	0.128	1.000	0.319	0.262	0.212	0.192
		Male	5.1	2.9	7.9	**4.4**	3.5	0.0	12.1	0.0	0.6	**12.7**	**2.9**	2.5
	Thailand	Female	2.9	1.3	8.6	**0.3**	1.3	0.0	8.6	0.0	0.0	**7.3**	**0.6**	1.0
		p-value	0.154	0.162	0.773	**0.001**	0.068	1.000	0.150	1.000	0.158	**0.024**	**0.033**	0.129
		Male	**5.7**	2.5	5.4	3.5	1.0	0.3	**22.2**	0.0	0.0	5.1	1.6	**4.1**
Z	Brazil	Female	**1.9**	1.3	4.8	1.9	0.3	0.3	**14.6**	0.0	0.3	2.9	0.3	**1.3**
		p-value	**0.013**	0.244	0.717	0.220	0.317	1.000	**0.014**	1.000	0.319	0.154	0.101	**0.027**
		Male	1.6	1.9	1.9	**2.9**	0.6	0.3	7.6	0.0	1.6	2.2	0.3	1.6
	India	Female	1.6	0.9	1.3	**0.3**	0.0	0.6	5.1	0.0	1.3	1.6	0.9	0.9
		p-value	0.997	0.312	0.521	**0.011**	0.157	0.566	0.188	1.000	0.734	0.557	0.318	0.475
		Male	1.6	2.2	2.5	3.8	1.0	1.0	13.3	0.6	0.6	3.5	1.0	1.6
	South Africa	Female	1.3	0.6	2.5	1.6	0.0	0.0	8.6	0.0	0.3	2.5	0.3	1.3
		p-value	0.738	0.094	1.000	0.086	0.083	0.083	0.056	0.158	0.564	0.485	0.317	0.738

[1]The Mann-Whitney U test was performed to investigate whether the frequency of insect powder selection in each country differed significantly according the gender.

[2]Bold highlights means that there is a significant difference (p-value < 0.05).

Table S2. Significant differences in the CATA frequency percentage indicating consumers' willingness to consume specific insect powder according to demographic (age; 18-34, 35-54, and 54+) across the five food types arranged by country clusters.

Cluster	Country	Age	Ant egg	Ant	Bee	Beetle	Caterpillar	Cockroach	Cricket	Fly larvae	Fly	Grasshopper	Mealworm	Wasp
			**Muffin or Bread**											
A	USA	18-34	12.4	19.1	**23.0**^{b}	16.3	13.9	**10.0**^{a}	27.3	8.1	10.0	22.0	9.6	12.9
		35-54	12.3	19.4	**31.8**^{a}	18.0	15.2	**5.7**^{ab}	25.6	7.1	7.6	23.7	15.2	11.4
		55+	6.2	12.4	**21.4**^{b}	13.3	9.0	**2.9**^{b}	23.3	6.2	4.3	22.4	10.5	8.1
		p-value	0.055	0.094	**0.032**	0.415	0.140	**0.009**	0.650	0.742	0.075	0.910	0.161	0.267
B	Australia	18-34	1.0	1.9	8.1	3.8	**4.3**^{a}	0.0	10.0	0.5	0.5	7.6	2.9	1.9
		35-54	1.4	3.3	6.7	3.3	**1.4**^{b}	0.0	7.1	0.0	0.5	4.8	1.0	1.0
		55+	1.0	1.9	8.1	1.9	**0.5**^{b}	1.0	4.8	0.0	0.5	4.3	3.3	0.5
		p-value	0.866	0.541	0.817	0.494	**0.017**	0.135	0.119	0.368	1.000	0.273	0.239	0.364
	Japan	18-34	1.9	1.9	4.3	2.4	2.9	1.4	2.9	1.9	1.0	5.2	1.4	2.4
		35-54	0.5	1.0	9.0	2.4	1.0	0.5	1.4	0.0	0.0	4.8	0.5	0.5
		55+	0.5	1.0	8.1	0.5	1.4	0.0	3.3	0.5	0.0	8.1	0.0	1.4
		p-value	0.220	0.603	0.134	0.228	0.301	0.172	0.435	0.073	0.135	0.301	0.172	0.259
	Russia	18-34	0.0	2.4	5.7	1.9	1.4	0.0	4.3	0.0	1.0	6.7	1.0	1.9
		35-54	1.4	2.4	7.6	0.5	1.0	0.0	3.3	0.0	0.0	6.2	0.5	1.4
		55+	1.4	3.8	6.7	1.0	0.0	0.0	2.9	0.0	0.0	5.2	0.0	1.0
		p-value	0.220	0.598	0.737	0.364	0.244	1.000	0.720	1.000	0.135	0.822	0.367	0.713
	Spain	18-34	1.0	3.8	4.8	0.0	0.5	0.5	3.3	0.0	0.0	2.4	1.0	1.4
		35-54	1.4	4.3	8.1	1.9	1.0	0.5	3.8	0.0	0.5	2.9	0.5	0.5
		55+	1.4	2.4	8.6	1.9	1.0	0.0	3.3	0.0	0.0	4.8	1.4	1.4
		p-value	0.881	0.543	0.256	0.132	0.818	0.606	0.954	1.000	0.368	0.356	0.604	0.562
	UK	18-34	1.9	**4.8**^{a}	6.2	2.9	2.4	1.0	7.6	1.4	1.4	4.3	1.4	1.9
		35-54	1.4	**4.3**^{a}	7.1	3.3	1.4	0.0	4.8	1.0	0.5	5.7	2.9	2.4
		55+	1.0	**0.5**^{b}	6.2	1.0	0.0	0.0	4.8	0.0	0.5	3.3	1.4	1.4
		p-value	0.713	**0.023**	0.901	0.239	0.091	0.135	0.347	0.244	0.447	0.492	0.466	0.775
C	China	18-34	1.4	2.9	13.3	1.9	1.9	0.0	2.9	1.0	0.5	5.7	4.3	2.9
		35-54	0.5	2.4	11.9	0.5	1.4	0.5	3.8	0.0	0.0	5.7	2.4	1.0
		55+	1.9	5.7	13.8	0.5	0.5	1.4	3.8	0.0	1.0	8.1	1.4	1.4
		p-value	0.413	0.144	0.834	0.220	0.413	0.172	0.829	0.135	0.367	0.521	0.185	0.301

Cluster	Country	Age	Ant egg	Ant	Bee	Beetle	Caterpillar	Cockroach	Cricket	Fly larvae	Fly	Grasshopper	Mealworm	Wasp
	Mexico	18-34	2.4	8.6	15.7	1.9	2.4	0.5	14.3	0.0	0.0	9.5	2.4	3.3
		35-54	4.3	8.1	14.3	1.9	1.4	0.0	14.8	0.0	0.0	11.4	4.3	2.9
		55+	4.8	6.7	18.1	1.4	3.3	0.0	13.3	0.0	0.0	14.3	3.8	4.3
		p-value	0.403	0.750	0.562	0.912	0.441	0.368	0.913	1.000	1.000	0.313	0.543	0.720
	Peru	18-34	0.5	5.7	13.3	1.4	**0.5[b]**	0.5	7.1	0.0	0.0	4.8	2.9	**0.5[b]**
		35-54	2.9	7.1	16.7	1.4	**3.8[a]**	0.0	7.6	0.0	0.0	5.7	3.8	**1.0[b]**
		55+	2.4	8.6	20.5	2.4	**4.3[a]**	0.0	7.1	0.0	0.0	8.1	4.3	**4.3[a]**
		p-value	0.168	0.525	0.148	0.691	**0.039**	0.368	0.977	1.000	1.000	0.345	0.730	**0.008**
	Thailand	18-34	**1.9[b]**	2.4	**8.1[b]**	1.0	**0.5[b]**	0.0	10.5	0.0	0.0	**5.7[b]**	0.0	1.0
		35-54	**6.7[a]**	2.4	**15.7[a]**	2.9	**1.0[b]**	0.0	12.9	0.0	0.0	**10.5[ab]**	1.9	2.9
		55+	**6.2[a]**	4.8	**18.1[a]**	2.9	**6.2[a]**	0.0	14.8	0.5	0.0	**14.8[a]**	2.4	2.4
		p-value	**0.046**	0.276	**0.009**	0.311	**0.0001**	1.000	0.418	0.368	1.000	**0.010**	0.094	0.361
D	Brazil	18-34	6.7	3.3	5.2	1.4	1.4	0.0	37.1	0.5	0.5	5.2	1.4	2.4
		35-54	3.3	3.3	4.3	2.9	0.5	0.5	34.8	0.5	0.0	3.8	1.4	3.3
		55+	4.8	3.8	6.2	2.4	0.0	0.5	30.5	0.0	0.0	4.8	1.0	3.8
		p-value	0.286	0.954	0.682	0.600	0.172	0.606	0.345	0.606	0.368	0.777	0.881	0.697
	India	18-34	4.3	**0.0[b]**	2.8	3.8	0.0	1.4	13.7	1.9	1.9	1.9	0.5	5.2
		35-54	4.3	**1.4[a]**	2.9	1.9	0.0	0.5	9.0	1.0	1.4	2.9	0.0	1.4
		55+	2.4	**0.0[b]**	1.0	2.4	0.0	0.5	9.0	0.5	0.5	1.4	1.0	2.4
		p-value	0.489	**0.049**	0.313	0.462	1.000	0.450	0.196	0.368	0.416	0.576	0.366	0.061
	South Africa	18-34	3.3	0.5	2.4	3.3	1.9	0.0	**21.4[ab]**	0.5	0.5	**1.9[b]**	1.4	**1.4[b]**
		35-54	1.0	1.9	2.4	1.9	0.5	0.0	**15.2[b]**	0.0	0.0	**2.4[b]**	0.5	**1.9[b]**
		55+	4.3	1.4	2.4	4.3	1.9	0.5	**27.1[a]**	0.5	0.5	**6.2[a]**	1.9	**5.2[a]**
		p-value	0.108	0.413	1.000	0.375	0.363	0.368	**0.012**	0.606	0.606	**0.032**	0.413	**0.039**
			**Cracker**											
A	USA	18-34	10.0	**18.2[a]**	**19.6[ab]**	11.0	13.4	**7.7[a]**	26.8	6.2	12.0	20.6	9.6	**14.8[a]**
		35-54	11.4	**15.2[ab]**	**26.1[a]**	16.1	13.3	**4.7[ab]**	26.5	7.6	9.0	21.8	15.6	**12.3[ab]**
		55+	6.7	**9.5[b]**	**16.2[b]**	13.3	11.4	**2.4[b]**	21.0	5.2	5.2	20.0	10.5	**7.1[b]**
		p-value	0.233	**0.037**	**0.040**	0.309	0.795	**0.044**	0.293	0.612	0.051	0.898	0.117	**0.042**
B	Australia	18-34	1.4	1.4	3.3	2.4	1.9	0.5	5.2	0.0	0.5	3.8	2.4	2.9
		35-54	1.0	2.4	5.7	1.9	1.4	0.0	6.2	0.0	0.5	4.8	0.5	1.9
		55+	0.5	1.4	5.2	1.4	1.0	0.0	4.3	0.0	0.0	3.3	1.9	1.0
		p-value	0.604	0.691	0.480	0.775	0.713	0.368	0.682	1.000	0.606	0.747	0.267	0.361

Cluster	Country	Age	Ant egg	Ant	Bee	Beetle	Caterpillar	Cockroach	Cricket	Fly larvae	Fly	Grasshopper	Mealworm	Wasp
	Japan	18-34	1.4	1.0	4.3	0.5	1.0	0.0	3.3	0.5	0.0	3.8	1.4	1.4
		35-54	1.4	1.0	5.7	0.5	1.4	0.0	1.9	0.0	0.0	4.3	0.5	1.4
		55+	1.0	1.0	5.7	0.5	0.5	0.0	3.3	0.0	0.0	8.1	0.0	1.0
		p-value	0.881	1.000	0.750	1.000	0.604	1.000	0.598	0.368	1.000	0.104	0.172	0.881
	Russia	18-34	0.5	1.9	2.9	1.9	1.4	0.0	2.9	0.0	0.5	3.3	1.0	0.5
		35-54	0.0	1.4	3.3	1.0	0.5	0.0	5.2	0.0	0.0	4.8	0.0	1.0
		55+	0.5	2.4	5.7	1.0	0.0	0.0	2.4	0.0	0.0	2.9	0.0	1.4
		p-value	0.606	0.775	0.275	0.603	0.172	1.000	0.233	1.000	0.368	0.557	0.135	0.604
	Spain	18-34	0.0	3.3	3.8	1.4	0.5	0.0	3.3	0.0	0.0	3.8	0.5	1.0
		35-54	1.9	2.9	5.7	1.9	1.9	0.5	5.7	0.0	0.0	3.8	1.4	1.4
		55+	1.0	2.4	4.8	1.4	0.5	0.0	3.3	0.0	0.0	3.8	1.4	1.9
		p-value	0.133	0.843	0.657	0.904	0.220	0.368	0.367	1.000	1.000	1.000	0.562	0.713
	UK	18-34	1.9	**3.3**[a]	6.2	2.4	1.9	1.0	5.7	2.4	1.4	3.3	1.9	1.9
		35-54	1.4	**1.9**[ab]	4.3	2.4	2.4	0.0	3.3	1.0	0.5	4.3	1.4	1.4
		55+	0.0	**0.0**[b]	2.9	1.4	0.0	0.0	3.8	0.0	0.5	1.0	0.5	1.0
		p-value	0.153	**0.033**	0.251	0.731	0.094	0.135	0.444	0.065	0.447	0.108	0.413	0.713
C	China	18-34	1.9	3.8	7.6	1.9	2.4	0.0	3.8	0.0	1.0	5.7	1.9	1.9
		35-54	0.5	2.9	4.8	0.5	0.0	0.0	4.3	0.0	0.0	4.8	0.5	0.5
		55+	1.0	6.2	11.0	0.5	1.0	0.0	3.8	0.5	1.0	4.8	1.0	2.9
		p-value	0.364	0.222	0.060	0.220	0.065	1.000	0.959	0.368	0.366	0.877	0.364	0.173
	Mexico	18-34	1.4	4.8	9.5	2.4	2.4	0.0	13.8	0.0	0.0	8.1	1.9	2.9
		35-54	3.3	4.3	8.1	1.4	0.5	0.0	10.5	0.0	0.0	9.0	1.4	2.9
		55+	3.3	5.7	13.8	0.0	1.9	0.0	11.0	0.5	0.0	11.0	4.3	4.3
		p-value	0.381	0.789	0.138	0.091	0.267	1.000	0.518	0.368	1.000	0.593	0.137	0.642
	Peru	18-34	0.5	**2.4**[b]	**6.2**[b]	1.0	1.9	0.0	5.2	0.0	0.0	**1.9**[b]	2.4	1.0
		35-54	0.5	**6.2**[ab]	**12.4**[a]	1.0	3.8	0.0	9.5	0.0	0.0	**7.1**[a]	3.8	1.9
		55+	2.4	**7.6**[a]	**16.7**[a]	2.9	3.8	0.0	7.1	0.5	0.0	**7.6**[a]	5.2	3.8
		p-value	0.099	**0.049**	**0.004**	0.197	0.438	1.000	0.240	0.368	1.000	**0.018**	0.311	0.130
	Thailand	18-34	1.9	1.0	7.1	0.5	**0.0**[b]	0.0	**4.8**[b]	0.0	0.0	4.8	0.0	**0.5**[b]
		35-54	4.3	2.9	10.5	3.3	**1.4**[ab]	0.0	**11.9**[a]	0.0	0.0	10.0	2.4	**2.9**[a]
		55+	5.2	3.3	12.4	2.4	**3.8**[a]	0.5	**10.5**[a]	0.5	0.5	9.0	1.9	**0.5**[b]
		p-value	0.185	0.239	0.194	0.111	**0.011**	0.368	**0.026**	0.368	0.368	0.107	0.094	**0.042**
D	Brazil	18-34	2.9	3.8	2.4	2.4	0.0	0.0	18.6	0.5	1.4	3.3	0.5	0.5
		35-54	3.3	1.9	4.8	3.8	0.5	1.0	20.5	0.0	1.0	3.3	1.9	2.4
		55+	2.4	1.4	7.1	3.3	0.0	0.0	17.1	0.0	0.0	3.8	1.0	3.8

		p-value	0.843	0.239	0.073	0.697	0.368	0.135	0.680	0.368	0.244	0.954	0.364	0.067
Cluster	Country	Age	Ant egg	Ant	Bee	Beetle	Caterpillar	Cockroach	Cricket	Fly larvae	Fly	Grasshopper	Mealworm	Wasp
	India	18-34	2.4	2.4	0.9	1.9	0.0	0.0	10.4	0.0	0.0	1.4	0.5	1.9
		35-54	1.9	1.4	2.9	1.0	0.5	0.5	7.6	0.5	1.4	1.4	0.5	1.9
		55+	2.4	1.0	1.9	1.4	0.0	0.5	4.8	0.0	1.0	1.0	0.5	0.5
		p-value	0.931	0.496	0.358	0.717	0.367	0.605	0.091	0.367	0.243	0.883	1.000	0.365
	South Africa	18-34	1.0	1.0	1.4	3.3	1.0	0.0	16.2	0.5	0.5	**2.4**[b]	0.5	**1.9**[b]
		35-54	0.5	1.0	1.4	1.4	0.0	0.0	11.4	0.0	0.5	**1.9**[b]	0.5	**1.4**[b]
		55+	2.9	2.9	3.3	3.8	1.9	1.0	12.4	0.5	0.5	**6.2**[a]	1.4	**5.2**[a]
		p-value	0.094	0.197	0.285	0.301	0.133	0.135	0.316	0.606	1.000	**0.032**	0.447	**0.039**
			**Cake**											
A	USA	18-34	9.1	**13.4**[ab]	**14.8**[b]	12.4	13.4	6.2	23.0	5.3	11.0	16.7	**8.1**[b]	10.5
		35-54	12.3	**17.5**[a]	**28.4**[a]	15.2	12.3	4.3	20.4	7.6	10.0	19.4	**16.1**[a]	11.8
		55+	6.7	**9.0**[b]	**15.7**[b]	12.9	11.0	2.9	19.0	4.8	6.2	18.1	**9.5**[b]	8.1
		p-value	0.136	**0.038**	**0.0001**	0.678	0.746	0.245	0.604	0.423	0.196	0.775	**0.022**	0.434
B	Australia	18-34	1.9	2.9	5.7	2.4	1.4	0.0	4.3	0.0	0.5	4.3	2.9	1.9
		35-54	1.4	2.4	4.8	1.0	2.4	0.0	4.8	0.0	1.0	3.8	1.0	1.0
		55+	0.5	1.4	5.2	1.4	1.0	0.0	2.9	0.0	0.5	3.8	1.9	1.0
		p-value	0.413	0.600	0.909	0.492	0.492	1.000	0.582	1.000	0.778	0.959	0.361	0.603
	Japan	18-34	1.4	1.9	**1.4**[b]	0.5	1.4	1.0	1.9	0.5	0.0	2.9	1.0	1.9
		35-54	0.5	0.0	**6.2**[a]	1.0	0.5	0.0	0.5	0.0	0.0	4.3	1.0	1.0
		55+	0.0	1.0	**5.7**[a]	0.5	1.0	0.0	1.4	0.0	0.5	5.2	0.0	1.0
		p-value	0.172	0.133	**0.034**	0.778	0.604	0.135	0.413	0.368	0.368	0.467	0.366	0.603
	Russia	18-34	1.0	1.9	3.3	1.0	0.0	0.0	1.9	0.0	0.5	1.0	0.5	0.5
		35-54	1.0	1.9	4.3	0.5	0.0	0.0	1.9	0.0	0.0	1.9	0.0	1.0
		55+	0.0	1.4	4.8	0.0	0.0	0.0	1.4	0.0	0.0	1.0	0.0	1.4
		p-value	0.366	0.912	0.756	0.367	1.000	1.000	0.912	1.000	0.368	0.603	0.368	0.604
	Spain	18-34	0.0	2.4	4.8	1.0	1.0	0.0	1.9	0.0	0.5	2.9	0.5	1.0
		35-54	1.9	2.4	4.8	1.9	1.4	0.5	2.9	1.0	0.0	3.3	1.4	1.0
		55+	1.4	1.9	4.3	1.4	0.5	0.0	2.4	0.0	0.0	3.3	1.0	1.9
		p-value	0.153	0.930	0.965	0.713	0.604	0.368	0.815	0.135	0.368	0.950	0.604	0.603
	UK	18-34	1.0	2.9	4.8	2.4	1.0	1.0	5.2	1.9	**2.4**[a]	3.3	1.0	1.0
		35-54	1.4	2.4	3.3	2.4	2.4	0.0	2.9	1.0	**0.5**[b]	2.4	1.4	1.4
		55+	0.0	1.0	1.4	1.9	0.0	0.0	1.9	0.0	**0.0**[b]	2.9	1.0	0.5
		p-value	0.244	0.361	0.148	0.930	0.065	0.135	0.147	0.133	**0.029**	0.843	0.866	0.604

Cluster	Country	Age	Ant egg	Ant	Bee	Beetle	Caterpillar	Cockroach	Cricket	Fly larvae	Fly	Grasshopper	Mealworm	Wasp
C	China	18-34	2.4	3.8	4.3	1.0	1.9	0.0	1.9	0.0	1.0	5.7	1.9	1.0
		35-54	0.5	2.4	5.2	0.5	0.5	0.0	2.9	0.0	0.0	3.8	1.0	1.0
		55+	1.0	2.9	8.1	0.0	1.0	0.5	1.0	0.0	1.0	4.3	1.4	2.9
		p-value	0.193	0.684	0.225	0.367	0.364	0.368	0.361	1.000	0.366	0.626	0.713	0.197
	Mexico	18-34	2.9	4.3	9.5	**2.9**^{a}	1.0	0.0	8.1	0.0	0.5	3.8	1.9	1.4
		35-54	1.9	4.8	6.7	**1.0**^{ab}	0.5	0.0	7.1	0.0	0.0	5.2	3.3	2.4
		55+	1.9	4.8	12.9	**0.0**^{b}	1.9	0.0	6.7	0.0	0.0	6.7	2.4	3.8
		p-value	0.747	0.965	0.100	**0.029**	0.364	1.000	0.849	1.000	0.368	0.422	0.639	0.296
	Peru	18-34	1.4	**1.4**^{b}	**7.6**^{b}	**0.0**^{b}	**1.0**^{b}	0.0	1.9	0.0	0.0	**1.9**^{b}	2.4	1.0
		35-54	1.0	**4.3**^{ab}	**11.0**^{ab}	**1.4**^{ab}	**1.4**^{b}	0.5	5.2	0.0	0.0	**4.8**^{ab}	3.8	1.4
		55+	2.9	**8.1**^{a}	**15.2**^{a}	**2.9**^{a}	**5.2**^{a}	0.0	5.7	0.0	0.0	**7.6**^{a}	3.3	3.3
		p-value	0.301	**0.005**	**0.047**	**0.048**	**0.009**	0.368	0.111	1.000	1.000	**0.023**	0.697	0.168
	Thailand	18-34	**1.0**^{b}	0.0	**4.3**^{b}	1.4	**0.5**^{b}	0.0	4.8	0.0	0.0	3.3	0.5	1.4
		35-54	**4.8**^{a}	2.4	**11.4**^{a}	1.4	**1.4**^{b}	0.0	7.1	0.0	0.0	6.7	2.4	1.4
		55+	**5.7**^{a}	2.4	**11.0**^{a}	1.0	**4.3**^{a}	0.5	5.7	0.0	0.5	6.2	1.4	0.5
		p-value	**0.026**	0.079	**0.016**	0.881	**0.017**	0.368	0.580	1.000	0.368	0.263	0.259	0.562
D	Brazil	18-34	1.0	3.3	3.8	1.4	0.5	0.0	17.6	1.0	0.0	3.3	0.5	1.9
		35-54	3.8	2.4	4.8	3.3	1.0	0.5	18.6	0.0	0.0	3.3	1.9	4.8
		55+	2.9	2.4	6.7	5.2	0.0	0.0	17.6	0.0	0.0	2.4	1.0	2.9
		p-value	0.167	0.785	0.398	0.094	0.367	0.368	0.958	0.135	1.000	0.805	0.364	0.236
	India	18-34	0.9	0.0	0.9	1.4	0.0	0.0	4.3	0.0	0.9	0.9	0.0	0.9
		35-54	1.4	1.0	2.4	1.0	0.0	0.5	3.8	0.5	1.4	0.5	0.0	0.0
		55+	3.8	1.0	1.4	1.0	1.0	0.0	2.4	0.0	1.0	1.9	0.0	0.5
		p-value	0.087	0.364	0.488	0.869	0.134	0.367	0.547	0.367	0.864	0.363	1.000	0.369
	South Africa	18-34	1.4	1.4	1.9	1.4	1.0	0.0	7.6	0.5	0.5	1.4	0.0	2.4
		35-54	1.0	0.5	2.9	1.0	0.0	0.0	6.7	0.0	0.5	2.4	1.4	1.0
		55+	1.0	1.9	3.8	3.8	1.4	0.5	9.5	0.5	0.5	2.9	1.0	2.9
		p-value	0.866	0.413	0.504	0.088	0.244	0.368	0.545	0.606	1.000	0.600	0.244	0.361
			**Salty snack food**											
W	USA	18-34	**8.6**^{ab}	**21.5**^{a}	24.9	13.4	12.0	6.7	29.2	8.6	9.1	21.5	7.7	12.9
		35-54	**12.8**^{a}	**18.0**^{a}	24.6	18.0	15.6	5.2	23.2	8.1	9.0	22.7	13.7	13.3
		55+	**5.7**^{b}	**10.5**^{b}	18.1	12.9	9.5	1.9	20.5	4.3	4.8	20.5	10.5	8.1
		p-value	**0.039**	**0.008**	0.169	0.262	0.159	0.057	0.105	0.167	0.161	0.852	0.129	0.177

Cluster	Country	Age	Ant egg	Ant	Bee	Beetle	Caterpillar	Cockroach	Cricket	Fly larvae	Fly	Grasshopper	Mealworm	Wasp
X	Australia	18-34	1.4	2.4	4.8	3.3	1.4	0.0	6.7	0.0	0.0	5.7	2.4	2.9
		35-54	1.4	3.3	5.2	1.9	1.4	0.0	6.7	0.0	0.5	4.3	1.4	1.9
		55+	0.5	1.9	6.2	1.0	0.5	0.0	4.8	0.0	0.5	3.8	2.9	1.0
		p-value	0.562	0.639	0.805	0.225	0.562	1.000	0.639	1.000	0.606	0.626	0.600	0.361
	China	18-34	2.4	4.3	9.0	2.4	1.0	0.5	2.9	0.0	0.0	7.6	2.4	**0.5[b]**
		35-54	0.5	2.4	5.7	1.0	1.0	0.0	3.3	0.5	0.5	4.8	0.0	**0.5[b]**
		55+	1.0	5.7	8.1	1.9	0.5	0.5	2.9	0.0	1.0	8.1	1.0	**2.9[a]**
		p-value	0.193	0.227	0.416	0.524	0.818	0.606	0.947	0.368	0.367	0.342	0.065	**0.042**
	Japan	18-34	1.0	1.4	4.3	0.5	2.4	0.5	3.8	1.0	0.5	5.7	1.0	1.9
		35-54	0.5	1.9	8.6	0.5	1.0	0.0	1.4	0.0	0.0	4.3	0.5	1.0
		55+	1.9	1.9	6.2	0.0	1.0	0.0	1.9	0.5	0.0	9.5	0.0	1.4
		p-value	0.364	0.912	0.197	0.606	0.363	0.368	0.239	0.367	0.368	0.080	0.367	0.713
	Russia	18-34	1.4	4.3	2.4	**1.9[a]**	1.4	0.0	**2.9[b]**	0.0	0.5	5.2	1.0	0.5
		35-54	1.0	3.3	4.3	**0.0[b]**	1.4	0.0	**7.6[a]**	0.0	0.0	5.2	0.0	1.9
		55+	0.5	3.8	5.7	**0.0[b]**	0.0	0.0	**3.3[b]**	0.0	0.0	3.8	0.5	1.0
		p-value	0.604	0.878	0.227	**0.018**	0.220	1.000	**0.037**	1.000	0.368	0.730	0.367	0.364
	Spain	18-34	0.0	3.3	4.3	1.9	0.5	0.0	5.2	0.0	0.5	4.8	0.0	1.0
		35-54	1.0	6.2	7.6	1.9	2.4	0.0	4.8	0.0	0.5	2.9	0.5	1.0
		55+	1.4	1.9	5.7	1.0	1.4	0.0	2.4	1.0	0.5	3.8	0.5	1.0
		p-value	0.244	0.066	0.346	0.666	0.259	1.000	0.289	0.135	1.000	0.595	0.606	1.000
	UK	18-34	2.4	3.8	6.7	3.3	1.9	0.0	6.2	1.0	1.9	3.8	1.9	2.9
		35-54	1.4	2.4	4.8	3.8	1.9	0.0	3.3	0.5	0.5	5.7	1.4	1.4
		55+	0.0	1.0	2.4	1.4	0.5	0.5	3.3	0.5	0.5	3.8	0.5	1.0
		p-value	0.091	0.159	0.111	0.301	0.363	0.368	0.249	0.778	0.220	0.550	0.413	0.301
Y	Mexico	18-34	1.4	5.2	10.5	3.8	4.3	0.0	12.9	0.0	0.0	9.5	2.4	2.9
		35-54	3.3	6.2	9.5	2.4	1.9	0.0	10.5	0.0	0.0	9.5	4.8	3.3
		55+	3.8	8.6	16.7	0.5	3.8	0.0	12.4	0.0	0.0	14.3	3.3	2.9
		p-value	0.301	0.370	0.053	0.067	0.356	1.000	0.728	1.000	1.000	0.201	0.409	0.947
	Peru	18-34	1.0	4.3	**10.0[b]**	1.9	**0.0[b]**	0.0	6.2	0.0	0.0	3.8	2.4	1.4
		35-54	1.4	6.7	**11.9[b]**	0.5	**3.8[a]**	0.0	8.1	0.0	0.0	4.8	4.3	3.8
		55+	1.9	7.6	**20.0[a]**	3.3	**4.8[a]**	0.0	7.6	0.0	0.0	7.6	3.3	5.2
		p-value	0.713	0.345	**0.007**	0.101	**0.008**	1.000	0.738	1.000	1.000	0.199	0.554	0.100
	Thailand	18-34	2.4	1.4	6.7	1.0	1.4	0.0	9.0	0.0	0.0	7.6	0.0	1.9
		35-54	4.8	1.9	10.5	1.4	1.4	0.0	15.2	0.0	0.0	11.0	1.9	2.4
		55+	5.2	4.3	12.9	2.4	3.8	0.0	11.9	0.0	0.0	13.8	1.4	2.9

		p-value	0.289	0.137	0.103	0.492	0.161	1.000	0.150	1.000	1.000	0.124	0.153	0.815
Cluster	Country	Age	Ant egg	Ant	Bee	Beetle	Caterpillar	Cockroach	Cricket	Fly larvae	Fly	Grasshopper	Mealworm	Wasp
Z	Brazil	18-34	3.3	2.4	4.8	1.9	1.0	0.0	22.4	0.0	0.0	4.8	0.5	1.4
		35-54	4.8	2.4	3.8	3.8	1.0	0.5	19.0	0.0	0.5	3.8	1.4	3.8
		55+	4.3	4.3	6.7	2.9	0.0	0.0	18.6	0.0	0.0	4.3	0.0	5.2
		p-value	0.756	0.420	0.398	0.504	0.366	0.368	0.569	1.000	0.368	0.891	0.172	0.100
	India	18-34	2.4	1.4	1.4	2.4	0.0	0.5	6.2	0.5	1.4	2.4	0.9	1.4
		35-54	2.4	1.0	1.0	2.4	0.0	0.5	6.7	0.0	1.0	2.4	0.5	1.9
		55+	1.9	0.5	1.4	1.0	0.0	0.0	4.3	0.0	1.9	2.4	0.0	1.0
		p-value	0.931	0.607	0.883	0.468	1.000	0.607	0.541	0.370	0.713	1.000	0.369	0.713
	South Africa	18-34	1.0	1.9	1.9	1.9	1.4	0.0	13.3	0.0	0.5	**2.9**[b]	0.5	**1.0**[b]
		35-54	2.9	0.5	1.0	1.4	0.0	0.0	10.5	0.0	0.0	**2.4**[b]	0.5	**1.0**[b]
		55+	1.9	2.4	1.9	4.3	1.4	0.5	13.8	0.5	0.5	**7.6**[a]	1.4	**5.7**[a]
		p-value	0.361	0.267	0.666	0.137	0.220	0.368	0.537	0.368	0.606	**0.014**	0.447	**0.002**
			**Meat patty**											
W	USA	18-34	8.6	**16.7**[a]	15.8	14.4	14.4	**7.7**[a]	23.9	6.2	**12.0**[a]	19.1	9.1	10.0
		35-54	11.4	**16.6**[a]	23.7	14.2	15.2	**4.3**[ab]	21.8	8.5	**7.6**[ab]	20.4	15.2	11.4
		55+	6.2	**8.6**[b]	15.7	12.9	9.5	**2.4**[b]	19.5	5.2	**4.3**[b]	18.6	10.0	7.6
		p-value	0.170	**0.022**	0.053	0.886	0.178	**0.037**	0.552	0.381	**0.014**	0.891	0.108	0.418
X	Australia	18-34	1.0	2.4	6.7	2.9	2.9	0.0	6.2	0.0	0.0	5.7	3.8	2.4
		35-54	1.4	2.9	5.2	2.9	2.4	0.0	3.8	0.0	0.5	3.3	2.4	1.0
		55+	0.5	1.4	5.7	1.9	1.0	0.5	3.8	0.0	0.0	3.3	3.3	1.9
		p-value	0.604	0.600	0.818	0.774	0.361	0.368	0.406	1.000	0.368	0.367	0.697	0.524
	China	18-34	1.0	2.4	4.8	0.5	1.9	0.0	3.8	0.5	0.5	5.7	2.4	1.0
		35-54	0.5	2.4	6.7	1.0	0.5	0.0	3.8	0.0	0.0	3.8	1.4	1.0
		55+	1.0	4.3	9.5	1.9	1.4	0.0	3.3	0.0	0.5	4.3	1.0	1.0
		p-value	0.818	0.420	0.157	0.364	0.413	1.000	0.956	0.368	0.606	0.626	0.492	1.000
	Japan	18-34	2.4	1.4	**1.9**[b]	1.4	1.0	0.5	**3.8**[a]	1.0	1.0	3.8	1.4	2.4
		35-54	1.0	0.5	**8.1**[a]	1.9	1.0	0.0	**0.5**[b]	0.0	0.0	3.8	0.5	1.4
		55+	1.0	0.5	**3.8**[b]	0.0	0.5	0.0	**1.4**[ab]	0.5	0.0	6.7	0.0	1.4
		p-value	0.363	0.447	**0.008**	0.153	0.818	0.368	**0.037**	0.367	0.135	0.284	0.172	0.691
	Russia	18-34	0.0	2.4	2.4	1.9	1.9	0.0	3.3	0.0	0.0	2.4	0.0	1.0
		35-54	0.5	2.4	3.3	0.5	0.5	0.0	1.9	0.0	0.0	2.9	0.0	0.5
		55+	0.0	1.4	3.8	0.5	0.5	0.0	2.4	0.0	0.0	4.3	0.0	1.0
		p-value	0.368	0.731	0.697	0.220	0.220	1.000	0.639	1.000	1.000	0.512	1.000	0.818

Cluster	Country	Age	Ant egg	Ant	Bee	Beetle	Caterpillar	Cockroach	Cricket	Fly larvae	Fly	Grasshopper	Mealworm	Wasp
	Spain	18-34	1.0	4.8	3.3	2.4	1.4	0.0	3.8	0.0	0.0	3.3	0.5	0.5
		35-54	1.4	2.4	5.2	1.4	0.5	0.0	2.9	0.0	0.5	2.9	1.0	1.0
		55+	1.0	1.4	3.3	1.0	1.0	0.0	2.9	0.0	0.0	2.4	0.5	1.4
		p-value	0.866	0.108	0.514	0.492	0.604	1.000	0.814	1.000	0.368	0.843	0.778	0.604
	UK	18-34	1.0	3.3	5.2	1.4	2.4	0.0	3.8	1.4	2.4	3.3	1.9	2.4
		35-54	1.0	2.9	4.3	3.3	2.9	0.0	3.8	1.0	1.0	3.3	1.4	1.0
		55+	0.0	1.0	1.4	1.9	1.0	0.0	2.4	0.0	0.0	3.8	1.0	1.0
		p-value	0.366	0.239	0.096	0.387	0.361	1.000	0.642	0.244	0.065	0.954	0.713	0.363
Y	Mexico	18-34	2.4	4.8	**7.1**[ab]	1.9	1.4	0.0	12.4	0.0	0.0	9.5	1.4	2.4
		35-54	1.4	4.8	**4.3**[b]	1.0	1.4	0.0	7.6	0.0	0.0	8.1	3.3	2.9
		55+	3.8	5.2	**11.0**[a]	2.9	2.4	0.0	10.0	0.0	0.0	10.0	2.9	4.3
		p-value	0.296	0.967	**0.033**	0.361	0.691	1.000	0.267	1.000	1.000	0.782	0.435	0.512
	Peru	18-34	1.0	3.8	**6.7**[b]	0.0	**0.5**[b]	0.0	4.3	0.0	0.0	**2.4**[b]	2.9	**0.5**[b]
		35-54	1.0	4.8	**8.1**[b]	1.0	**5.2**[a]	0.0	6.7	0.0	0.0	**3.8**[b]	3.3	**2.4**[ab]
		55+	1.9	9.0	**17.1**[a]	2.4	**5.2**[a]	0.0	6.7	0.0	0.5	**8.1**[a]	5.2	**4.3**[a]
		p-value	0.603	0.052	**0.001**	0.065	**0.011**	1.000	0.488	1.000	0.368	**0.017**	0.403	**0.038**
	Thailand	18-34	1.4	1.0	**4.8**[b]	**1.0**[b]	0.5	0.0	**6.2**[b]	0.0	0.0	7.6	0.0	0.5
		35-54	5.2	1.4	**7.1**[b]	**1.4**[b]	2.9	0.0	**11.0**[ab]	0.0	0.0	8.6	2.9	2.4
		55+	5.2	3.8	**12.9**[a]	**4.8**[a]	3.8	0.0	**13.8**[a]	0.0	1.0	13.8	2.4	2.4
		p-value	0.070	0.088	**0.008**	**0.021**	0.070	1.000	**0.035**	1.000	0.135	0.075	0.057	0.228
Z	Brazil	18-34	2.4	1.4	2.9	1.4	1.4	0.0	20.0	0.0	0.5	2.9	0.5	1.4
		35-54	4.3	1.4	5.2	3.3	0.5	0.5	18.6	0.0	0.0	4.3	1.9	3.3
		55+	4.8	2.9	7.1	3.3	0.0	0.5	16.7	0.0	0.0	4.8	0.5	3.3
		p-value	0.403	0.466	0.135	0.381	0.172	0.606	0.677	1.000	0.368	0.582	0.220	0.381
	India	18-34	0.9	0.9	1.4	2.8	0.5	0.5	**8.1**[a]	0.0	0.9	2.4	1.4	1.9
		35-54	1.9	1.9	1.9	1.4	0.5	0.5	**8.6**[a]	0.0	1.9	1.9	0.0	1.4
		55+	1.9	1.4	1.4	0.5	0.0	0.5	**2.4**[b]	0.0	1.4	1.4	0.5	0.5
		p-value	0.663	0.710	0.902	0.148	0.607	1.000	**0.015**	1.000	0.710	0.779	0.174	0.416
	South Africa	18-34	1.9	1.4	1.9	2.9	0.5	0.5	11.9	0.5	0.5	2.4	0.5	0.5
		35-54	1.0	0.5	1.9	1.4	0.0	0.5	9.5	0.0	0.5	2.4	0.5	1.0
		55+	1.4	2.4	3.8	3.8	1.0	0.5	11.4	0.5	0.5	4.3	1.0	2.9
		p-value	0.713	0.259	0.359	0.318	0.367	1.000	0.711	0.606	1.000	0.420	0.778	0.094

[1]The Kruskal-Wallis test was performed to investigate whether the frequency of insect powder selection in each country differed significantly according the age.

[2]Bold highlights means that there is a significant difference (*p*-value < 0.05).

[3 a,b] letters performed multiple pairwise comparisons using Dunn's procedure, indicating significant differences between insect powders in each country (p-value < 0.05).

Table S3. Significant differences in the CATA frequency percentage indicating consumers' willingness to consume specific insect powder according to demographic (the highest degree earned; High school or less and college graduate) across the five food types arranged by country clusters.

Cluster	Country	Highest degree earned	Ant egg	Ant	Bee	Beetle	Caterpillar	Cockroach	Cricket	Fly larvae	Fly	Grasshopper	Mealworm	Wasp
			**Muffin or Bread**											
		High School or less	10.2	16.7	27.6	13.6	12.2	5.4	23.5	6.8	7.1	21.4	11.9	12.9
A	USA	College+	10.4	17.3	23.5	17.9	13.1	6.8	27.1	7.4	7.4	23.8	11.6	8.9
		p-value	0.931	0.843	0.246	0.146	0.750	0.467	0.299	0.757	0.887	0.477	0.908	0.107
		High School or less	0.8	2.4	5.6	2.4	1.2	0.0	7.2	0.0	0.4	5.6	2.4	0.8
B	Australia	College+	1.3	2.4	9.0	3.4	2.6	0.5	7.4	0.3	0.5	5.5	2.4	1.3
		p-value	0.541	0.991	0.116	0.456	0.213	0.250	0.919	0.418	0.819	0.985	0.991	0.541
		High School or less	0.9	1.8	7.2	**4.1**	2.7	0.9	3.6	1.4	0.5	6.3	0.5	2.3
	Japan	College+	1.0	1.0	7.1	**0.5**	1.2	0.5	2.0	0.5	0.2	5.9	0.7	1.0
		p-value	0.923	0.380	0.964	**0.001**	0.177	0.537	0.211	0.246	0.664	0.831	0.669	0.199
		High School or less	0.0	0.0	7.1	1.8	0.0	0.0	5.4	0.0	0.0	5.4	1.8	1.8
	Russia	College+	1.0	3.1	6.6	1.0	0.9	0.0	3.3	0.0	0.3	6.1	0.3	1.4
		p-value	0.444	0.180	0.882	0.616	0.485	1.000	0.427	1.000	0.661	0.825	0.137	0.815
		High School or less	1.0	3.0	5.7	1.0	0.7	0.0	3.7	0.0	0.0	3.4	1.0	1.0
	Spain	College+	1.5	3.9	8.4	1.5	0.9	0.6	3.3	0.0	0.3	3.3	0.9	1.2
		p-value	0.590	0.562	0.200	0.590	0.755	0.183	0.774	1.000	0.348	0.953	0.883	0.827
		High School or less	1.6	3.7	7.8	1.2	1.2	0.0	5.7	0.4	0.4	2.9	1.6	2.0
	UK	College+	1.3	2.9	5.7	3.1	1.3	0.5	5.7	1.0	1.0	5.5	2.1	1.8
		p-value	0.732	0.570	0.312	0.129	0.936	0.260	1.000	0.386	0.386	0.123	0.691	0.843
		High School or less	2.2	2.2	8.2	1.5	1.5	0.7	3.0	0.7	1.5	6.7	0.7	2.2
C	China	College+	1.0	4.0	14.3	0.8	1.2	0.6	3.6	0.2	0.2	6.5	3.2	1.6
		p-value	0.260	0.327	0.063	0.469	0.796	0.857	0.719	0.322	0.055	0.913	0.117	0.625
		High School or less	3.6	3.6	25.0	**7.1**	7.1	0.0	17.9	0.0	0.0	10.7	3.6	3.6
	Mexico	College+	3.8	8.0	15.6	**1.5**	2.2	0.2	14.0	0.0	0.0	11.8	3.5	3.5
		p-value	0.948	0.396	0.186	**0.026**	0.092	0.835	0.563	1.000	1.000	0.863	0.983	0.983
		High School or less	1.6	4.9	11.5	1.6	3.3	0.0	4.9	0.0	0.0	4.9	3.3	0.0
	Peru	College+	1.9	7.4	17.4	1.8	2.8	0.2	7.6	0.0	0.0	6.3	3.7	2.1
		p-value	0.875	0.479	0.240	0.948	0.836	0.747	0.452	1.000	1.000	0.665	0.871	0.253

Cluster	Country	Highest degree earned	Ant egg	Ant	Bee	Beetle	Caterpillar	Cockroach	Cricket	Fly larvae	Fly	Grasshopper	Mealworm	Wasp
		High School or less	4.4	2.2	10.0	1.1	1.1	0.0	18.9	0.0	0.0	10.0	2.2	1.1
	Thailand	College+	5.0	3.3	14.6	2.4	2.8	0.0	11.7	0.2	0.0	10.4	1.3	2.2
		p-value	0.822	0.579	0.241	0.441	0.353	1.000	0.057	0.686	1.000	0.915	0.494	0.494
		High School or less	4.2	2.7	4.2	2.3	0.8	0.4	32.4	0.0	0.0	5.0	0.4	2.7
D	Brazil	College+	5.4	4.0	5.9	2.2	0.5	0.3	35.3	0.5	0.3	4.3	1.9	3.5
		p-value	0.514	0.368	0.352	0.894	0.718	0.800	0.454	0.238	0.405	0.678	0.098	0.573
		High School or less	3.3	0.0	3.3	3.3	0.0	3.3	16.7	**6.7**	3.3	0.0	0.0	6.7
	India	College+	3.7	0.5	2.2	2.7	0.0	0.7	10.3	**0.8**	1.2	2.2	0.5	2.8
		p-value	0.927	0.702	0.673	0.826	1.000	0.109	0.271	**0.003**	0.302	0.417	0.702	0.231
		High School or less	3.0	0.9	3.0	2.6	0.9	0.0	17.3	0.4	0.4	2.6	0.9	2.2
	South Af rica	College+	2.8	1.5	2.0	3.5	1.8	0.3	23.6	0.3	0.3	4.0	1.5	3.3
		p-value	0.843	0.492	0.417	0.530	0.366	0.449	0.065	0.697	0.697	0.353	0.492	0.428
			**Cracker**											
		High School or less	9.2	15.3	21.4	13.6	12.2	5.1	22.8	5.1	9.2	19.4	11.9	11.9
A	USA	College+	9.5	13.4	19.9	13.4	13.1	4.8	26.5	7.4	8.3	22.0	11.9	11.0
		p-value	0.884	0.494	0.646	0.938	0.750	0.844	0.284	0.230	0.707	0.417	1.000	0.726
		High School or less	**0.0**	1.6	4.4	2.4	1.2	0.0	4.4	0.0	0.4	2.8	1.6	1.2
B	Australia	College+	**1.6**	1.8	5.0	1.6	1.6	0.3	5.8	0.0	0.3	4.7	1.6	2.4
		p-value	**0.045**	0.813	0.717	0.469	0.689	0.418	0.433	1.000	0.771	0.218	0.993	0.290
		High School or less	1.8	0.5	**7.7**	0.9	1.4	0.0	4.1	0.5	0.0	6.8	0.9	1.4
	Japan	College+	1.0	1.2	**3.9**	0.2	0.7	0.0	2.2	0.0	0.0	4.7	0.5	1.2
		p-value	0.380	0.340	**0.045**	0.255	0.448	1.000	0.184	0.176	1.000	0.266	0.537	0.894
		High School or less	0.0	0.0	5.4	0.0	0.0	0.0	7.1	0.0	0.0	3.6	**1.8**	0.0
	Russia	College+	0.3	2.1	3.8	1.4	0.7	0.0	3.1	0.0	0.2	3.7	**0.2**	1.0
		p-value	0.661	0.276	0.578	0.375	0.533	1.000	0.120	1.000	0.759	0.974	**0.041**	0.444
		High School or less	0.7	2.4	4.7	1.7	1.0	0.0	3.7	0.0	0.0	4.1	1.0	1.0
	Spain	College+	1.2	3.3	4.8	1.5	0.9	0.3	4.5	0.0	0.0	3.6	1.2	1.8
		p-value	0.502	0.486	0.972	0.848	0.883	0.348	0.626	1.000	1.000	0.763	0.827	0.410
		High School or less	0.4	1.2	4.5	1.2	0.4	0.4	4.5	0.8	1.2	1.2	0.8	2.0
	UK	College+	1.6	2.1	4.4	2.6	2.1	0.3	4.2	1.3	0.5	3.9	1.6	1.0
		p-value	0.180	0.426	0.965	0.238	0.086	0.749	0.841	0.575	0.332	0.050	0.418	0.302
		High School or less	1.5	3.7	9.0	0.7	**3.0**	0.0	2.2	0.7	**2.2**	3.0	0.7	1.5
C	China	College+	1.0	4.4	7.5	1.0	**0.6**	0.0	4.4	0.0	**0.2**	5.6	1.2	1.8
		p-value	0.636	0.722	0.567	0.783	**0.020**	1.000	0.248	0.055	**0.009**	0.214	0.651	0.802

Cluster	Country	Highest degree earned	Ant egg	Ant	Bee	Beetle	Caterpillar	Cockroach	Cricket	Fly larvae	Fly	Grasshopper	Mealworm	Wasp
		High School or less	3.6	7.1	14.3	3.6	**7.1**	0.0	14.3	0.0	0.0	10.7	7.1	**10.7**
	Mexico	College+	2.7	4.8	10.3	1.2	**1.3**	0.0	11.6	0.2	0.0	9.3	2.3	**3.0**
		p-value	0.772	0.579	0.502	0.267	**0.016**	1.000	0.670	0.835	1.000	0.803	0.114	**0.026**
		High School or less	1.6	4.9	9.8	3.3	3.3	0.0	4.9	0.0	0.0	4.9	4.9	1.6
	Peru	College+	1.1	5.4	12.0	1.4	3.2	0.0	7.6	0.2	0.0	5.6	3.7	2.3
		p-value	0.681	0.863	0.627	0.267	0.962	1.000	0.452	0.747	1.000	0.820	0.635	0.746
		High School or less	2.2	1.1	6.7	1.1	0.0	**1.1**	10.0	0.0	0.0	3.3	1.1	0.0
	Thailand	College+	4.1	2.6	10.6	2.2	2.0	**0.0**	8.9	0.2	0.2	8.7	1.5	1.5
		p-value	0.396	0.394	0.256	0.494	0.173	**0.014**	0.734	0.686	0.686	0.081	0.785	0.246
		High School or less	3.1	2.3	4.6	4.2	0.4	0.8	15.8	0.0	0.4	3.9	0.8	1.5
D	Brazil	College+	2.7	2.4	4.9	2.4	0.0	0.0	20.8	0.3	1.1	3.2	1.3	2.7
		p-value	0.771	0.930	0.900	0.200	0.233	0.091	0.119	0.405	0.337	0.674	0.499	0.336
		High School or less	0.0	0.0	0.0	0.0	0.0	0.0	10.0	0.0	0.0	0.0	0.0	0.0
	India	College+	2.3	1.7	2.0	1.5	0.2	0.3	7.5	0.2	0.8	1.3	0.5	1.5
		p-value	0.399	0.478	0.436	0.502	0.829	0.756	0.614	0.829	0.619	0.527	0.702	0.502
	South Africa	High School or less	1.7	0.9	2.2	2.6	0.4	0.0	**9.5**	0.4	0.4	2.6	0.4	2.6
		College+	1.3	2.0	2.0	3.0	1.3	0.5	**15.5**	0.3	0.5	4.0	1.0	3.0
		p-value	0.627	0.271	0.893	0.767	0.308	0.283	**0.033**	0.697	0.906	0.353	0.439	0.767
			**Cake**											
		High School or less	9.5	13.3	19.0	13.3	12.9	4.1	19.7	4.1	8.2	17.3	10.9	9.2
A	USA	College+	9.2	13.4	20.2	13.7	11.6	4.8	21.7	7.4	9.8	18.8	11.6	11.0
		p-value	0.899	0.963	0.708	0.877	0.615	0.680	0.538	0.074	0.470	0.649	0.775	0.449
		High School or less	0.8	1.6	4.4	2.4	1.2	0.0	3.6	0.0	0.8	3.6	1.6	0.8
B	Australia	College+	1.6	2.6	5.8	1.1	1.8	0.0	4.2	0.0	0.5	4.2	2.1	1.6
		p-value	0.389	0.385	0.433	0.190	0.523	1.000	0.690	1.000	0.679	0.690	0.643	0.389
		High School or less	1.4	0.9	5.9	1.4	1.8	0.9	1.4	0.5	0.0	5.0	1.4	1.8
	Japan	College+	0.2	1.0	3.7	0.2	0.5	0.0	1.2	0.0	0.2	3.7	0.2	1.0
		p-value	0.096	0.923	0.205	0.096	0.106	0.055	0.894	0.176	0.463	0.442	0.096	0.380
		High School or less	0.0	1.8	1.8	0.0	0.0	0.0	3.6	0.0	0.0	1.8	0.0	0.0
	Russia	College+	0.7	1.7	4.4	0.5	0.0	0.0	1.6	0.0	0.2	1.2	0.2	1.0
		p-value	0.533	0.982	0.357	0.590	1.000	1.000	0.276	1.000	0.759	0.720	0.759	0.444
		High School or less	1.0	1.4	3.7	1.4	1.4	0.0	2.0	0.7	0.3	3.7	0.7	1.4
	Spain	College+	1.2	3.0	5.4	1.5	0.6	0.3	2.7	0.0	0.0	2.7	1.2	1.2
		p-value	0.827	0.163	0.318	0.879	0.333	0.348	0.584	0.133	0.290	0.466	0.502	0.864

Cluster	Country	Highest degree earned	Ant egg	Ant	Bee	Beetle	Caterpillar	Cockroach	Cricket	Fly larvae	Fly	Grasshopper	Mealworm	Wasp
	UK	High School or less	0.4	2.0	**1.2**	2.0	0.4	0.0	1.6	0.8	0.4	2.4	0.8	0.8
		College+	1.0	2.1	**4.4**	2.3	1.6	0.5	4.4	1.0	1.3	3.1	1.3	1.0
		p-value	0.386	0.975	**0.026**	0.806	0.180	0.260	0.058	0.780	0.263	0.625	0.575	0.780
C	China	High School or less	1.5	3.7	5.2	0.0	1.5	0.0	2.2	0.0	**2.2**	3.0	2.2	1.5
		College+	1.2	2.8	6.0	0.6	1.0	0.2	1.8	0.0	**0.2**	5.0	1.2	1.6
		p-value	0.796	0.586	0.719	0.368	0.636	0.606	0.751	1.000	**0.009**	0.314	0.374	0.922
	Mexico	High School or less	7.1	10.7	17.9	3.6	**7.1**	0.0	7.1	0.0	0.0	10.7	3.6	**14.3**
		College+	2.0	4.3	9.3	1.2	**0.8**	0.0	7.3	0.0	0.2	5.0	2.5	**2.0**
		p-value	0.071	0.115	0.135	0.267	**0.002**	1.000	0.975	1.000	0.835	0.184	0.724	**<0.0001**
	Peru	High School or less	1.6	1.6	9.8	1.6	1.6	**1.6**	1.6	0.0	0.0	3.3	1.6	0.0
		College+	1.8	4.9	11.4	1.4	2.6	**0.0**	4.6	0.0	0.0	4.9	3.3	2.1
		p-value	0.948	0.246	0.710	0.885	0.639	**0.002**	0.284	1.000	1.000	0.568	0.473	0.253
	Thailand	High School or less	1.1	1.1	7.8	1.1	1.1	0.0	3.3	0.0	**1.1**	2.2	0.0	0.0
		College+	4.3	1.7	9.1	1.3	2.2	0.2	6.3	0.0	**0.0**	5.9	1.7	1.3
		p-value	0.149	0.698	0.690	0.886	0.494	0.686	0.269	1.000	**0.014**	0.151	0.218	0.279
D	Brazil	High School or less	2.3	2.7	5.0	2.7	0.4	0.4	16.2	0.4	0.0	2.7	0.8	**1.2**
		College+	2.7	2.7	5.1	3.8	0.5	0.0	19.1	0.3	0.0	3.2	1.3	**4.6**
		p-value	0.767	0.996	0.955	0.462	0.785	0.233	0.348	0.800	1.000	0.702	0.499	**0.016**
	India	High School or less	0.0	0.0	3.3	3.3	0.0	0.0	6.7	**3.3**	0.0	0.0	0.0	0.0
		College+	2.2	0.7	1.5	1.0	0.3	0.2	3.3	**0.0**	1.2	1.2	0.0	0.5
		p-value	0.417	0.657	0.434	0.235	0.756	0.829	0.332	**<0.0001**	0.554	0.554	1.000	0.702
	South Africa	High School or less	0.9	1.3	2.2	2.6	0.4	0.0	5.2	0.4	0.4	2.6	0.9	2.2
		College+	1.3	1.3	3.3	1.8	1.0	0.3	9.5	0.3	0.5	2.0	0.8	2.0
		p-value	0.656	0.962	0.428	0.474	0.439	0.449	0.053	0.697	0.906	0.628	0.878	0.893
			**Salty snack food**											
W	USA	High School or less	8.8	15.0	24.5	15.6	13.6	3.7	23.1	5.8	7.5	21.8	10.2	12.9
		College+	9.2	18.2	20.8	14.0	11.3	5.4	25.3	8.0	7.7	21.4	11.0	10.1
		p-value	0.868	0.285	0.274	0.559	0.383	0.335	0.527	0.269	0.905	0.918	0.743	0.270
X	Australia	High School or less	**0.0**	1.2	4.4	2.0	0.8	0.0	6.0	0.0	0.4	4.4	2.0	1.6
		College+	**1.8**	3.4	6.1	2.1	1.3	0.0	6.1	0.0	0.3	4.7	2.4	2.1
		p-value	**0.031**	0.081	0.360	0.919	0.541	1.000	0.962	1.000	0.771	0.830	0.751	0.643
	China	High School or less	2.2	6.7	5.2	1.5	1.5	0.7	2.2	0.0	0.7	6.7	0.0	**3.0**
		College+	1.0	3.4	8.3	1.8	0.6	0.2	3.2	0.2	0.4	6.9	1.4	**0.8**
		p-value	0.260	0.090	0.239	0.802	0.305	0.322	0.554	0.606	0.611	0.956	0.167	**0.046**

Cluster	Country	Highest degree earned	Ant egg	Ant	Bee	Beetle	Caterpillar	Cockroach	Cricket	Fly larvae	Fly	Grasshopper	Mealworm	Wasp
		High School or less	0.5	**3.2**	8.1	0.5	2.3	0.0	3.6	0.9	0.0	8.6	0.5	2.3
	Japan	College+	1.5	**1.0**	5.4	0.2	1.0	0.2	1.7	0.2	0.2	5.4	0.5	1.0
		p-value	0.244	**0.047**	0.182	0.664	0.199	0.463	0.138	0.255	0.463	0.124	0.946	0.199
		High School or less	0.0	3.6	5.4	0.0	0.0	0.0	7.1	0.0	0.0	5.4	0.0	0.0
	Russia	College+	1.0	3.8	4.0	0.7	1.0	0.0	4.4	0.0	0.2	4.7	0.5	1.2
		p-value	0.444	0.923	0.629	0.533	0.444	1.000	0.343	1.000	0.759	0.827	0.590	0.408
		High School or less	0.3	3.7	5.1	1.0	1.4	0.0	4.1	0.0	0.3	3.0	0.3	1.4
	Spain	College+	1.2	3.9	6.6	2.1	1.5	0.0	4.2	0.6	0.6	4.5	0.3	0.6
		p-value	0.226	0.909	0.419	0.279	0.879	1.000	0.932	0.183	0.636	0.343	0.934	0.333
		High School or less	0.8	2.0	3.7	3.3	1.6	0.4	4.5	0.4	1.2	4.5	1.2	1.2
	UK	College+	1.6	2.6	5.2	2.6	1.3	0.0	4.2	0.8	0.8	4.4	1.3	2.1
		p-value	0.418	0.656	0.375	0.625	0.732	0.211	0.841	0.569	0.576	0.965	0.936	0.426
		High School or less	3.6	7.1	**25.0**	3.6	**10.7**	0.0	10.7	0.0	0.0	7.1	3.6	7.1
Y	Mexico	College+	2.8	6.6	**11.6**	2.2	**3.0**	0.0	12.0	0.0	0.0	11.3	3.5	2.8
		p-value	0.818	0.919	**0.035**	0.622	**0.026**	1.000	0.843	1.000	1.000	0.495	0.983	0.192
		High School or less	1.6	3.3	11.5	1.6	1.6	0.0	4.9	0.0	0.0	4.9	3.3	1.6
	Peru	College+	1.4	6.5	14.2	1.9	3.0	0.0	7.6	0.0	0.0	5.4	3.3	3.7
		p-value	0.885	0.322	0.555	0.875	0.549	1.000	0.452	1.000	1.000	0.863	0.981	0.408
		High School or less	2.2	3.3	5.6	1.1	2.2	0.0	15.6	0.0	0.0	11.1	1.1	1.1
	Thailand	College+	4.4	2.4	10.7	1.7	2.2	0.0	11.5	0.0	0.0	10.7	1.1	2.6
		p-value	0.327	0.606	0.129	0.698	1.000	1.000	0.273	1.000	1.000	0.917	1.000	0.394
		High School or less	4.2	3.1	4.6	2.7	0.8	0.4	**15.4**	0.0	0.0	4.6	0.4	2.7
Z	Brazil	College+	4.0	3.0	5.4	3.0	0.5	0.0	**23.2**	0.0	0.3	4.0	0.8	4.0
		p-value	0.900	0.929	0.671	0.847	0.718	0.233	**0.017**	1.000	0.405	0.720	0.513	0.368
		High School or less	3.3	0.0	0.0	3.3	0.0	0.0	3.3	0.0	3.3	6.7	**3.3**	0.0
	India	College+	2.2	1.0	1.3	1.8	0.0	0.3	5.8	0.2	1.3	2.2	**0.3**	1.5
		p-value	0.673	0.585	0.527	0.558	1.000	0.756	0.567	0.829	0.368	0.115	**0.020**	0.502
		High School or less	1.7	1.3	1.3	3.5	0.4	0.0	**9.1**	0.4	0.4	3.0	0.9	1.7
	South Africa	College+	2.0	1.8	1.8	2.0	1.3	0.3	**14.5**	0.0	0.3	5.0	0.8	3.0
		p-value	0.810	0.660	0.660	0.263	0.308	0.449	**0.047**	0.190	0.697	0.237	0.878	0.327
			**Meat patty**											
		High School or less	7.5	15.0	20.4	14.6	13.3	5.4	19.7	5.1	6.5	18.7	11.2	11.6
W	USA	College+	9.8	13.1	16.7	13.1	12.8	4.2	23.5	8.0	9.2	19.9	11.6	8.0
		p-value	0.300	0.500	0.227	0.579	0.862	0.454	0.251	0.141	0.201	0.696	0.881	0.136

Cluster	Country	Highest degree earned	Ant egg	Ant	Bee	Beetle	Caterpillar	Cockroach	Cricket	Fly larvae	Fly	Grasshopper	Mealworm	Wasp
		High School or less	**0.0**	1.6	4.4	2.4	2.0	0.0	3.6	0.0	0.4	3.6	2.0	1.2
X	Australia	College+	**1.6**	2.6	6.9	2.6	2.1	0.3	5.3	0.0	0.0	4.5	4.0	2.1
		p-value	**0.045**	0.385	0.196	0.847	0.919	0.418	0.322	1.000	0.220	0.579	0.169	0.391
		High School or less	0.7	3.7	5.2	**3.7**	0.7	0.0	2.2	0.7	0.7	4.5	1.5	0.7
	China	College+	0.8	2.8	7.5	**0.4**	1.4	0.0	4.0	0.0	0.2	4.6	1.6	1.0
		p-value	0.946	0.586	0.368	**0.001**	0.543	1.000	0.327	0.055	0.322	0.938	0.922	0.783
		High School or less	2.3	0.5	5.4	1.4	0.9	0.5	3.2	0.5	0.9	5.9	0.9	2.3
	Japan	College+	1.0	1.0	4.2	1.0	0.7	0.0	1.2	0.5	0.0	4.2	0.5	1.5
		p-value	0.199	0.475	0.479	0.672	0.824	0.176	0.091	0.946	0.055	0.342	0.537	0.475
		High School or less	0.0	0.0	1.8	1.8	0.0	0.0	5.4	0.0	0.0	1.8	0.0	0.0
	Russia	College+	0.2	2.3	3.3	0.9	1.0	0.0	2.3	0.0	0.0	3.3	0.0	0.9
		p-value	0.759	0.256	0.536	0.503	0.444	1.000	0.161	1.000	1.000	0.536	1.000	0.485
		High School or less	1.0	1.7	4.4	1.7	0.3	0.0	3.4	0.0	0.3	3.0	0.7	1.4
	Spain	College+	1.2	3.9	3.6	1.5	1.5	0.0	3.0	0.0	0.0	2.7	0.6	0.6
		p-value	0.827	0.098	0.609	0.848	0.136	1.000	0.784	1.000	0.290	0.796	0.905	0.333
		High School or less	0.0	2.4	3.3	2.0	1.6	0.0	2.9	0.8	0.4	3.3	0.8	2.0
	UK	College+	1.0	2.3	3.9	2.3	2.3	0.0	3.6	0.8	1.6	3.6	1.8	1.0
		p-value	0.110	0.930	0.681	0.806	0.545	1.000	0.596	0.960	0.180	0.805	0.302	0.302
		High School or less	7.1	7.1	10.7	0.0	**7.1**	0.0	3.6	0.0	0.0	7.1	7.1	7.1
Y	Mexico	College+	2.3	4.8	7.3	2.0	**1.5**	0.0	10.3	0.0	0.0	9.3	2.3	3.0
		p-value	0.114	0.579	0.504	0.452	**0.026**	1.000	0.247	1.000	1.000	0.700	0.114	0.222
		High School or less	1.6	1.6	8.2	1.6	1.6	0.0	3.3	0.0	0.0	3.3	1.6	0.0
	Peru	College+	1.2	6.3	10.9	1.1	3.9	0.0	6.2	0.0	0.2	4.9	4.0	2.6
		p-value	0.788	0.139	0.516	0.681	0.379	1.000	0.365	1.000	0.747	0.568	0.352	0.200
		High School or less	2.2	1.1	6.7	2.2	2.2	0.0	10.0	0.0	1.1	6.7	1.1	1.1
	Thailand	College+	4.3	2.2	8.5	2.4	2.4	0.0	10.4	0.0	0.2	10.6	1.9	1.9
		p-value	0.360	0.494	0.555	0.916	0.916	1.000	0.915	1.000	0.149	0.256	0.621	0.621
		High School or less	4.2	1.5	4.2	3.1	0.8	0.4	16.6	0.0	0.0	3.9	0.8	1.5
Z	Brazil	College+	3.5	2.2	5.7	2.4	0.5	0.3	19.7	0.0	0.3	4.0	1.1	3.5
		p-value	0.632	0.581	0.427	0.614	0.718	0.800	0.328	1.000	0.405	0.909	0.698	0.136
		High School or less	0.0	0.0	0.0	**6.7**	**3.3**	0.0	6.7	0.0	0.0	0.0	0.0	0.0
	India	College+	1.7	1.5	1.7	**1.3**	**0.2**	0.5	6.3	0.0	1.5	2.0	0.7	1.3
		p-value	0.478	0.502	0.478	**0.023**	**0.003**	0.702	0.941	1.000	0.502	0.436	0.657	0.527
	South	High School or less	1.3	1.7	2.2	3.9	0.4	0.4	8.2	0.4	0.4	3.5	0.4	1.3
	Africa	College+	1.5	1.3	2.8	2.0	0.5	0.5	12.5	0.3	0.5	2.8	0.8	1.5

		p-value	0.835	0.627	0.650	0.159	0.906	0.906	0.096	0.697	0.906	0.618	0.629	0.835

[1]The Mann-Whitney U test was performed to investigate whether the frequency of insect powder selection in each country differed significantly according to the level of education.

[2]Bold highlights means that there is a significant difference (p-value < 0.05).

Table S4. Significant differences in the CATA frequency percentage indicating consumers' willingness to consume specific insect powder according to demographic (number of adults aged 18 or order in household; 1-2, and 3+) across the five food types arranged by country clusters.

Cluster	Country	Number of adults ≥ 18	Ant egg	Ant	Bee	Beetle	Caterpillar	Cockroach	Cricket	Fly larvae	Fly	Grasshopper	Mealworm	Wasp
			**Muffin or Bread**											
		1-2	11.1	17.5	25.8	16.2	12.4	6.6	26.2	8.1	7.9	23.2	**13.4**	10.4
A	USA	3+	8.1	15.5	24.2	14.9	13.7	5.0	23.0	4.3	5.6	21.1	**6.8**	11.8
		p-value	0.279	0.569	0.692	0.698	0.670	0.457	0.415	0.111	0.334	0.580	**0.025**	0.634
		1-2	1.0	2.5	6.7	3.3	1.9	0.4	7.1	0.0	0.4	5.4	2.5	1.0
B	Australia	3+	1.3	2.0	10.5	2.0	2.6	0.0	7.9	0.7	0.7	5.9	2.0	1.3
		p-value	0.784	0.706	0.121	0.389	0.573	0.426	0.748	0.077	0.711	0.822	0.706	0.784
		1-2	0.6	1.2	6.3	0.9	1.2	0.6	2.7	0.9	0.3	4.8	0.3	1.5
	Japan	3+	1.4	1.4	8.1	2.7	2.4	0.7	2.4	0.7	0.3	7.4	1.0	1.4
		p-value	0.333	0.864	0.376	0.085	0.265	0.905	0.794	0.755	0.934	0.165	0.261	0.879
		1-2	1.2	2.5	5.7	1.0	0.7	0.0	3.0	0.0	0.2	5.5	0.5	1.5
	Russia	3+	0.4	3.5	8.3	1.3	0.9	0.0	4.4	0.0	0.4	7.0	0.4	1.3
		p-value	0.315	0.470	0.216	0.720	0.866	1.000	0.367	1.000	0.690	0.447	0.915	0.851
		1-2	0.0	0.0	7.9	0.0	0.0	0.0	1.6	0.0	0.0	1.6	0.0	3.2
	Spain	3+	1.4	3.9	7.1	1.4	0.9	0.4	3.7	0.0	0.2	3.5	1.1	0.9
		p-value	0.344	0.112	0.797	0.344	0.456	0.640	0.386	1.000	0.743	0.417	0.414	0.100
		1-2	1.9	3.4	7.2	2.1	1.3	0.2	5.7	0.8	0.8	4.6	2.1	1.9
	UK	3+	0.0	2.6	4.5	3.2	1.3	0.6	5.8	0.6	0.6	3.9	1.3	1.9
		p-value	0.085	0.628	0.248	0.428	0.980	0.405	0.955	0.812	0.812	0.691	0.520	0.975
		1-2	1.4	4.2	11.9	0.0	0.0	0.0	4.2	0.0	0.7	4.9	0.7	1.4
C	China	3+	1.2	3.5	13.3	1.2	1.6	0.8	3.3	0.4	0.4	7.0	3.3	1.8
		p-value	0.877	0.693	0.649	0.183	0.124	0.278	0.603	0.445	0.661	0.375	0.094	0.719
		1-2	5.1	7.7	25.6	0.0	0.0	0.0	15.4	0.0	0.0	17.9	5.1	5.1
	Mexico	3+	3.7	7.8	15.4	1.9	2.5	0.2	14.0	0.0	0.0	11.3	3.4	3.4
		p-value	0.658	0.984	0.092	0.392	0.315	0.802	0.817	1.000	1.000	0.215	0.567	0.567

Cluster	Country	Number of adults ≥ 18	Ant egg	Ant	Bee	Beetle	Caterpillar	Cockroach	Cricket	Fly larvae	Fly	Grasshopper	Mealworm	Wasp
		1-2	0.0	7.7	19.2	0.0	3.8	0.0	3.8	0.0	0.0	0.0	0.0	0.0
	Peru	3+	2.0	7.1	16.7	1.8	2.8	0.2	7.5	0.0	0.0	6.5	3.8	2.0
		p-value	0.470	0.913	0.739	0.489	0.759	0.842	0.490	1.000	1.000	0.182	0.312	0.470
		1-2	3.7	2.6	12.0	1.0	3.1	0.0	9.4	0.5	0.0	7.9	**0.0**	1.6
	Thailand	3+	5.5	3.4	14.8	2.7	2.3	0.0	14.1	0.0	0.0	11.4	**2.1**	2.3
		p-value	0.337	0.600	0.358	0.188	0.528	1.000	0.104	0.130	1.000	0.180	**0.047**	0.567
		1-2	5.5	3.7	5.2	3.0	0.6	0.3	37.5	0.3	0.3	4.9	1.5	3.4
D	Brazil	3+	4.3	3.3	5.3	1.3	0.7	0.3	30.5	0.3	0.0	4.3	1.0	3.0
		p-value	0.493	0.813	0.949	0.143	0.935	0.955	0.063	0.955	0.339	0.732	0.553	0.790
		1-2	1.7	0.6	3.5	1.2	0.0	0.6	7.5	1.2	0.0	2.3	0.0	1.2
	India	3+	4.4	0.4	1.7	3.3	0.0	0.9	11.8	1.1	1.7	2.0	0.7	3.7
		p-value	0.116	0.820	0.191	0.143	1.000	0.710	0.120	0.946	0.081	0.785	0.287	0.094
		1-2	3.3	1.6	1.6	2.7	1.4	0.0	21.1	0.0	0.3	3.0	1.6	3.3
	South Africa	3+	2.3	0.8	3.4	3.8	1.5	0.4	21.5	0.8	0.4	4.2	0.8	2.3
		p-value	0.447	0.326	0.155	0.466	0.885	0.242	0.901	0.097	0.822	0.444	0.326	0.447
			**Cracker**											
		1-2	10.7	15.4	20.9	14.5	13.0	4.9	24.9	7.0	8.3	20.0	**13.4**	11.3
A	USA	3+	5.6	11.2	19.9	10.6	11.8	5.0	24.2	4.3	9.9	23.0	**7.5**	11.8
		p-value	0.057	0.192	0.783	0.207	0.692	0.974	0.855	0.228	0.530	0.428	**0.043**	0.864
		1-2	0.6	1.5	5.2	2.1	1.3	0.0	5.2	0.0	0.2	3.8	1.3	1.5
B	Australia	3+	2.0	2.6	3.3	1.3	2.0	0.7	5.3	0.0	0.7	4.6	2.6	3.3
		p-value	0.137	0.340	0.328	0.543	0.517	0.077	0.988	1.000	0.394	0.645	0.238	0.152
		1-2	0.6	1.2	**3.3**	0.0	0.3	0.0	2.7	0.3	0.0	4.5	0.3	0.9
	Japan	3+	2.0	0.7	**7.4**	1.0	1.7	0.0	3.0	0.0	0.0	6.4	1.0	1.7
		p-value	0.111	0.502	**0.020**	0.066	0.073	1.000	0.796	0.348	1.000	0.286	0.261	0.377
		1-2	0.2	2.0	3.5	1.5	0.5	0.0	3.5	0.0	0.0	4.5	0.5	0.7
	Russia	3+	0.4	1.7	4.8	0.9	0.9	0.0	3.5	0.0	0.4	2.2	0.0	1.3
		p-value	0.690	0.827	0.418	0.503	0.571	1.000	0.999	1.000	0.187	0.138	0.286	0.486
		1-2	1.6	3.2	4.8	0.0	0.0	0.0	0.0	0.0	0.0	0.0	1.6	3.2
	Spain	3+	0.9	2.8	4.8	1.8	1.1	0.2	4.6	0.0	0.0	4.2	1.1	1.2
		p-value	0.586	0.874	1.000	0.289	0.414	0.743	0.083	1.000	1.000	0.096	0.706	0.219
		1-2	1.5	2.1	4.8	2.5	1.7	0.2	4.2	1.1	1.1	3.4	1.1	1.5
	UK	3+	0.0	0.6	3.2	0.6	0.6	0.6	4.5	1.3	0.0	1.3	1.9	1.3
		p-value	0.129	0.229	0.397	0.153	0.345	0.405	0.871	0.808	0.201	0.178	0.395	0.868

Cluster	Country	Number of adults ≥ 18	Ant egg	Ant	Bee	Beetle	Caterpillar	Cockroach	Cricket	Fly larvae	Fly	Grasshopper	Mealworm	Wasp
		1-2	0.0	3.5	9.1	0.0	0.0	0.0	2.8	0.0	0.7	3.5	0.7	2.1
C	China	3+	1.4	4.5	7.4	1.2	1.4	0.0	4.3	0.2	0.6	5.5	1.2	1.6
		p-value	0.150	0.597	0.506	0.183	0.150	1.000	0.415	0.591	0.914	0.328	0.594	0.716
		1-2	5.1	2.6	**23.1**	0.0	0.0	0.0	12.8	0.0	0.0	17.9	5.1	7.7
	Mexico	3+	2.5	5.1	**9.6**	1.4	1.7	0.0	11.7	0.2	0.0	8.8	2.4	3.0
		p-value	0.335	0.483	**0.008**	0.466	0.414	1.000	0.830	0.802	1.000	0.058	0.290	0.118
		1-2	0.0	3.8	15.4	0.0	0.0	0.0	0.0	0.0	0.0	0.0	0.0	7.7
	Peru	3+	1.2	5.5	11.6	1.7	3.3	0.0	7.6	0.2	0.0	5.8	4.0	2.0
		p-value	0.583	0.722	0.557	0.510	0.347	1.000	0.145	0.842	1.000	0.207	0.301	0.054
		1-2	2.1	1.6	**4.7**	1.6	0.5	0.0	5.8	0.5	0.0	**4.7**	1.0	0.5
	Thailand	3+	4.6	2.7	**12.3**	2.3	2.3	0.2	10.5	0.0	0.2	**9.3**	1.6	1.6
		p-value	0.138	0.380	**0.004**	0.567	0.123	0.512	0.058	0.130	0.512	**0.049**	0.596	0.271
		1-2	**4.3**	2.4	4.6	4.3	0.3	0.6	20.7	0.0	0.3	4.0	1.2	2.1
D	Brazil	3+	**1.3**	2.3	5.0	2.0	0.0	0.0	16.6	0.3	1.3	3.0	1.0	2.3
		p-value	**0.027**	0.921	0.817	0.103	0.339	0.175	0.180	0.299	0.150	0.503	0.788	0.877
		1-2	2.3	1.7	2.9	0.6	0.6	0.0	5.8	0.0	0.0	1.7	0.0	1.2
	India	3+	2.2	1.5	1.5	1.7	0.0	0.4	8.3	0.2	1.1	1.1	0.7	1.5
		p-value	0.923	0.855	0.265	0.270	0.104	0.386	0.288	0.541	0.168	0.521	0.287	0.726
		1-2	1.6	1.4	1.9	2.2	0.8	0.0	14.0	0.3	0.3	3.8	0.8	2.7
	South Africa	3+	1.1	1.9	2.3	3.8	1.1	0.8	12.5	0.4	0.8	3.0	0.8	3.0
		p-value	0.594	0.609	0.764	0.240	0.694	0.097	0.580	0.822	0.388	0.582	0.926	0.836
			**Cake**											
		1-2	10.4	14.3	20.0	13.6	13.6	4.9	21.5	**7.2**	9.6	18.6	12.2	10.2
A	USA	3+	6.2	10.6	18.6	13.0	8.1	3.1	18.6	**1.9**	7.5	16.8	8.7	9.9
		p-value	0.112	0.231	0.699	0.847	0.063	0.340	0.434	**0.012**	0.414	0.613	0.232	0.915
		1-2	0.8	2.1	5.0	1.5	1.0	0.0	3.6	0.0	0.6	4.0	1.5	0.8
B	Australia	3+	2.6	2.6	5.9	2.0	3.3	0.0	5.3	0.0	0.7	3.9	3.3	2.6
		p-value	0.086	0.695	0.665	0.663	0.054	1.000	0.349	1.000	0.969	0.989	0.152	0.086
		1-2	0.3	0.9	3.6	**0.0**	0.6	0.0	1.8	0.0	0.0	3.0	0.6	1.5
	Japan	3+	1.0	1.0	5.4	**1.4**	1.4	0.7	0.7	0.3	0.3	5.4	0.7	1.0
		p-value	0.261	0.883	0.271	**0.033**	0.333	0.133	0.211	0.290	0.290	0.129	0.905	0.590
		1-2	0.5	1.5	3.2	0.7	0.0	0.0	1.5	0.0	0.2	0.7	0.0	0.7
	Russia	3+	0.9	2.2	5.7	0.0	0.0	0.0	2.2	0.0	0.0	2.2	0.4	1.3
		p-value	0.571	0.527	0.140	0.191	1.000	1.000	0.527	1.000	0.452	0.122	0.187	0.486

Cluster	Country	Number of adults ≥ 18	Ant egg	Ant	Bee	Beetle	Caterpillar	Cockroach	Cricket	Fly larvae	Fly	Grasshopper	Mealworm	Wasp
		1-2	1.6	1.6	6.3	0.0	1.6	**1.6**	0.0	0.0	**1.6**	1.6	1.6	0.0
	Spain	3+	1.1	2.3	4.4	1.6	0.9	**0.0**	2.6	0.4	**0.0**	3.4	0.9	1.4
		p-value	0.706	0.720	0.487	0.315	0.586	**0.003**	0.192	0.640	**0.003**	0.450	0.586	0.344
		1-2	1.1	1.7	3.2	2.3	1.3	0.2	3.6	1.1	1.1	3.2	1.1	1.1
	UK	3+	0.0	3.2	3.2	1.9	0.6	0.6	2.6	0.6	0.6	1.9	1.3	0.6
		p-value	0.201	0.242	0.967	0.781	0.525	0.405	0.549	0.652	0.652	0.429	0.808	0.652
		1-2	0.0	2.1	6.3	0.0	0.0	0.7	1.4	0.0	1.4	2.8	1.4	1.4
C	China	3+	1.6	3.3	5.7	0.6	1.4	0.0	2.1	0.0	0.4	5.1	1.4	1.6
		p-value	0.124	0.466	0.808	0.348	0.150	0.066	0.616	1.000	0.192	0.242	0.974	0.838
		1-2	2.6	5.1	**25.6**	0.0	0.0	0.0	10.3	0.0	0.0	7.7	5.1	**7.7**
	Mexico	3+	2.2	4.6	**8.6**	1.4	1.2	0.0	7.1	0.0	0.2	5.1	2.4	**2.2**
		p-value	0.883	0.873	**0.001**	0.466	0.496	1.000	0.465	1.000	0.802	0.479	0.290	**0.035**
		1-2	0.0	3.8	15.4	0.0	3.8	**3.8**	0.0	0.0	0.0	0.0	0.0	3.8
	Peru	3+	1.8	4.6	11.1	1.5	2.5	**0.0**	4.5	0.0	0.0	5.0	3.3	1.8
		p-value	0.489	0.852	0.499	0.533	0.667	**<0.0001**	0.272	1.000	1.000	0.245	0.347	0.461
		1-2	3.7	2.1	6.3	0.5	1.6	0.5	5.2	0.0	0.0	3.1	1.0	0.5
	Thailand	3+	3.9	1.4	10.0	1.6	2.3	0.0	6.2	0.0	0.2	6.4	1.6	1.4
		p-value	0.901	0.503	0.130	0.271	0.567	0.130	0.654	1.000	0.512	0.099	0.596	0.354
		1-2	3.0	2.1	5.5	**4.9**	0.9	0.3	18.6	0.3	0.0	2.7	1.5	3.0
D	Brazil	3+	2.0	3.3	4.6	**1.7**	0.0	0.0	17.2	0.3	0.0	3.3	0.7	3.3
		p-value	0.398	0.363	0.627	**0.025**	0.096	0.339	0.653	0.955	1.000	0.678	0.303	0.852
		1-2	1.7	0.6	0.0	0.0	0.0	0.6	4.0	0.0	0.6	0.6	0.0	0.6
	India	3+	2.2	0.7	2.2	1.5	0.4	0.0	3.3	0.2	1.3	1.3	0.0	0.4
		p-value	0.724	0.915	0.050	0.103	0.386	0.104	0.638	0.541	0.435	0.435	1.000	0.820
		1-2	0.5	1.1	2.5	1.9	0.5	0.0	8.5	0.3	0.3	2.2	0.8	1.9
	South Africa	3+	1.9	1.5	3.4	2.3	1.1	0.4	7.2	0.4	0.8	2.3	0.8	2.3
		p-value	0.114	0.648	0.490	0.764	0.416	0.242	0.545	0.822	0.388	0.952	0.926	0.764
			**Salty snack food**											
		1-2	9.8	17.1	21.7	14.3	13.0	4.7	24.5	8.1	8.1	21.7	**12.2**	**9.8**
W	USA	3+	6.8	15.5	24.8	16.1	10.6	4.3	23.6	3.7	6.2	21.1	**6.2**	**16.1**
		p-value	0.257	0.654	0.418	0.566	0.417	0.858	0.815	0.060	0.436	0.867	**0.035**	**0.029**
		1-2	**0.6**	2.5	4.6	1.9	**0.6**	0.0	6.3	0.0	0.4	5.0	2.1	1.7
X	Australia	3+	**2.6**	2.6	7.9	2.6	**2.6**	0.0	5.3	0.0	0.0	3.3	2.6	2.6
		p-value	**0.040**	0.935	0.118	0.573	**0.040**	1.000	0.648	1.000	0.426	0.376	0.695	0.453

Cluster	Country	Number of adults ≥ 18	Ant egg	Ant	Bee	Beetle	Caterpillar	Cockroach	Cricket	Fly larvae	Fly	Grasshopper	Mealworm	Wasp
		1-2	0.7	4.2	7.7	0.0	0.0	0.0	2.8	0.0	0.7	8.4	0.7	1.4
	China	3+	1.4	4.1	7.6	2.3	1.0	0.4	3.1	0.2	0.4	6.4	1.2	1.2
		p-value	0.489	0.963	0.971	0.070	0.225	0.445	0.863	0.591	0.661	0.399	0.594	0.877
		1-2	1.2	1.2	5.4	0.0	0.9	0.3	2.4	0.3	0.0	6.0	0.3	1.8
	Japan	3+	1.0	2.4	7.4	0.7	2.0	0.0	2.4	0.7	0.3	7.1	0.7	1.0
		p-value	0.827	0.265	0.294	0.133	0.234	0.348	0.981	0.495	0.290	0.575	0.495	0.410
		1-2	0.5	3.5	3.0	0.7	1.0	0.0	4.0	0.0	0.0	4.7	0.5	1.0
	Russia	3+	1.7	4.4	6.1	0.4	0.9	0.0	5.7	0.0	0.4	4.8	0.4	1.3
		p-value	0.121	0.582	0.058	0.637	0.879	1.000	0.332	1.000	0.187	0.971	0.915	0.720
		1-2	1.6	1.6	4.8	1.6	0.0	0.0	0.0	0.0	1.6	3.2	0.0	0.0
	Spain	3+	0.7	4.1	6.0	1.6	1.6	0.0	4.6	0.4	0.4	3.9	0.4	1.1
		p-value	0.456	0.332	0.693	1.000	0.315	1.000	0.083	0.640	0.178	0.782	0.640	0.414
		1-2	1.5	2.5	4.2	3.2	1.5	0.2	4.2	0.6	1.3	4.8	1.1	1.7
	UK	3+	0.6	1.9	5.8	1.9	1.3	0.0	4.5	0.6	0.0	3.2	1.9	1.9
		p-value	0.425	0.676	0.411	0.429	0.868	0.570	0.871	0.987	0.161	0.397	0.395	0.837
		1-2	5.1	5.1	20.5	0.0	2.6	0.0	17.9	0.0	0.0	15.4	5.1	7.7
Y	Mexico	3+	2.7	6.8	11.7	2.4	3.4	0.0	11.5	0.0	0.0	10.8	3.4	2.7
		p-value	0.381	0.692	0.103	0.332	0.784	1.000	0.230	1.000	1.000	0.381	0.567	0.078
		1-2	0.0	7.7	15.4	0.0	0.0	0.0	0.0	0.0	0.0	3.8	0.0	7.7
	Peru	3+	1.5	6.1	13.9	2.0	3.0	0.0	7.6	0.0	0.0	5.5	3.5	3.3
		p-value	0.533	0.747	0.832	0.470	0.373	1.000	0.145	1.000	1.000	0.722	0.335	0.235
		1-2	2.6	2.1	7.3	1.0	1.0	0.0	10.5	0.0	0.0	9.4	0.0	2.6
	Thailand	3+	4.8	2.7	11.2	1.8	2.7	0.0	12.8	0.0	0.0	11.4	1.6	2.3
		p-value	0.210	0.640	0.141	0.475	0.188	1.000	0.419	1.000	1.000	0.466	0.080	0.798
		1-2	4.6	3.7	5.8	3.7	0.9	0.3	21.6	0.0	0.0	4.6	0.9	4.3
Z	Brazil	3+	3.6	2.3	4.3	2.0	0.3	0.0	18.2	0.0	0.3	4.0	0.3	2.6
		p-value	0.558	0.326	0.396	0.209	0.358	0.339	0.282	1.000	0.299	0.711	0.358	0.269
		1-2	2.9	0.6	1.7	0.6	0.0	0.6	4.6	0.0	1.2	1.7	0.6	0.0
	India	3+	2.0	1.1	1.1	2.4	0.0	0.2	6.1	0.2	1.5	2.6	0.4	2.0
		p-value	0.482	0.554	0.521	0.135	1.000	0.475	0.473	0.541	0.726	0.516	0.820	0.064
		1-2	2.5	1.4	1.6	2.2	0.8	0.0	13.4	0.0	0.3	3.8	0.5	2.7
	South Africa	3+	1.1	1.9	1.5	3.0	1.1	0.4	11.3	0.4	0.4	4.9	1.1	2.3
		p-value	0.227	0.609	0.895	0.516	0.694	0.242	0.432	0.242	0.822	0.513	0.416	0.709

Cluster	Country	Number of adults ≥ 18	Ant egg	Ant	Bee	Beetle	Caterpillar	Cockroach	Cricket	Fly larvae	Fly	Grasshopper	Mealworm	Wasp
			**Meat patty**											
		1-2	10.0	14.7	18.3	13.2	13.6	4.9	21.7	6.6	8.3	19.6	11.9	9.6
W	USA	3+	5.0	11.8	18.6	15.5	11.2	4.3	21.7	6.8	6.8	18.6	9.9	9.9
		p-value	0.050	0.359	0.934	0.464	0.423	0.776	0.998	0.923	0.549	0.786	0.491	0.899
		1-2	0.6	2.3	5.6	2.7	**1.3**	0.2	4.6	0.0	0.2	3.8	2.9	1.5
X	Australia	3+	2.0	2.0	6.6	2.0	**4.6**	0.0	4.6	0.0	0.0	5.3	3.9	2.6
		p-value	0.137	0.812	0.672	0.612	**0.011**	0.575	0.999	1.000	0.575	0.420	0.534	0.340
		1-2	0.7	2.8	10.5	0.0	0.7	0.0	3.5	0.0	0.0	2.1	1.4	1.4
	China	3+	0.8	3.1	6.0	1.4	1.4	0.0	3.7	0.2	0.4	5.3	1.6	0.8
		p-value	0.886	0.863	0.062	0.150	0.489	1.000	0.912	0.591	0.445	0.104	0.838	0.533
		1-2	0.6	0.9	3.9	1.2	0.3	0.0	**3.3**	0.3	0.3	4.8	0.6	1.5
	Japan	3+	2.4	0.7	5.4	1.0	1.4	0.3	**0.3**	0.7	0.3	4.7	0.7	2.0
		p-value	0.063	0.755	0.366	0.827	0.138	0.290	**0.007**	0.495	0.934	0.972	0.905	0.613
		1-2	0.2	2.0	2.5	1.0	1.2	0.0	2.7	0.0	0.0	3.2	0.0	0.5
	Russia	3+	0.0	2.2	4.4	0.9	0.4	0.0	2.2	0.0	0.0	3.1	0.0	1.3
		p-value	0.452	0.874	0.198	0.879	0.315	1.000	0.668	1.000	1.000	0.899	1.000	0.271
		1-2	1.6	0.0	4.8	1.6	0.0	0.0	0.0	0.0	0.0	3.2	1.6	0.0
	Spain	3+	1.1	3.2	3.9	1.6	1.1	0.0	3.5	0.0	0.2	2.8	0.5	1.1
		p-value	0.706	0.152	0.735	1.000	0.414	1.000	0.130	1.000	0.743	0.874	0.317	0.414
		1-2	0.8	2.7	3.8	**2.9**	2.5	0.0	3.6	0.8	0.8	4.2	1.5	1.7
	UK	3+	0.0	1.3	3.2	**0.0**	0.6	0.0	2.6	0.6	1.9	1.3	1.3	0.6
		p-value	0.253	0.306	0.746	**0.031**	0.153	1.000	0.549	0.812	0.260	0.086	0.868	0.345
		1-2	5.1	2.6	**17.9**	2.6	0.0	0.0	15.4	0.0	0.0	7.7	5.1	7.7
Y	Mexico	3+	2.4	5.1	**6.8**	1.9	1.9	0.0	9.6	0.0	0.0	9.3	2.4	2.9
		p-value	0.290	0.483	**0.010**	0.757	0.392	1.000	0.248	1.000	1.000	0.737	0.290	0.097
		1-2	0.0	3.8	11.5	0.0	0.0	0.0	0.0	0.0	0.0	0.0	0.0	3.8
	Peru	3+	1.3	6.0	10.6	1.2	3.8	0.0	6.1	0.0	0.2	5.0	4.0	2.3
		p-value	0.557	0.655	0.880	0.583	0.312	1.000	0.194	1.000	0.842	0.245	0.301	0.618
		1-2	**1.6**	0.5	**4.7**	2.6	1.6	0.0	7.3	0.0	0.5	7.9	**0.0**	2.1
	Thailand	3+	**5.0**	2.7	**9.8**	2.3	2.7	0.0	11.6	0.0	0.2	10.9	**2.5**	1.6
		p-value	**0.042**	0.073	**0.033**	0.798	0.380	1.000	0.104	1.000	0.546	0.237	**0.028**	0.661
		1-2	4.6	2.1	4.0	**4.3**	0.9	0.3	19.8	0.0	0.0	4.3	1.5	3.4
Z	Brazil	3+	3.0	1.7	6.3	**1.0**	0.3	0.3	16.9	0.0	0.3	3.6	0.3	2.0
		p-value	0.297	0.662	0.184	**0.011**	0.358	0.955	0.344	1.000	0.299	0.688	0.124	0.291

Cluster	Country	Number of adults ≥ 18	Ant egg	Ant	Bee	Beetle	Caterpillar	Cockroach	Cricket	Fly larvae	Fly	Grasshopper	Mealworm	Wasp
	India	1-2	0.6	0.6	1.2	0.0	0.0	0.6	5.8	0.0	2.3	1.7	0.0	0.6
		3+	2.0	1.7	1.7	2.2	0.4	0.4	6.6	0.0	1.1	2.0	0.9	1.5
		p-value	0.214	0.270	0.597	0.050	0.386	0.820	0.724	1.000	0.250	0.851	0.219	0.342
	South Africa	1-2	1.1	0.8	2.2	2.2	0.3	0.5	11.0	0.3	0.3	2.2	0.5	0.8
		3+	1.9	2.3	3.0	3.4	0.8	0.4	10.9	0.4	0.8	4.2	0.8	2.3
		p-value	0.410	0.133	0.516	0.358	0.388	0.760	0.995	0.822	0.388	0.156	0.748	0.133

[1]The Mann-Whitney U test was performed to investigate whether the frequency of insect powder selection in each country differed significantly according the number of adults aged 18 or older in participants' household.

[2]Bold highlights means that there is a significant difference (p-value < 0.05).

Table S5. Significant differences in the CATA frequency percentage indicating consumers' willingness to consume specific insect powder according to demographic (number of children under 18 in household; none or any) across the five food types arranged by country clusters.

Cluster	Country	Number of children < 18	Ant egg	Ant	Bee	Beetle	Caterpillar	Cockroach	Cricket	Fly larvae	Fly	Grasshopper	Mealworm	Wasp
			**Muffin or Bread**											
A	USA	None	**8.3**	16.3	24.3	14.6	**10.8**	**3.8**	24.1	6.6	**5.2**	22.6	12.3	9.9
		Any	**14.6**	18.4	27.7	18.4	**16.5**	**11.2**	28.2	8.3	**11.7**	22.8	10.7	12.6
		p-value	**0.015**	0.496	0.361	0.218	**0.046**	**0.0001**	0.268	0.452	**0.003**	0.961	0.563	0.303
B	Australia	None	1.4	2.6	7.9	3.1	1.4	0.5	7.4	0.2	0.7	6.0	2.4	1.0
		Any	0.5	1.9	7.1	2.8	3.3	0.0	7.1	0.0	0.0	4.7	2.4	1.4
		p-value	0.280	0.572	0.733	0.858	0.117	0.316	0.896	0.480	0.219	0.526	0.990	0.599
	Japan	None	**0.4**	0.8	6.6	**1.1**	1.3	**0.2**	2.1	0.4	**0.0**	5.7	0.4	1.5
		Any	**2.5**	2.5	8.9	**3.8**	3.2	**1.9**	3.8	1.9	**1.3**	7.0	1.3	1.3
		p-value	**0.018**	0.099	0.320	**0.022**	0.113	**0.020**	0.239	0.069	**0.014**	0.555	0.246	0.852
	Russia	None	1.2	2.5	4.9	0.9	0.6	0.0	3.1	0.0	0.0	5.8	0.3	1.5
		Any	0.7	3.3	8.6	1.3	1.0	0.0	3.9	0.0	0.7	6.3	0.7	1.3
		p-value	0.464	0.530	0.067	0.637	0.599	1.000	0.549	1.000	0.143	0.825	0.524	0.819
	Spain	None	1.1	2.4	6.5	1.1	0.8	0.3	3.0	0.0	0.0	3.5	0.8	1.4
		Any	1.5	5.0	8.0	1.5	0.8	0.4	4.2	0.0	0.4	3.1	1.1	0.8
		p-value	0.628	0.090	0.474	0.628	0.944	0.811	0.416	1.000	0.237	0.742	0.676	0.483
	UK	None	1.5	2.2	6.2	2.2	0.7	0.2	5.2	**0.2**	0.5	4.0	1.7	2.0
		Any	1.3	4.8	7.0	2.6	2.2	0.4	6.6	**1.7**	1.3	5.2	2.2	1.7
		p-value	0.851	0.078	0.713	0.767	0.122	0.690	0.495	**0.042**	0.271	0.465	0.700	0.827

Cluster	Country	Number of children < 18	Ant egg	Ant	Bee	Beetle	Caterpillar	Cockroach	Cricket	Fly larvae	Fly	Grasshopper	Mealworm	Wasp
C	China	None	1.5	4.5	10.1	0.4	0.4	0.7	4.1	0.0	0.7	5.6	1.9	1.9
		Any	1.1	3.0	15.2	1.4	1.9	0.6	3.0	0.6	0.3	7.2	3.3	1.7
		p-value	0.669	0.342	0.059	0.199	0.084	0.763	0.472	0.224	0.398	0.426	0.268	0.845
	Mexico	None	5.0	5.9	**12.2**	1.3	2.5	0.4	13.4	0.0	0.0	11.8	2.9	2.9
		Any	3.1	8.9	**18.4**	2.0	2.3	0.0	14.5	0.0	0.0	11.7	3.8	3.8
		p-value	0.209	0.167	**0.041**	0.469	0.858	0.201	0.702	1.000	1.000	0.991	0.558	0.558
	Peru	None	**4.0**	8.1	19.8	**3.6**	4.0	0.4	**10.9**	0.0	0.0	**9.3**	**5.6**	2.8
		Any	**0.5**	6.5	14.9	**0.5**	2.1	0.0	**5.0**	0.0	0.0	**4.2**	**2.4**	1.3
		p-value	**0.002**	0.470	0.113	**0.004**	0.154	0.216	**0.005**	1.000	1.000	**0.010**	**0.032**	0.175
	Thailand	None	**2.7**	2.3	16.6	1.7	2.3	0.0	12.0	0.3	0.0	9.6	1.0	2.7
		Any	**7.0**	4.0	11.6	2.7	2.7	0.0	13.4	0.0	0.0	10.9	1.8	1.5
		p-value	**0.012**	0.246	0.067	0.362	0.745	1.000	0.595	0.297	1.000	0.590	0.383	0.316
D	Brazil	None	3.4	3.2	5.4	2.3	0.6	0.3	**30.7**	0.3	0.3	4.6	1.4	2.9
		Any	6.8	3.9	5.0	2.1	0.7	0.4	**38.4**	0.4	0.0	4.6	1.1	3.6
		p-value	0.056	0.605	0.796	0.895	0.829	0.880	**0.041**	0.880	0.371	0.981	0.685	0.622
	India	None	2.7	0.4	2.0	2.4	0.0	0.8	7.8	1.2	2.0	2.7	0.4	2.7
		Any	4.3	0.5	2.4	2.9	0.0	0.8	12.5	1.1	0.8	1.6	0.5	3.2
		p-value	0.321	0.804	0.718	0.664	1.000	0.986	0.063	0.896	0.201	0.319	0.804	0.748
	South Africa	None	2.7	0.9	1.8	3.5	1.5	0.3	24.2	0.3	0.3	3.5	1.5	3.5
		Any	3.1	1.7	3.1	2.7	1.4	0.0	17.9	0.3	0.3	3.4	1.0	2.1
		p-value	0.743	0.353	0.278	0.573	0.917	0.356	0.054	0.916	0.916	0.944	0.621	0.268
			**Cracker**											
A	USA	None	7.8	12.5	18.9	13.4	11.8	**3.5**	22.6	5.4	7.3	19.8	11.8	10.4
		Any	12.6	18.0	24.3	13.6	14.6	**7.8**	29.1	8.3	11.7	22.8	12.1	13.6
		p-value	0.051	0.066	0.116	0.959	0.328	**0.021**	0.077	0.173	0.071	0.384	0.901	0.235
B	Australia	None	1.2	1.4	4.1	1.4	1.2	0.2	5.3	0.0	0.5	3.1	1.4	1.4
		Any	0.5	2.4	6.2	2.8	1.9	0.0	5.2	0.0	0.0	5.7	1.9	2.8
		p-value	0.381	0.397	0.243	0.222	0.484	0.480	0.985	1.000	0.316	0.117	0.661	0.222
	Japan	None	1.3	0.8	4.9	0.2	0.6	0.0	2.7	0.0	0.0	5.3	0.6	1.5
		Any	1.3	1.3	6.4	1.3	1.9	0.0	3.2	0.6	0.0	5.7	0.6	0.6
		p-value	0.997	0.634	0.464	0.094	0.154	1.000	0.777	0.083	1.000	0.831	0.999	0.415
	Russia	None	0.0	2.1	4.3	**0.3**	0.6	0.0	3.4	0.0	0.0	4.0	0.0	0.6
		Any	0.7	1.6	3.6	**2.3**	0.7	0.0	3.6	0.0	0.3	3.3	0.7	1.3
		p-value	0.143	0.646	0.665	**0.026**	0.945	1.000	0.868	1.000	0.302	0.641	0.143	0.366

Cluster	Country	Number of children < 18	Ant egg	Ant	Bee	Beetle	Caterpillar	Cockroach	Cricket	Fly larvae	Fly	Grasshopper	Mealworm	Wasp
		None	1.1	2.2	4.6	1.9	**0.3**	0.0	3.5	0.0	0.0	3.5	1.4	1.6
	Spain	Any	0.8	3.8	5.0	1.1	**1.9**	0.4	5.0	0.0	0.0	4.2	0.8	1.1
		p-value	0.681	0.223	0.843	0.455	**0.037**	0.237	0.375	1.000	1.000	0.668	0.483	0.614
		None	1.0	**0.7**	3.7	1.7	1.0	0.2	4.0	0.5	0.7	2.0	1.5	1.2
	UK	Any	1.3	**3.5**	5.7	2.6	2.2	0.4	4.8	2.2	0.9	4.4	0.9	1.7
		p-value	0.720	**0.011**	0.257	0.459	0.228	0.690	0.628	0.053	0.866	0.086	0.503	0.612
		None	1.1	4.1	6.7	0.7	0.4	0.0	3.4	0.4	0.7	4.9	0.7	2.2
C	China	Any	1.1	4.4	8.6	1.1	1.7	0.0	4.4	0.0	0.6	5.2	1.4	1.4
		p-value	0.987	0.847	0.393	0.648	0.129	1.000	0.500	0.246	0.763	0.823	0.453	0.417
		None	2.1	4.6	8.4	1.3	2.5	0.0	10.5	0.0	0.0	10.9	2.5	2.9
	Mexico	Any	3.1	5.1	11.7	1.3	1.0	0.0	12.5	0.3	0.0	8.4	2.6	3.6
		p-value	0.472	0.788	0.186	0.988	0.145	1.000	0.451	0.438	1.000	0.296	0.982	0.670
		None	**2.4**	6.0	14.1	**3.2**	4.8	0.0	8.1	0.4	0.0	6.9	5.2	3.2
	Peru	Any	**0.3**	5.0	10.2	**0.5**	2.1	0.0	6.8	0.0	0.0	4.7	2.9	1.6
		p-value	**0.012**	0.560	0.138	**0.008**	0.055	1.000	0.554	0.216	1.000	0.252	0.131	0.169
		None	3.0	2.0	10.3	1.3	1.7	0.0	8.0	0.3	0.3	6.6	0.7	0.7
	Thailand	Any	4.6	2.7	9.7	2.7	1.8	0.3	10.0	0.0	0.0	9.1	2.1	1.8
		p-value	0.305	0.542	0.811	0.215	0.877	0.340	0.369	0.297	0.297	0.252	0.123	0.195
		None	2.9	1.7	4.9	**1.7**	0.0	0.0	**15.8**	0.0	**0.0**	3.2	**0.3**	2.3
D	Brazil	Any	2.8	3.2	4.6	**5.0**	0.4	0.7	**22.4**	0.4	**1.8**	3.9	**2.1**	2.1
		p-value	0.990	0.225	0.887	**0.020**	0.266	0.115	**0.033**	0.266	**0.012**	0.605	**0.028**	0.895
		None	2.4	1.6	0.8	0.4	0.0	0.0	**4.7**	0.0	0.8	1.2	0.0	1.2
	India	Any	2.1	1.6	2.7	2.1	0.3	0.5	**9.6**	0.3	0.8	1.3	0.8	1.6
		p-value	0.851	0.979	0.091	0.072	0.412	0.245	**0.024**	0.412	0.986	0.867	0.154	0.664
		None	**2.4**	1.8	1.5	3.5	0.9	0.3	**15.9**	0.3	0.3	4.4	**1.5**	3.8
	South Africa	Any	**0.3**	1.4	2.7	2.1	1.0	0.3	**10.3**	0.3	0.7	2.4	**0.0**	1.7
		p-value	**0.034**	0.693	0.263	0.268	0.852	0.916	**0.039**	0.916	0.477	0.169	**0.038**	0.112
			**Cake**											
		None	8.0	12.5	17.9	12.5	**10.4**	**3.3**	19.3	**4.2**	**7.1**	17.5	9.9	9.4
A	USA	Any	12.1	15.0	23.3	15.5	**16.0**	**6.8**	23.8	**9.2**	**13.1**	19.4	14.1	11.7
		p-value	0.096	0.378	0.112	0.296	**0.043**	**0.046**	0.197	**0.013**	**0.013**	0.548	0.121	0.388
		None	1.7	1.9	4.5	1.4	1.2	0.0	3.6	0.0	1.0	3.8	1.9	1.0
B	Australia	Any	0.5	2.8	6.6	1.9	2.4	0.0	4.7	0.0	0.0	4.3	1.9	1.9
		p-value	0.206	0.454	0.265	0.661	0.266	1.000	0.482	1.000	0.155	0.787	0.991	0.320

Cluster	Country	Number of children < 18	Ant egg	Ant	Bee	Beetle	Caterpillar	Cockroach	Cricket	Fly larvae	Fly	Grasshopper	Mealworm	Wasp
		None	0.4	**0.4**	4.4	**0.2**	1.1	0.2	1.1	0.2	0.0	3.8	0.6	1.5
	Japan	Any	1.3	**2.5**	4.5	**1.9**	0.6	0.6	1.9	0.0	0.6	5.1	0.6	0.6
		p-value	0.246	**0.018**	0.993	**0.020**	0.640	0.413	0.409	0.567	0.083	0.482	0.999	0.415
		None	0.3	2.1	4.3	0.0	0.0	0.0	1.8	0.0	0.0	1.2	0.0	0.3
	Russia	Any	1.0	1.3	3.9	1.0	0.0	0.0	1.6	0.0	0.3	1.3	0.3	1.6
		p-value	0.284	0.427	0.827	0.073	1.000	1.000	0.852	1.000	0.302	0.922	0.302	0.084
		None	0.8	1.4	4.9	1.4	1.1	0.3	2.2	0.5	0.3	4.1	0.8	1.4
	Spain	Any	1.5	3.4	4.2	1.5	0.8	0.0	2.7	0.0	0.0	1.9	1.1	1.1
		p-value	0.402	0.082	0.683	0.862	0.681	0.401	0.687	0.233	0.401	0.127	0.676	0.814
		None	**0.2**	1.2	2.5	1.5	1.0	0.2	2.5	0.5	**0.2**	2.5	1.2	1.0
	UK	Any	**1.7**	3.5	4.4	3.5	1.3	0.4	4.8	1.7	**2.2**	3.5	0.9	0.9
		p-value	**0.042**	0.057	0.198	0.102	0.720	0.690	0.121	0.121	**0.016**	0.470	0.668	0.879
		None	1.1	3.0	6.0	0.4	**0.0**	0.4	1.9	0.0	0.7	4.5	0.7	1.5
C	China	Any	1.4	3.0	5.8	0.6	**1.9**	0.0	1.9	0.0	0.6	4.7	1.9	1.7
		p-value	0.773	0.970	0.929	0.748	**0.022**	0.246	0.952	1.000	0.763	0.898	0.215	0.871
		None	1.3	4.2	7.1	0.8	1.7	0.0	5.9	0.0	0.4	5.0	1.7	3.4
	Mexico	Any	2.8	4.8	11.2	1.5	0.8	0.0	8.2	0.0	0.0	5.4	3.1	2.0
		p-value	0.203	0.709	0.093	0.454	0.289	1.000	0.287	1.000	0.201	0.864	0.286	0.308
		None	2.0	6.0	12.9	**2.8**	**4.4**	0.0	**6.5**	0.0	0.0	**6.9**	4.8	2.8
	Peru	Any	1.6	3.7	10.2	**0.5**	**1.3**	0.3	**2.9**	0.0	0.0	**3.4**	2.1	1.3
		p-value	0.678	0.164	0.297	**0.018**	**0.015**	0.422	**0.031**	1.000	1.000	**0.047**	0.055	0.175
		None	3.0	1.0	9.0	1.0	1.7	0.3	6.6	0.0	0.0	5.3	1.0	1.0
	Thailand	Any	4.6	2.1	8.8	1.5	2.4	0.0	5.2	0.0	0.3	5.5	1.8	1.2
		p-value	0.305	0.257	0.946	0.559	0.498	0.297	0.432	1.000	0.340	0.932	0.383	0.794
		None	2.0	2.9	4.6	2.9	0.3	0.0	**14.9**	0.0	0.0	3.4	0.9	2.6
D	Brazil	Any	3.2	2.5	5.7	3.9	0.7	0.4	**21.7**	0.7	0.0	2.5	1.4	3.9
		p-value	0.343	0.774	0.529	0.467	0.442	0.266	**0.027**	0.115	1.000	0.490	0.503	0.343
		None	2.0	0.8	1.2	0.4	0.8	0.0	2.7	0.0	0.8	1.2	0.0	0.4
	India	Any	2.1	0.5	1.9	1.6	0.0	0.3	4.0	0.3	1.3	1.1	0.0	0.5
		p-value	0.886	0.696	0.500	0.157	0.086	0.412	0.404	0.412	0.522	0.896	1.000	0.804
		None	0.9	1.2	2.1	2.4	0.9	0.0	**10.3**	0.3	0.3	2.1	1.2	2.4
	South Africa	Any	1.4	1.4	3.8	1.7	0.7	0.3	**5.2**	0.3	0.7	2.4	0.3	1.7
		p-value	0.560	0.829	0.198	0.573	0.782	0.282	**0.017**	0.916	0.477	0.773	0.239	0.573

Cluster	Country	Number of children < 18	Ant egg	Ant	Bee	Beetle	Caterpillar	Cockroach	Cricket	Fly larvae	Fly	Grasshopper	Mealworm	Wasp
			**Salty snack food**											
W	USA	None	7.8	**13.7**	20.5	14.2	**10.4**	3.5	22.6	5.7	**5.4**	21.7	10.4	10.8
		Any	11.7	**22.8**	26.7	16.0	**16.5**	6.8	27.7	9.7	**12.1**	21.4	11.2	12.6
		p-value	0.113	**0.004**	0.082	0.536	**0.029**	0.067	0.168	0.062	**0.003**	0.923	0.764	0.512
X	Australia	None	1.2	2.6	5.3	1.9	**0.2**	0.0	6.0	0.0	0.5	4.3	2.1	1.2
		Any	0.9	2.4	5.7	2.4	**2.8**	0.0	6.2	0.0	0.0	5.2	2.4	3.3
		p-value	0.783	0.848	0.820	0.702	**0.003**	1.000	0.923	1.000	0.316	0.605	0.859	0.066
	China	None	0.7	4.5	6.0	0.7	0.4	0.4	3.0	0.0	0.7	6.7	0.7	1.5
		Any	1.7	3.9	8.8	2.5	1.1	0.3	3.0	0.3	0.3	6.9	1.4	1.1
		p-value	0.313	0.704	0.180	0.100	0.307	0.833	0.970	0.391	0.398	0.926	0.453	0.669
	Japan	None	0.8	1.9	6.8	0.4	1.5	0.2	2.1	**0.0**	0.0	5.9	0.4	1.5
		Any	1.9	1.3	5.1	0.0	1.3	0.0	3.2	**1.9**	0.6	8.3	0.6	1.3
		p-value	0.271	0.603	0.458	0.416	0.852	0.567	0.447	**0.003**	0.083	0.299	0.737	0.852
	Russia	None	0.6	3.4	4.0	**0.0**	0.6	0.0	3.7	0.0	0.0	4.9	0.0	0.9
		Any	1.3	4.3	4.3	**1.3**	1.3	0.0	5.6	0.0	0.3	4.6	1.0	1.3
		p-value	0.366	0.555	0.856	**0.038**	0.366	1.000	0.253	1.000	0.302	0.859	0.073	0.637
	Spain	None	0.5	**2.4**	5.7	1.4	0.8	0.0	3.8	0.3	0.5	3.3	0.3	0.8
		Any	1.1	**5.7**	6.1	1.9	2.3	0.0	4.6	0.4	0.4	4.6	0.4	1.1
		p-value	0.403	**0.034**	0.834	0.587	0.125	1.000	0.630	0.811	0.773	0.395	0.811	0.676
	UK	None	0.7	1.7	**3.2**	2.2	1.2	0.2	**3.0**	0.5	0.5	3.7	1.0	1.7
		Any	2.2	3.5	**7.0**	3.9	1.7	0.0	**6.6**	0.9	1.7	5.7	1.7	1.7
		p-value	0.122	0.167	**0.031**	0.223	0.612	0.452	**0.034**	0.571	0.121	0.257	0.420	1.000
Y	Mexico	None	3.4	5.9	10.5	0.8	3.8	0.0	12.6	0.0	0.0	10.5	2.9	2.1
		Any	2.6	7.1	13.3	3.1	3.1	0.0	11.5	0.0	0.0	11.5	3.8	3.6
		p-value	0.555	0.539	0.306	0.067	0.626	1.000	0.673	1.000	1.000	0.706	0.558	0.296
	Peru	None	2.4	6.5	16.5	3.2	4.4	0.0	9.3	0.0	0.0	7.3	4.4	4.4
		Any	0.8	6.0	12.3	1.0	1.8	0.0	6.0	0.0	0.0	4.2	2.6	2.9
		p-value	0.092	0.827	0.135	0.051	0.056	1.000	0.126	1.000	1.000	0.096	0.215	0.299
	Thailand	None	3.7	2.7	12.0	1.3	1.7	0.0	**9.3**	0.0	0.0	9.6	1.0	2.7
		Any	4.6	2.4	8.2	1.8	2.7	0.0	**14.6**	0.0	0.0	11.9	1.2	2.1
		p-value	0.569	0.858	0.117	0.621	0.362	1.000	**0.042**	1.000	1.000	0.370	0.794	0.664
Z	Brazil	None	3.2	3.2	5.2	2.0	0.3	0.0	**16.9**	0.0	0.0	4.6	0.6	3.4
		Any	5.3	2.8	5.0	3.9	1.1	0.4	**23.8**	0.0	0.4	3.9	0.7	3.6
		p-value	0.171	0.825	0.921	0.153	0.221	0.266	**0.031**	1.000	0.266	0.681	0.829	0.935

Cluster	Country	Number of children < 18	Ant egg	Ant	Bee	Beetle	Caterpillar	Cockroach	Cricket	Fly larvae	Fly	Grasshopper	Mealworm	Wasp
	India	None	2.7	1.6	1.2	1.2	0.0	0.0	4.3	0.0	0.4	2.7	0.4	0.8
		Any	1.9	0.5	1.3	2.4	0.0	0.5	6.6	0.3	2.1	2.1	0.5	1.9
		p-value	0.461	0.189	0.867	0.273	1.000	0.245	0.215	0.412	0.072	0.618	0.804	0.264
	South Africa	None	2.1	2.1	1.5	3.5	1.2	0.3	**15.6**	0.3	0.3	4.4	1.2	3.5
		Any	1.7	1.0	1.7	1.4	0.7	0.0	**8.9**	0.0	0.3	4.1	0.3	1.4
		p-value	0.752	0.301	0.808	0.085	0.527	0.356	**0.011**	0.356	0.916	0.853	0.239	0.085
			**Meat patty**											
W	USA	None	7.8	12.7	17.5	14.4	**10.6**	**3.5**	19.8	5.7	**6.1**	19.1	10.8	9.9
		Any	10.7	16.5	20.4	12.6	**18.0**	**7.3**	25.7	8.7	**11.7**	19.9	12.6	9.2
		p-value	0.228	0.201	0.373	0.547	**0.010**	**0.039**	0.092	0.147	**0.016**	0.812	0.512	0.786
X	Australia	None	1.0	2.4	5.3	2.9	1.7	0.2	4.5	0.0	0.2	4.1	3.3	1.9
		Any	0.9	1.9	7.1	1.9	2.8	0.0	4.7	0.0	0.0	4.3	2.8	1.4
		p-value	0.995	0.694	0.350	0.467	0.329	0.480	0.908	1.000	0.480	0.902	0.737	0.660
	China	None	1.1	3.7	7.8	1.1	0.7	0.0	4.1	0.0	0.4	4.1	1.1	1.5
		Any	0.6	2.5	6.4	1.1	1.7	0.0	3.3	0.3	0.3	5.0	1.9	0.6
		p-value	0.429	0.367	0.471	0.987	0.313	1.000	0.602	0.391	0.833	0.608	0.420	0.231
	Japan	None	1.5	**0.2**	4.9	0.8	0.6	0.0	1.7	**0.0**	0.2	4.2	0.6	1.9
		Any	1.3	**2.5**	3.8	1.9	1.3	0.6	2.5	**1.9**	0.6	6.4	0.6	1.3
		p-value	0.852	**0.004**	0.590	0.271	0.435	0.083	0.497	**0.003**	0.413	0.276	0.999	0.603
	Russia	None	0.0	1.5	3.1	0.9	0.6	0.0	2.5	0.0	0.0	3.1	0.0	0.6
		Any	0.3	2.6	3.3	1.0	1.3	0.0	2.6	0.0	0.0	3.3	0.0	1.0
		p-value	0.302	0.334	0.874	0.933	0.366	1.000	0.888	1.000	1.000	0.874	1.000	0.599
	Spain	None	1.1	**1.6**	3.5	0.8	0.8	0.0	2.7	0.0	0.3	3.8	0.5	1.1
		Any	1.1	**4.6**	4.6	2.7	1.1	0.0	3.8	0.0	0.0	1.5	0.8	0.8
		p-value	0.946	**0.029**	0.508	0.066	0.676	1.000	0.439	1.000	0.401	0.091	0.733	0.681
	UK	None	0.2	1.7	**2.2**	2.2	**1.0**	0.0	2.7	**0.2**	0.7	2.7	0.7	1.7
		Any	1.3	3.5	**6.1**	2.2	**3.9**	0.0	4.4	**1.7**	1.7	4.8	2.6	0.9
		p-value	0.108	0.167	**0.013**	0.961	**0.013**	1.000	0.276	**0.042**	0.251	0.176	0.057	0.376
Y	Mexico	None	3.4	3.8	5.9	1.7	1.7	0.0	9.2	0.0	0.0	9.7	2.1	2.9
		Any	2.0	5.6	8.4	2.0	1.8	0.0	10.5	0.0	0.0	8.9	2.8	3.3
		p-value	0.308	0.304	0.241	0.749	0.923	1.000	0.623	1.000	1.000	0.757	0.586	0.795
	Peru	None	**2.4**	6.9	13.3	**2.4**	4.8	0.0	8.1	0.0	0.4	**7.7**	5.6	3.2
		Any	**0.5**	5.2	8.9	**0.3**	2.9	0.0	4.5	0.0	0.0	**2.9**	2.6	1.8
		p-value	**0.038**	0.399	0.080	**0.012**	0.201	1.000	0.060	1.000	0.216	**0.006**	0.053	0.263

Cluster	Country	Number of children < 18	Ant egg	Ant	Bee	Beetle	Caterpillar	Cockroach	Cricket	Fly larvae	Fly	Grasshopper	Mealworm	Wasp
	Thailand	None	3.0	1.7	10.3	2.0	2.0	0.0	11.0	0.0	0.3	8.0	1.0	1.7
		Any	4.9	2.4	6.4	2.7	2.7	0.0	9.7	0.0	0.3	11.9	2.4	1.8
		p-value	0.230	0.498	0.075	0.542	0.542	1.000	0.611	1.000	0.952	0.105	0.170	0.877
Z	Brazil	None	3.2	2.0	4.9	2.0	0.3	0.3	**14.9**	0.0	0.0	4.0	0.6	2.3
		Any	4.6	1.8	5.3	3.6	1.1	0.4	**22.8**	0.0	0.4	3.9	1.4	3.2
		p-value	0.337	0.837	0.791	0.232	0.221	0.880	**0.011**	1.000	0.266	0.951	0.276	0.484
	India	None	0.8	2.0	1.6	2.0	0.0	0.4	**3.9**	0.0	1.2	2.4	1.2	2.0
		Any	2.1	1.1	1.6	1.3	0.5	0.5	**8.0**	0.0	1.6	1.6	0.3	0.8
		p-value	0.186	0.352	0.979	0.534	0.245	0.804	**0.040**	1.000	0.664	0.495	0.158	0.201
	South Africa	None	1.5	1.5	2.4	3.2	0.6	0.3	12.1	0.3	0.3	2.9	1.2	1.5
		Any	1.4	1.4	2.7	2.1	0.3	0.7	9.6	0.3	0.7	3.1	0.0	1.4
		p-value	0.917	0.917	0.758	0.362	0.656	0.477	0.322	0.916	0.477	0.917	0.063	0.917

[1]The Mann-Whitney U test was performed to investigate whether the frequency of insect powder selection in each country differed significantly according the number of children aged under 18 in participants' household.

[2]Bold highlights means that there is a significant difference (p-value < 0.05).
